# Anti-Electrostatic Pi-Hole Bonding: How Covalency Conquers Coulombics

**DOI:** 10.3390/molecules27020377

**Published:** 2022-01-07

**Authors:** Frank Weinhold

**Affiliations:** Theoretical Chemistry Institute and Department of Chemistry, University of Wisconsin-Madison, Madison, WI 53705, USA; weinhold@chem.wisc.edu

**Keywords:** sigma hole, pi hole, Bürgi–Dunitz angle, *n*-π* donor-acceptor interaction, intermolecular interactions, classical electrostatics, Hellmann–Feynman theorem, metastable species, natural bond orbital, natural resonance theory

## Abstract

Intermolecular bonding attraction at π-bonded centers is often described as “electrostatically driven” and given quasi-classical rationalization in terms of a “pi hole” depletion region in the electrostatic potential. However, we demonstrate here that such bonding attraction also occurs between closed-shell ions of *like* charge, thereby yielding locally stable complexes that sharply violate classical electrostatic expectations. Standard DFT and MP2 computational methods are employed to investigate complexation of simple pi-bonded diatomic anions (BO^−^, CN^−^) with simple atomic anions (H^−^, F^−^) or with one another. Such “anti-electrostatic” anion–anion attractions are shown to lead to robust metastable binding wells (ranging up to 20–30 kcal/mol at DFT level, or still deeper at dynamically correlated MP2 level) that are shielded by broad predissociation barriers (ranging up to 1.5 Å width) from long-range ionic dissociation. Like-charge attraction at pi-centers thereby provides additional evidence for the dominance of 3-center/4-electron (3c/4e) *n*_D_-π*_AX_ interactions that are fully analogous to the *n*_D_-σ*_AH_ interactions of H-bonding. Using standard keyword options of natural bond orbital (NBO) analysis, we demonstrate that both *n*-σ* (sigma hole) and *n*-π* (pi hole) interactions represent simple variants of the essential resonance-type donor-acceptor (Bürgi–Dunitz-type) attraction that apparently underlies *all* intermolecular association phenomena of chemical interest. We further demonstrate that “deletion” of such π*-based donor-acceptor interaction *obliterates* the characteristic Bürgi–Dunitz signatures of pi-hole interactions, thereby establishing the *unique* cause/effect relationship to short-range covalency (“charge transfer”) rather than envisioned Coulombic properties of unperturbed monomers.

## 1. Introduction

The concept that pi-bonded centers exert a characteristic form of directional binding traces back to pioneering statistical analyses of the Cambridge Structural Database (CSD) by Bürgi and Dunitz [1,2], who recognized the general propensity for nucleophilic groups to adopt a particular orientation (now termed the “Bürgi–Dunitz angle” [3]) with respect to the pi-bond of ketones or aldehydes. The broader implications of such pi-type stabilizing interactions in protein chemistry (complementing the well-known sigma-type interactions of hydrogen bonding) were subsequently explored by Raines and coworkers [4,5,6,7,8] with combined CSD, NMR, computational and natural bond orbital (NBO) methods [9,10]. Results of these studies consistently affirm the conceptual aptness of the “*n*-π*” orbital picture of Bürgi–Dunitz interaction (lone pair *n*_donor_ of the nucleophilic e-donor with the π*_acceptor_ valence antibond of the pi-bonded e-acceptor moiety), consistent with the analogous *n*-σ* picture of H-bonding interactions [11,12]. General consistency of qualitative Bürgi–Dunitz conceptions with corresponding *n*π* orbital-level descriptors is now well recognized in a broad range of chemical and biochemical phenomena [13,14]. 

As halogen bonds and other (pnicogen, tetrel, …) analogs of H-bonding [15,16] were increasingly recognized as significant features of intermolecular interactions, the alternative “sigma-hole” picture of such interactions was introduced by Politzer and coworkers [17,18]. This picture focuses on the electrostatic potential (ESP) and its characteristic depletion region along the C-X bonding axis that suggests a quasi-classical electrostatic rationale for directional attraction to lone pairs of an incoming nucleophile. An analogous “pi-hole” rationale can be developed for the out-of-plane attractions to nucleophiles around C=X pi-bonds, suggesting that all such forms of “non-covalent” bonding are driven by quasi-classical attractions of electron-rich species to positive (hole-like) regions of the ESP [19]. A variety of computational studies have lent support to such “electrostatically driven” conceptions of pi-hole interactions [20,21,22,23,24,25,26,27,28,29].

However, the orbital-level NBO donor-acceptor rationalization of supramolecular bonding is essentially *quantal* in nature [30,31], with no intrinsic dependence on secondary electrostatic or other classical forces. In the case of A−H∙∙∙B hydrogen bonding, the *n*_B_-σ*_AH_ 2-electron stabilizing interaction [3] corresponds formally to resonance-type mixing with the alternative A^−^∙∙∙H−B^+^ charge-transfer bonding pattern. Such resonance-type mixing leads to *fractional* bond orders *b*_AH_, *b*_BH_ that continuously vary over an allowed range of values satisfying a form of total bond-order conservation law [32], *b*_AH_ + *b*_BH_ = 1. The symmetry of such bond-conserving relationships in turn reflects the essential *identity* of quantum covalency forces that underlie both majority “covalent bond” and minority “hydrogen bond” linkages. A recent study [33] demonstrates more generally that NBO-based signatures of H-bonding are also faithfully exhibited by other X-bonding phenomena (specifically, of halogen and pnicogen type) in a manner that is remarkably *independent* of polarity reversals or geometrical variations that should be expected to remove the bonding if classical-type electrostatics were the authentic driving force. Such lines of evidence indicate that classical electrostatic interactions provide at most a *modulating* influence on the underlying resonance *n*-σ* features of H-bonding and related X-bonding interactions of chemical interest, much as heteroatom substitutions alter the strength, but not the characteristic signatures, of benzenoid aromaticity. 

The secondary role of electrostatics in H-bonding is demonstrated still more directly by proliferating theoretical and experimental evidence [34,35,36,37,38,39,40,41,42,43,44,45,46,47,48,49,50,51,52,53,54,55,56,57,58,59,60,61,62,63,64,65,66,67,68] for “anti-electrostatic” H-bonds between closed-shell ions of *like* charge, in strong contradiction to expectations of classical electrostatics. In particular, IR studies of functionalized ionic liquids provide striking evidence for polyionic H-bonded clusters (e.g., tetrameric^4+^ hydroxyimidazolium species [37]) that closely match the cooperative structural and spectroscopic properties of corresponding neutral alcohol clusters [69]. The demonstrated ability of polyionic H-bond clusters to defy Coulomb explosion [70] testifies to the primacy of exponential exchange-type forces of short-range quantum covalency over the long-range power-law forces of classical electrostatics in general X-bonding phenomena. 

The present work builds on these previous results to investigate whether similar anti-electrostatic defiance of classical electrostatic expectations is exhibited by pi-hole interactions between like-charged ions. For this purpose, we employ standard density functional theory (DFT) and 2nd-order Møller–Plesset (MP2) methods to study various like-charge pi-hole complexes between simple closed-shell monatomic (H^−^, F^−^) and pi-bonded diatomic (BO^−^, CN^−^) anions. As described below, the results demonstrate that *n*-π* and *n*-σ* interactions are comparable in this respect and reinforce one another in complementary manner to surprisingly *deepen* the metastable potential wells and *broaden* the predissociation barriers that were previously found for *n*-σ* complexes. 

## 2. Methods

All DFT calculations were carried out at B3LYP/6-311++G** level, for strict consistency with numerous previous examples in NBO literature. Possible effects of London dispersion and other higher-order dynamical electron correlation corrections were addressed with comparative Møller–Plesset calculations at MP2/6-311++G** level in the *Gaussian 16* program implementation [71,72]. NBO descriptors, natural resonance theory (NRT) bond orders [73,74], and associated orbital graphics were obtained with *NBO 7.0* [75,76] as incorporated in the *NBOPro7@*Jmol [77] utility program. 

For all considered anion–anion pairs, 1-D potential energy surface (PES) relaxed-scan plots were obtained with respect to the shortest interatomic distance (*R*_i∙∙∙j_) between monomer anions, using the opt = modredundant keyword option. All points were checked with the stable = opt keyword for electronic stability, and all points of open-shell (partial diradical singlet) character were evaluated with corresponding unrestricted (UB3LYP, UMP2) methods and general “different Lewis structures for different spin”^4^ NBO/NRT analysis [9,10]. Vibrational analysis was performed for each calculated stationary point to assure proper vibrational stability or saddle-point character. *G16* input files with complete geometry information (and 〈S^2^〉 values, if non-zero) for each stationary and non-stationary point are included in Supplementary Information (SI). 

## 3. Results

### 3.1. Structural, Energetic, and NBO Properties of Various Binary Complexes of H^−^, F^−^, BO^−^, CN^−^ Anions

As simple examples drawn from a selected set of main-group monatomic (H^−^, F^−^) and pi-bonded diatomic (BO^−^, CN^−^) anions, we focus on complexes with the more electropositive end (B, C) of each diatomic bonding pattern (:B≡O:, :C≡N:) as principal coordinating center with the other anion. Of the seven such possible anion–anion complexes, six combinations (all but the imaginable F^−^∙∙∙CN^−^ complex) were found to yield locally stable short-range equilibrium structures when brought into the sub-van der Waals range of separations where exchange-type donor-acceptor interactions are expected to become appreciable. Each such metastable binding well is found to be shielded inside an imposingly high and broad predissociation barrier (capped by a well-defined transition-state structure) that is expected to confer long-lived protection from Coulomb explosion to the long-range ionic dissociation limit. Geometrical and electronic features of these binding wells and shielding barriers will now be compared to exhibit the common Bürgi–Dunitz-type *n*-π* aspect of their surprising metastability. 

**Figure 1:**
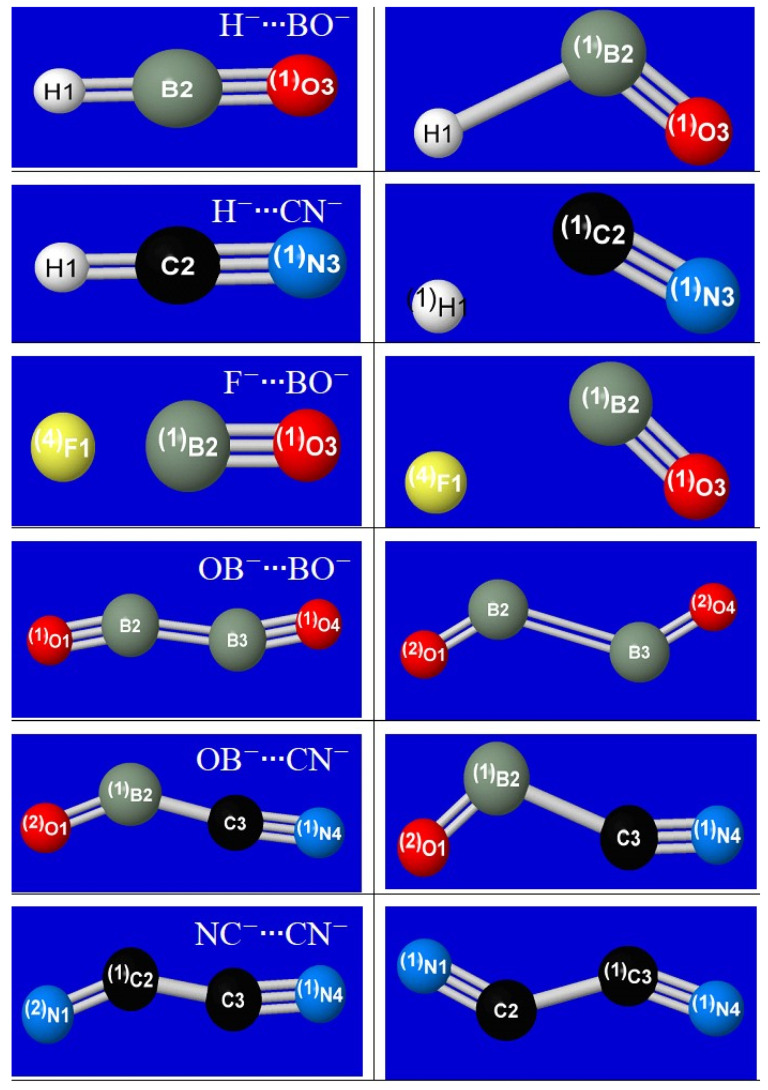
Computed B3LYP/6-311++G** equilibrium (**left**) and transition-state (**right**) structures for like-charge pi-hole complexes (from top to bottom row: H^−^∙∙∙BO^−^, H^−^∙∙∙CN^−^, F^−^∙∙∙BO^−^, OB^−^∙∙∙BO^−^, OB^−^∙∙∙CN^−^, NC^−^∙∙∙CN^−^), showing nominal NLS (natural Lewis structure) bonding pattern (for α-spin, if biradicaloid) for each species at the depicted geometry.

The coordination geometries for each of the six metastable anion–anion complexes (H^−^∙∙∙BO^−^, H^−^∙∙∙CN^−^, F^−^∙∙∙BO^−^, OB^−^∙∙∙BO^−^, OB^−^∙∙∙CN^−^, NC^−^∙∙∙CN^−^) are displayed in the successive rows of Figure 1. The panels of each row show both the equilibrium (left) and transition-state (right) geometry, each with the natural Lewis structure (NLS) pattern of bonds (sticks) and lone pairs (pre-superscripts) at the corresponding stationary-state geometry. Except for F^−^∙∙∙BO^−^ (3rd row), the formal NLS depiction of each equilibrium structure exhibits dative bond formation (or even *double*-bond formation for H^−^∙∙∙BO^−^, H^−^∙∙∙CN^−^, consistent with the two available π* acceptor orbitals available to the *n*_H_ donor orbital) that suggests the remarkable strength of anion–anion binding energies in such *n*-π* complexes compared to known *n*-σ* species. 

Table 1 summarizes details of the equilibrium geometry of each anion–anion complex in terms of interatomic distances (*R*_ij_) and bending angles (*A*_ijk_). As shown in Figure 1, the equilibrium and transition-state structures for diatomic anion–anion complexation are found to have planar *C*_s_-symmetric geometry, with characteristic trans-like kinking resembling that commonly found in analogous neutral species. Despite the fierce Coulombic opposition, the equilibrium inter-monomer distances (*R*_23_) are found to lie within the range of ordinary chemical bonding interactions, consistent with the NLS depictions of Figure 1.

Quantitative details of the binding energies (Δ*E*_bind_), dissociative energy release (Δ*E*_dissoc_), equilibrium vibrational frequencies (ν_i_^(eq)^), and transition saddle-point frequency (ν_1_^‡^) for each anion–anion complex are summarized in Table 2, comparing DFT (left) with corresponding MP2 (right) values for each species. As anticipated in NLS depictions, the calculated binding energies tend to be significantly higher than those for known *n*-σ* complexes, ranging toward values that would be considered representative of conventional dative bonds [viz., 27 kcal/mol at DFT level (or 37 kcal/mol at MP2 level) for H^−^∙∙∙CN^−^]. These values are remarkable in view of the huge exothermicities for ionic dissociation (in the range 80–150 kcal/mol), warranting designation of such metastable species as “energetic materials”.

**Figure 2:**
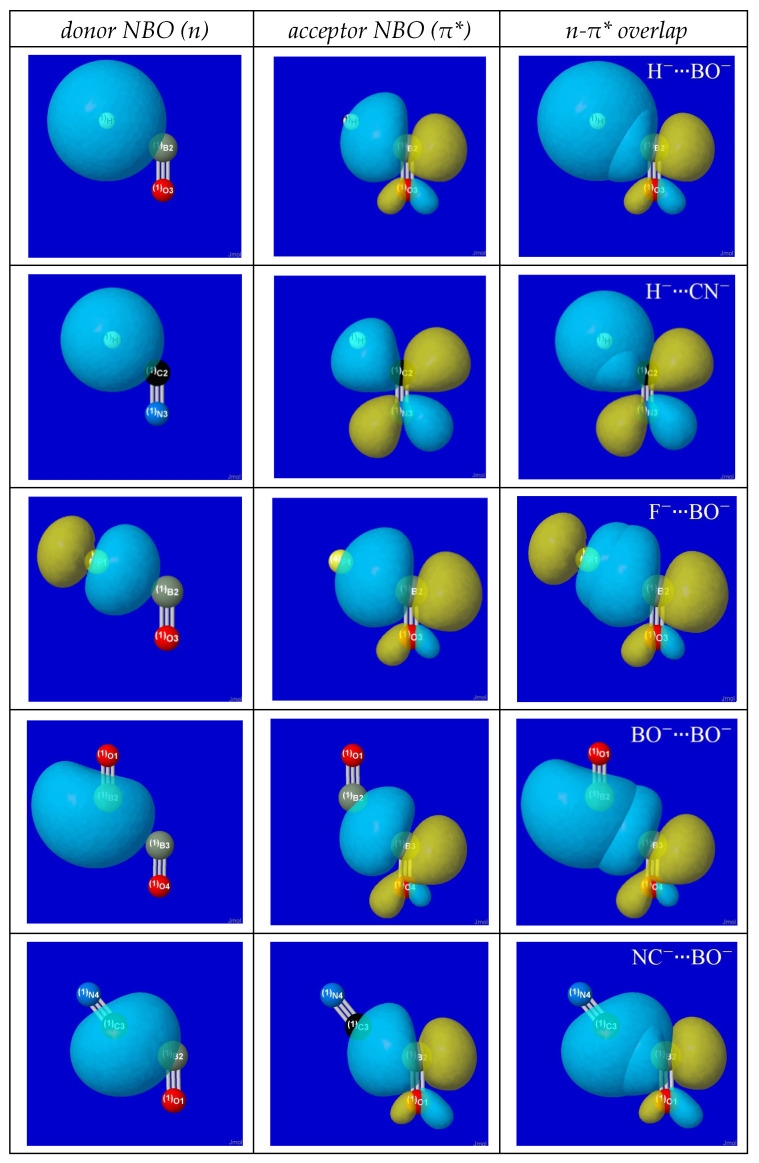

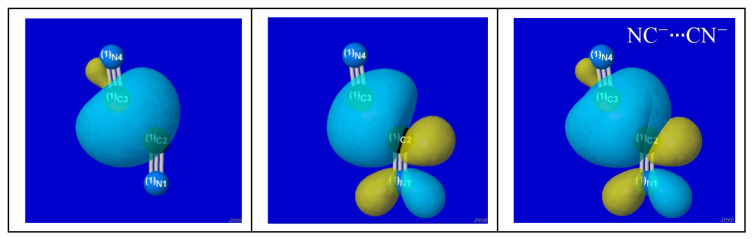
(P)NBO *n*-π* orbital interaction diagrams in the transition-state species of Figure 1, showing donor lone pair (*n*, **left**), acceptor antibond (π*, **center**), and intermolecular *n*π* overlap (**right**) that is maximized in Bürgi–Dunitz approach geometry.

The aptness of “*n*-π*” description of anion–anion attraction can be seen in the pre-NBO overlap diagrams for primary *n*_donor_-π*_acceptor_ interactions at transition-state geometry in Figure 2. The first two panels of each row display the donor lone pair (*n*) and acceptor pi-antibond (π*) NBO for the *n*-π* orbital overlap diagram in the right panel. In each case, the approach geometry achieves maximum overlap of monomer donor and acceptor orbitals (and thus, maximum *n*_donor_-π*_acceptor_ stabilization, according to Mulliken-type precepts [78]) in the transition-state region where quantum covalency forces gain ascendency over powerful long-range Coulombic repulsion. The close relationship of the NBO *n*-π* orbital overlap diagrams to original Bürgi–Dunitz conceptions is evident.

**Figure 3:**
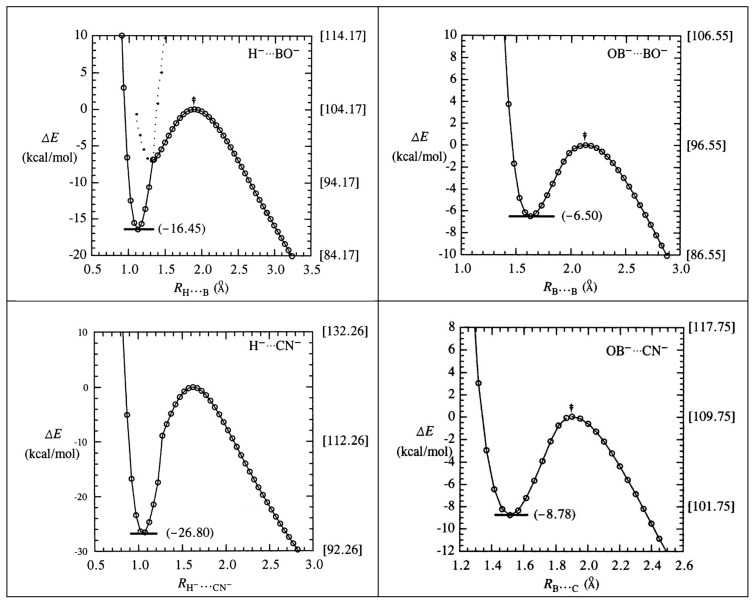

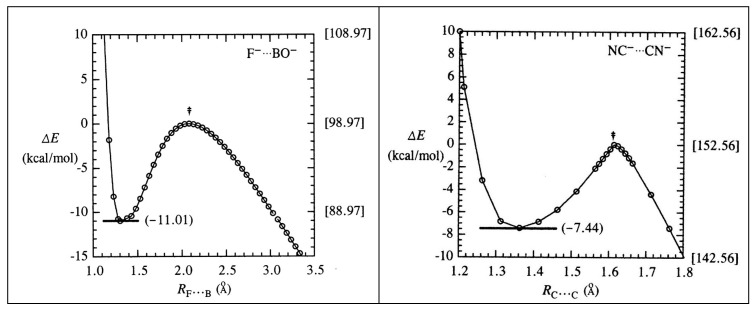
Relaxed-scan energy profiles for metastable binding wells of the six pihole complexes of Figure 1, showing the parenthesized well depths (**left** scale) and bracketed dissociation energies (**right** scale) to the long-range dissociation limit.

The DFT relaxed-scan potential curves for each pi-hole complex are shown in the panels of Figure 3. In each case, the scan extends sufficiently toward the long-range dissociation limit to include the full width of the predissociation barrier (viz., ca. 1.8 Å for H^−^∙∙∙BO^−^ but only 0.4 Å for NC^−^∙∙∙CN^−^). The bracketed values at the right edge of each plot measure the metastable “stored” energies with respect to the infinite separation limit (cf. Δ*E*_dissoc_ of Table 2), whereas values at the left measure well-depth below the transition state (cf. Δ*E*_bind_ of Table 2). The potential curves in each plot are seen to exhibit relatively gentle outer slope (asymptotically, Coulombic *R*^−1^ repulsion) vs. steep inner slope (exponential-type steric repulsion) on either side of the binding well.

For the H^−^∙∙∙BO^−^ species (upper-left panel), informative additional detail is shown for the curve-crossing near *R*_H∙∙∙B_ ≈ 1.3 Å. As shown by the dotted extensions, the crossing marks a juncture between a relatively shallow outer well (ca. 7 kcal/mol) to the much deeper final well (ca. 16 kcal/mol). At this same juncture, the geometry also switches from bent (“*n*-π*”-like; cf. transition-state geometry of Figure 1) to the linear (“*n*-σ*”-like) geometry of the final equilibrium species. A similar geometry switch from bent to linear is seen in each of the three atom-diatom complexes (see top three rows of Figure 1), whereas the diatom-diatom complexes fail to achieve this transition and instead remain in a shallower binding well of kinked geometry.

For H^−^∙∙∙BO^−^ and other atom-diatom complexes it therefore appears that the dominant *n*-π* orbital interaction of the transition-state approach region serves merely as a catalyst or “gateway” to the final linear geometry, where *n*-π* orbital interactions are forbidden by symmetry. However, in actuality this symmetry change can occur in more subtle fashion through rehybridization of the long-range diatomic π* orbital to an acceptor orbital of lower symmetry. Rehybridized distortion of the nominal “π*” acceptor NBO is already conspicuous in the transition-state of NC^−^∙∙∙CN^−^ (lowest row of Figure 2), where one lobe of the erstwhile π* orbital appears enlarged and distended toward the incoming lone pair of the donor anion to increasingly resemble the sp^λ^ hybrid of a directed sigma bond. Further details of atomic rehybridization are beyond the scope of present discussion, but one can see in a general way that an unsaturated species with two (or more) valence acceptor orbitals of different shape must afford greater quantum mechanical flexibility (and deeper binding wells; cf. Table 2) than a saturated species with only one such acceptor orbital.

**Figure 4:**
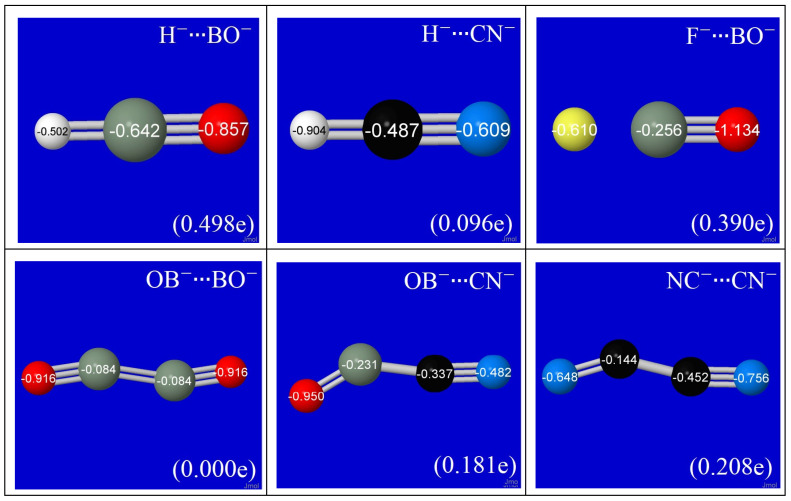
NPA atomic charges for equilibrium species of Figure 1, showing net charge transfer Δ*Q*_CT_ (in parentheses) from donor to acceptor monomer in each species.

For the final equilibrium species, Figure 4 displays the atomic charge distributions and parenthesized net charge transfer Δ*Q*_CT_ between monomers in each pi-hole complex. As suggested by the NLS diagrams of Figure 1, the atomic charge distributions differ significantly from those of the initial monomer anions (e.g., nearly 0.5e charge transfer from H^−^ to BO^−^ in the H^−^∙∙∙BO^−^ complex). Even when *net* Δ*Q*_CT_ appears negligible (as, e.g., in *C*_2_-symmetric OB^−^∙∙∙BO^−^), the NBO populations reveal significant gains and losses compared to those of isolated monomers, indicative of strong intermolecular donor-acceptor stabilizations in both directions. Although OB^−^∙∙∙BO^−^ achieves *C*_2_ symmetry, with each monomer serving equivalently as donor and acceptor to the other, it is interesting that NC^−^∙∙∙CN^−^ retains distinct asymmetry (Δ*Q*_CT_ ≈ 0.2e) between donor (left) and acceptor (right) monomers. This further illustrates what appears to be the fairly general role of *n*-π* interactions as the “smoking gun” responsible for numerous pseudo Jahn–Teller effects and related symmetry-breaking phenomena [79].

**Figure 5:**
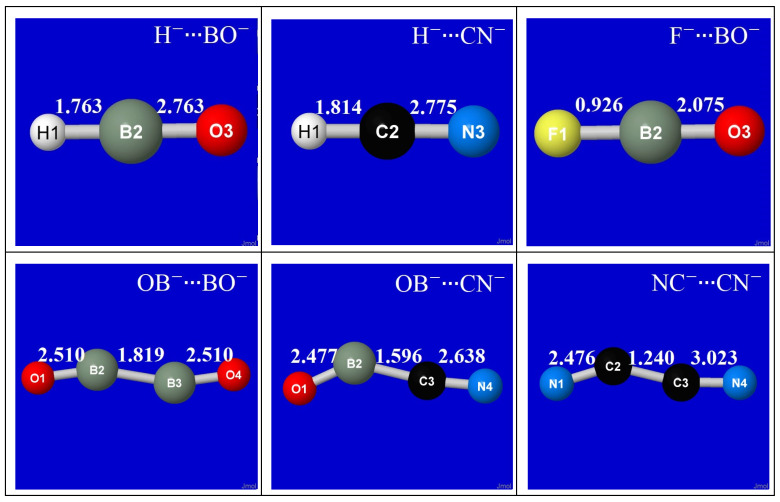
NRT bond orders for equilibrium species of Figure 1, showing robust single-molecule values (all *b*_ij_ > ½) for each species.

Figure 5 displays NRT bond orders for the equilibrium pi-hole species. The panels show that all these complexes achieve full molecular connectivity, i.e., newly formed connective linkages with bond orders in the general range (1 ≤ *b*_ij_ ≤ 2) of robust dative bonding or conjugated double-bonding. Compared to the bond orders of benzenoid species, the intermolecular bond orders of Figure 5 exhibit the high electrovalent (ionic) character that is characteristic of their “dative” origin. The calculated bond orders are generally expected to exhibit qualitative correlations with bond lengths, vibrational frequencies and other experimental properties. Although the data set is too small for meaningful statistical tests of such correlations, the strong (molecule-like) values of binding energies and vibrational frequencies quoted in Table 2 are evidently consistent with the robust intermolecular bond orders in Figure 5.

### 3.2. Unique Associations of Binding Properties with Specific Donor-Acceptor Interactions

The *uniqueness* of orbital-level donor-acceptor interactions as the origin of all such structural and energetic features of pi-hole attraction can also be demonstrated with standard “$DEL-deletion” options of the NBO program [80]. ^23^ $DEL-keylist input allows one to *delete* the specific *n*-π* interaction (Figure 2), or other partial or total contributions to intermolecular donor-acceptor “charge transfer” (CT), and recalculate the optimized potential energy curve as though the associated CT is *absent* in Nature. From the results for various deletions. one can then identify the unique “smoking gun” that acts as the specific *cause* for the appearance or disappearance of a specific *effect* of interest.

**Figure 6:**
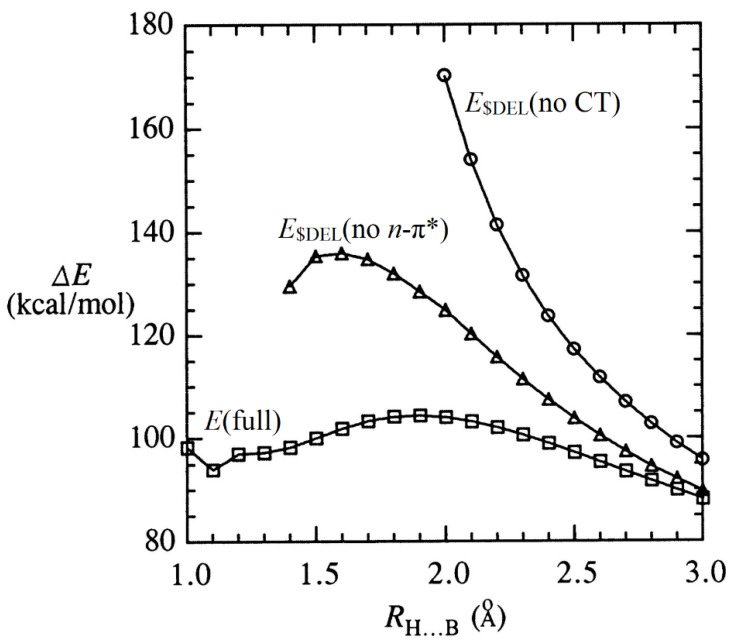
Relaxed-scan H^−^···BO^−^ interaction energy Δ*E* (relative to isolated H^−^ + BO^−^; cf. right-axis scale of upper-left panel in Figure 3), showing the strong deviations from the full calculation (squares) with respect to corresponding $DEL-type^23^ variational reoptimizations that “delete” either the single in-plane NBO *n*_H_-π*_BO_ matrix element (triangles) or *all* intermolecular NBO donor-acceptor interactions (circles).

As an illustrative example, we consider the H^−^∙∙∙BO^−^ complex (upper left panel of Figure 3), which exhibits apparent “entrance” pi-hole (*n*_H_-π*_BO_) and “terminal” σ-hole (*n*_H_-σ*_BO_) character of the deep binding well (16.45 kcal/mol). Figure 6 displays (on a greatly expanded energy scale) the intermolecular potential energy curves for (i) the original full calculation (squares; cf. Figure 3), (ii) the result of deleting the *single n*_H_-π*_BO_ interaction matrix element with the in-plane π*_BO_ NBO (triangles), and (iii) the result of deleting *all* intermolecular CT between the two units (circles). As shown in the figure, deletion of *n*_H_-π*_BO_ interaction sharply increases the repulsive character throughout the long-range “entry” region (and obliterates Bürgi–Dunitz geometry in favor of overall linear alignment), but eventually (near *R*_HB_ = 1.5 Å) enters the “σ-hole” binding region of strong *n*_H_-σ*_BO_ interaction. However, removal of *all* intermolecular CT is seen to lead uniformly (circles) to the steep steric *plus* electrostatic repulsions that would be “expected” in a naive classical-like view of intermolecular forces. All of these results are mutually consistent, both in the variational sense and in terms of the complementary picture of *n*-σ* and *n*-π* interactions as sketched above for the entire family of such anion–anion complexes. In effect, Bürgi–Dunitz approach geometry and metastable complex formation can be switched off (or on) by excluding (or including) the associated NBO donor-acceptor interactions from the variational calculation, even though such CT-type interactions make *no* contribution to electron density, electrostatic multipole moments, or other measurable properties of the monomers at large separation. The unique “driving force” for anion–anion attractions is thereby shown to have deep roots in the quantum mechanical domain of intermolecular CT interactions, rather than electrostatic properties of isolated monomers.

## 4. Discussion

Beyond the results discussed above for individual species, brief comments may be offered on broader electronic questions raised by the metastable binding wells. We address a few such questions in the following Q/A format:

*Why is F^−^ unable to form a pi-hole complex with CN^−^?* The on-axis lone pair of F^−^ appears to be obstructed from high overlap with the diatomic monomer by exchange-type steric repulsions with its inner (1s)^2^ core, whereas H^−^ presents only the weaker Coulomb-type repulsion to nuclear charge. In addition, the off-axis lone pairs of F^−^ lead to steric congestion in non-linear geometry and are relatively ineffective in donating to the vacant π*_CN_ orbital in linear geometry, because such formal 2e donation (to yield “CN^3−^,” isoelectronic to O_2_) demands triplet open-shell character.

*Why are the MP2 binding energies so different from DFT values?* Dynamic electron correlation (primarily, dynamic left-right bond polarization, better described at MP2 than DFT level) is apparently a critical factor in stabilizing these highly anionic complexes.

*Why is open-shell description required at some points on the potential curves?* Singlet-diradical character is an effective means to describe dynamical electron correlation effects in single-determinant DFT methods. Such open-shell intrusions (with typical maximum 〈S^2^〉 ≈ 0.5 value) occur near the most distressed regions of the anion–anion potential curves, particularly the transition state region of *n*-π* approach and (where applicable) the ensuing short-range realignment to *n*-σ* linear geometry. Additional details of the partial diradicaloid geometries and spin values of each species are included in Supporting Information (SI).

**Figure 7:**
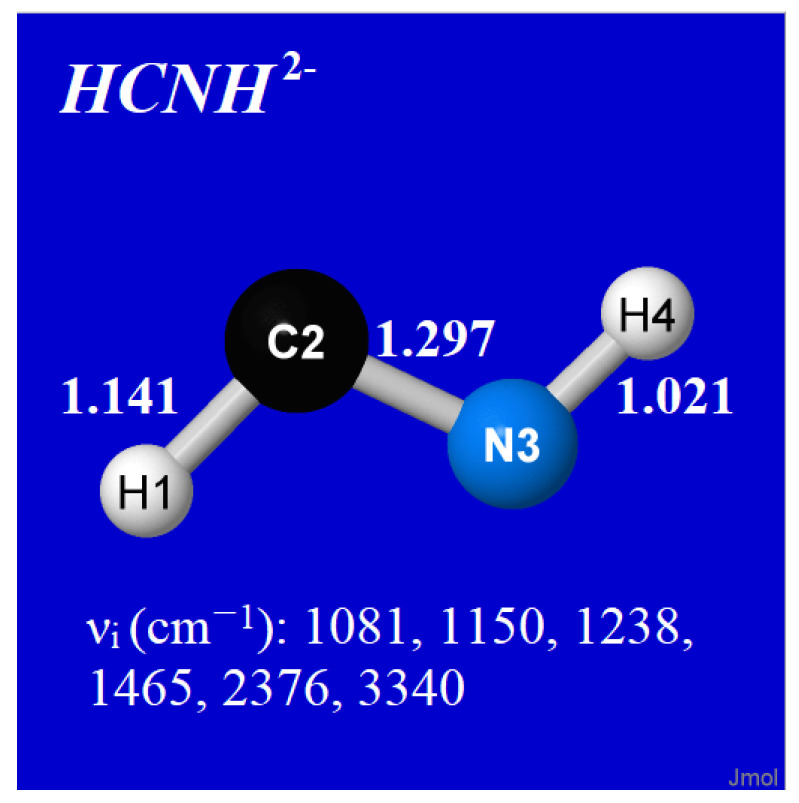
Optimized structure of model HCN^2−^∙∙∙H complex, showing bond lengths (Å) and harmonic vibration frequencies (cm^−1^) of the equilibrium B3LYP/6-311++G** species.

*What about possible spontaneous decay of these species via electronic autodetachment?* Proper description of the lifetimes of such metastable species, whether for shape potentials (Figure 3) or Feshbach-type autoionization phenomena, requires more advanced theoretical methods [81]. However, as a simple DFT-level model of possible electronic autodetachment, we considered HCN^2−^ in the presence of an electrophile (here taken as a neutral H-atom, initially 2 Å from C in T-shaped approach) that might be expected to capture an electron from the dianion and autodissociate to the asymptotic low-energy limit. However, rather than capturing an electron from the dianion, the H-atom is instead found to be captured *by* the dianion, optimizing to a surprising HCNH^2−^ dianion radical (trans-bent “diimine-like” species of *C*_s_ symmetry, electronically and vibrationally stable) as shown in Figure 7. The model thereby suggests *absence* of any low-barrier channel to separated monoanions, corresponding to appreciable HCN^2−^ lifetime with respect to predissociation or autodetachment decay modes.

*Doesn’t the Hellmann–Feynman theorem guarantee that all such interactions can be equivalently described as “electrostatic” in nature?* The Hellmann–Feynman theorem [82,83] is a mixed bag. It is undoubtedly a true statement if written in terms of the exact wavefunction. However, it was recognized by early workers [84] that the Hellmann–Feynman theorem gives large, uncontrollable errors if expressed in terms of approximate wavefunctions or densities (particularly those with inexact satisfaction of nuclear and electronic cusp conditions [85]). At face value, the Hellmann–Feynman theorem allows interpretation of covalent chemical bonding and all other intra- and intermolecular quantum chemical phenomena as “electrostatically driven.” However, as concluded in Ref. [84], “A superficial application of the theorem to systems described by approximate wave functions, even through they may seem reasonable from a variational point of view, can lead to totally absurd results.” Unlike other quantum chemistry theorems in common usage, the Hellmann–Feynman theorem lacks an equivalent variational formulation or valid perturbation expansion (of non-zero convergence radius) to provide the stability or bounding properties necessary for controlling the errors arising from approximate wavefunctions or densities. Whether Hellmann–Feynman-based numerical evaluations have useful interpretive value (e.g., in predictive correlations with measurable properties of H-bonded systems [86] requires case-by-case demonstration rather than untested acceptance as irrefutable fact.

In this context, it should also be stressed that the discussed controversies between orbital-level vs. classical-type conceptions of intermolecular bonding refer not to the esoterics of Hellmann–Feynman theory, but rather to superficial “dipole–dipole” rationalizations of hydrogen bonding (and related halogen, pnicogen, … bonding phenomena) that still pervade many freshman-level expositions [12]. Similar controversies surround the quasi-classical electrostatic multipole logic that underlies symmetry adapted perturbation theory (SAPT) [87,88] and empirical variants of molecular dynamics (MD) simulation methods [89,90]. The present results provide additional cases that fail the “smell test” of plausible interpretation or numerical fitting with classical-type electrostatic constructs, but are in full harmony with the broader picture of orbital-level donor-acceptor interactions.

## 5. Concluding Summary

The present work illustrates how “covalency conquers Coulombics” in still another class of intermolecular interaction phenomena. The results build on the conceptual insights provided by anti-electrostatic H-bonds [34] and related *n*-σ* species [33], but reveal also the surprising interplay between higher-order *n*-σ*/*n*-π* *couplings* that become available in unsaturated supramolecular complexes. The orbital logic of *n*-π* interactions can also be recognized in a variety of intramolecular phenomena [1,2,3,4,5,6,7,8], including the characteristic stereoelectronic propensities of alicyclic ring-closure reactions as summarized in Baldwin’s rules [91].

Our results do not discount the anisotropies of atomic charge distribution underlying calculated [17,18,19,20,21,22,23,24,25,26,27,28,29] or measured [92] variations of the associated electrostatic potential around isolated monomers. However, they demonstrate that such (expected!) anisotropies provide at most secondary modulating influence on the primary *n*-π* CT-type orbital interaction that is here shown to be the unique “cause” for the characteristic Bürgi–Dunitz approach geometry and deep binding well “effect” of complex formation.

The elementary atomic and diatomic anions of the present study were chosen rather arbitrarily as candidate gas-phase complexes that are “too small to hide” the fierce Coulombic opposition to chemical bonding interactions through dielectric shielding or charge dispersal effects. Such complexes underscore the fundamental conceptual dichotomy between classical electrostatic vs. quantum covalency rationalizations of supramolecular binding in sharpest terms. We expect that many additional examples of chemically and biochemically relevant like-charge bonding remain to be discovered in liquid and solid phases, but the present need is for detection of metastable like-charge species in isolated gas-phase conditions as a more stringent experimental test of the computational predictions.

## Figures and Tables

**Table 1:** Optimized geometrical parameters [interatomic distances *R*_ij_ (Å), angles *A*_ijk_ (°)] for equilibrium species of Figure 1.

Species	*R*_12_	*R*_23_	*R*_34_	*A*_123_	*A*_234_
H^−^∙∙∙BO^−^	1.130	1.217	-	180.0	-
H^−^∙∙∙CN^−^	1.061	1.171	-	180.0	-
F^−^∙∙∙BO^−^	1.303	1.219	-	180.0	-
OB^−^∙∙∙BO^−^	1.240	1.631	1.240	162.9	162.9
OB^−^∙∙∙CN^−^	1.257	1.515	1.172	141.9	171.3
NC^−^∙∙∙CN^−^	1.206	1.427	1.193	149.5	166.7

**Table 2:** Calculated equilibrium well depth (Δ*E*_bind_, kcal/mol), ionic dissociation energy (Δ*E*_dissoc_, kcal/mol), harmonic vibrational frequencies (ν_i_^(eq)^, cm^−1^), and transition-state imaginary frequency (ν_1_^‡^, cm^−1^) for species of Figure 1, comparing DFT (left) and MP2 (right) theoretical levels.

Species	Property	DFT	MP2
H^−^···BO^−^	Δ*E*_bind_	−16.45	−24.96
	[−Δ*E*_dissoc_]	[+87.72]	[+81.24]
	ν_i_^(eq)^	756,825,1771,2974	827,835,1771,3020
	ν_1_^‡^	837i	869i
H^−^···CN^−^	Δ*E*_bind_	−26.80	−37.24
	[−Δ*E*_dissoc_]	[+95.46]	[+89.94]
	ν_i_^(eq)^	923(2),2181,3531	900(2),2263,3463
	ν_1_^‡^	1373i	1517i
F^−^···BO^−^	Δ*E*_bind_	−11.01	−15.37
	[−Δ*E*_dissoc_]	[+88.26]	[+85.94]
	ν_i_^(eq)^	362(2),891,1970	428(2),896,1967
	ν_1_^‡^	258i	301i
F^−^···CN^−^	Δ*E*_bind_	*NA*	*NA*
OB^−^···BO^−^	Δ*E*_bind_	−6.50	−3.55
	[−Δ*E*_dissoc_]	[+91.64]	[+97.72]
	ν_i_^(eq)^	211,227,345, 601,1656,1831	207,245,438, 572,1591,1829
	ν_1_^‡^	299i	321i
OB^−^···CN^−^	Δ*E*_bind_	−8.78	−10.40
	[−Δ*E*_dissoc_]	[+100.97]	[+107.42]
	ν_i_^(eq)^	195,339,487, 687,1653,2107	237,355,556, 680,1563,2775
	ν_1_^‡^	436i	549i
NC^−^···CN^−^	Δ*E*_bind_	−7.44	−12.71
	[−Δ*E*_dissoc_]	[+145.13]	[+151.23]
	ν_i_^(eq)^	148,230,287, 881,1737,2161	248,489,528, 896,1909,2597
	ν_1_^‡^	979i	1166i

## Data Availability

Not applicable.

## Supplementary Materials

The following supporting information can be downloaded online. The Supporting Information (SI) file contains optimized geometrical coordinates, NBO keyword input, and other computational details in ready-to-run Gaussian input files for all equilibrium and transition state species described in the paper.

## Sample Availability

Samples of the compounds not are available from the authors.
