# Supplementary material for: Anti-Electrostatic Pi-Hole Bonding: How Covalency Conquers Coulombics
Source: Molecules. 2022 Jan 7;27(2):377. doi: 10.3390/molecules27020377 (PMC8780338; doi:10.3390/molecules27020377)
Supplement: Supplementary file 1 [file molecules-27-00377-s001.zip › molecules-1528857-supplementary.pdf]

Supporting Information for
"Anti-Electrostatic Pi-Hole Bonding: How Covalency Conquers Coulombics"

Frank Weinhold*
Department of Chemistry, University of Wisconsin-Madison, Madison WI, USA
(*weinhold@chem.wisc.edu*)

This *Supporting Information* includes Gaussian input files for each of the six binary complexes discussed in the main text, summarizing optimized geometries, frequencies, and S^2 values. For each complex, separate listings are given for

- "`X(-)...Y(-) Input Files`", for stationary equilibrium and transition state features at B3LYP/6-311++G** DFT level;
- "`X(-)...Y(-) IRC Input Files`", for intrinsic reaction coordinate (IRC) features at DFT level;
- "`X(-)...Y(-) MP2 Input Files`", for MP2/6-311++G** stationary features; and
- "`X(-)...Y(-) Segment Scan Input Files`", for various relaxed-scan (opt=modredundant or opt=z-matrix) segments of plotted DFT potential curves.

The title section of each input file often summarizes the important energy, geometry, and spin results that are generated by the file. Each file commonly includes also the $NBO keylist input that generates NBO/NRT descriptors and orbital graphics used throughout the main text.

```
***************************** H(-)...BO(-) Input Files ***************************** %mem=2gb
%nprocshared=8
%chk=h_m_bo_m_eq
#ub3lyp/6-311++G** pop=nbo7read guess=mix stable=opt
  antielectrostatic pi-hole: H(-)...BO(-), E(UB3LYP) =  -
100.535380549
-2 1
H
B   1   1.2924
O   2   1.2519   1   132.65

$nbo file=h_m_bo_m archive $end

--Link1--
%mem=2gb
%nprocshared=8
%chk=h_m_bo_m_eq
#ub3lyp/6-311++G** pop=nbo7read guess=read

 AE pi-hole: H(-)...BO(-), E(UB3LYP) =  -100.548481569(-16.45)[87.72], <S**2>= 0.9543
nu: 756,825,1771,2974
 Vibrational temperatures:   1087.52  1186.59  2547.60  4278.78
           (Kelvin)
 Zero-point correction=                           0.014410 (Hartree/Particle)
 Thermal correction to Energy=                    0.016936
```

```
 Thermal correction to Enthalpy=                  0.017880
Thermal correction to Gibbs Free Energy=        -0.005120  Sum of
electronic and zero-point Energies=           -100.534072
 Sum of electronic and thermal Energies=              -100.531546
 Sum of electronic and thermal Enthalpies=            -100.530602
 Sum of electronic and thermal Free Energies=         -100.553601

-2 1
H
B   1   1.1259
x   2   1.      1   90.
O   2   1.2089  3   90.   1  180.00

$nbo file=h_m_bo_m_eq archive $end

--Link1--
--Link1--
%mem=2gb
%nprocshared=8
%chk=h_m_bo_m_ts
#b3lyp/6-311++G** pop=nbo7read
  antielectrostatic pi-hole: H(-)...BO(-) t.s., E(RB3LYP) =  -100.522263816(e-
stable)  nu: -837,542,1594
 Vibrational temperatures:    780.00  2292.70
          (Kelvin)
 Zero-point correction=                           0.004865 (Hartree/Particle)
 Thermal correction to Energy=                    0.007896
 Thermal correction to Enthalpy=                  0.008840
Thermal correction to Gibbs Free Energy=        -0.016883
 Sum of electronic and zero-point Energies=           -100.517398
 Sum of electronic and thermal Energies=              -100.514368
 Sum of electronic and thermal Enthalpies=            -100.513424
 Sum of electronic and thermal Free Energies=         -100.539147

-2 1
H
B   1   1.8867
O   2   1.2537   1   116.30

$nbo file=h_m_bo_m_ts archive $end

--Link1--
%mem=2gb
%nprocshared=8
%chk=h_m_bo_m_eq
#b3lyp/6-311++G** pop=nbo7read

 antielectrostatic pi-hole: H(-)...BO(-), E(RB3LYP) =  -100.533580545 [-
7.10 kcal/mol w.r.t. t.s.; +97.07 w.r.t. H(-) + BO(-), -100.6882742]  nu:
652,1513,1832
 Vibrational temperatures:    938.40  2177.11  2635.35
          (Kelvin)
 Zero-point correction=                           0.009106 (Hartree/Particle)
 Thermal correction to Energy=                    0.012078
Thermal correction to Enthalpy=                  0.013022  Thermal
correction to Gibbs Free Energy=        -0.012008
 Sum of electronic and zero-point Energies=           -100.524475
 Sum of electronic and thermal Energies=              -100.521503
 Sum of electronic and thermal Enthalpies=            -100.520559
Sum of electronic and thermal Free Energies=         -100.545589
-2 1
H
B   1   1.2924
O   2   1.2519   1   132.65

$nbo file=h_m_bo_m_eq archive nrt plot $end
```

```
--Link1--
**************************** H(-)...BO(-) IRC Input Files **************************** %mem=2gb
%nprocshared=8
%chk=h_m_bo_m_irc
#b3lyp/6-311++G** pop=nbo7read

 AEPH: H(-)...BO(-) pt.#m12, E = -100.533504121, IRC=-1.26406

-2 1
     1    -0.830855    -0.000000    -1.596268
     5    -0.610246     0.000000    -0.315553
     8     0.472384     0.000000     0.317774

$nbo file=h_m_bo_m_m12 archive nrt plot $end

--Link1--
%mem=2gb
%nprocshared=8
%chk=h_m_bo_m_irc
#b3lyp/6-311++G** pop=nbo7read

 AEPH: H(-)...BO(-) pt.#m11, E = -100.533232846 , IRC=-1.15857

-2 1
     1    -0.785225     0.000000    -1.608088
     5    -0.616433     0.000000    -0.310487
     8     0.473768    -0.000000     0.315033

$nbo file=h_m_bo_m_m11 archive nrt plot $end

--Link1--
%mem=2gb
%nprocshared=8
%chk=h_m_bo_m_irc
#b3lyp/6-311++G** pop=nbo7read

 AEPH: H(-)...BO(-) pt.#m10, E = -100.532729238, IRC=-1.05435

-2 1
     1    -0.744816     0.000000    -1.632273
     5    -0.622422    -0.000000    -0.304832
     8     0.475344     0.000000     0.312664

$nbo file=h_m_bo_m_m10 archive nrt plot $end

--Link1--
%mem=2gb
%nprocshared=8
%chk=h_m_bo_m_irc
#b3lyp/6-311++G** pop=nbo7read

 AEPH: H(-)...BO(-) pt.#m9, E = -100.531864061, IRC=-0.94960

-2 1
     1    -0.719164     0.000000    -1.674318
     5    -0.626673     0.000000    -0.298779
     8     0.476653    -0.000000     0.311147

$nbo file=h_m_bo_m_m9 archive nrt plot $end

--Link1--
%mem=2gb
%nprocshared=8
%chk=h_m_bo_m_irc
#b3lyp/6-311++G** pop=nbo7read
```

```
 AEPH: H(-)...BO(-) pt.#m8, E = -100.530602524, IRC=-0.84420

-2 1
     1    -0.702860     0.000000    -1.722574
     5    -0.629580     0.000000    -0.292911
     8     0.477627    -0.000000     0.310148

$nbo file=h_m_bo_m_m8 archive nrt plot $end

--Link1--
%mem=2gb
%nprocshared=8
%chk=h_m_bo_m_irc
#b3lyp/6-311++G** pop=nbo7read

 AEPH: H(-)...BO(-) pt.#m7, E = -100.529095766, IRC=-0.73870

-2 1
     1    -0.690945     0.000000    -1.772825
     5    -0.631801     0.000000    -0.287212
     8     0.478404    -0.000000     0.309392

$nbo file=h_m_bo_m_m7 archive nrt plot $end

--Link1--
%mem=2gb
%nprocshared=8
%chk=h_m_bo_m_irc
#b3lyp/6-311++G** pop=nbo7read

 AEPH: H(-)...BO(-) pt.#m6, E = -100.527520817, IRC=-0.63318

-2 1
     1    -0.681340     0.000000    -1.823924
     5    -0.633645    -0.000000    -0.281627
     8     0.479069    -0.000000     0.308768

$nbo file=h_m_bo_m_m6 archive nrt plot $end

--Link1--
%mem=2gb
%nprocshared=8
%chk=h_m_bo_m_irc
#b3lyp/6-311++G** pop=nbo7read

 AEPH: H(-)...BO(-) pt.#m5, E = -100.526022924, IRC=-0.52765

-2 1
     1    -0.673134     0.000000    -1.875473
     5    -0.635257    -0.000000    -0.276120
     8     0.479661    -0.000000     0.308226

$nbo file=h_m_bo_m_m5 archive nrt plot $end

--Link1--
%mem=2gb
%nprocshared=8
%chk=h_m_bo_m_irc
#b3lyp/6-311++G** pop=nbo7read

 AEPH: H(-)...BO(-) pt.#m4, E = -100.524708128, IRC=-0.42212

-2 1
     1    -0.665840     0.000000    -1.927292
     5    -0.636716    -0.000000    -0.270672
     8     0.480206     0.000000     0.307741
```

```
$nbo file=h_m_bo_m_m4 archive nrt plot $end

--Link1--
%mem=2gb
%nprocshared=8
%chk=h_m_bo_m_irc
#b3lyp/6-311++G** pop=nbo7read

 AEPH: H(-)...BO(-) pt.#m3, E = -100.523646068, IRC=-0.31660

-2 1
     1    -0.659187     0.000000    -1.979292
     5    -0.638071    -0.000000    -0.265271
     8     0.480719    -0.000000     0.307300

$nbo file=h_m_bo_m_m3 archive nrt plot $end

--Link1--
%mem=2gb
%nprocshared=8
%chk=h_m_bo_m_irc
#b3lyp/6-311++G** pop=nbo7read

 AEPH: H(-)...BO(-) pt.#m2, E = -100.522876621, IRC=-0.21107

-2 1
     1    -0.653004     0.000000    -2.031421
     5    -0.639352    -0.000000    -0.259909
     8     0.481211     0.000000     0.306894

$nbo file=h_m_bo_m_m2 archive nrt plot $end

--Link1--
%mem=2gb
%nprocshared=8
%chk=h_m_bo_m_irc
#b3lyp/6-311++G** pop=nbo7read

 AEPH: H(-)...BO(-) pt.#m1, E = -100.522416270, IRC=-0.10555

-2 1
     1    -0.647136     0.000000    -2.083636
     5    -0.640589     0.000000    -0.254581
     8     0.481693     0.000000     0.306516

$nbo file=h_m_bo_m_m1 archive nrt plot $end

--Link1--
%mem=2gb
%nprocshared=8
%chk=h_m_bo_m_ts
#b3lyp/6-311++G** pop=nbo7read

 AEPH: H(-)...BO(-) t.s., E(RB3LYP) =  -100.522263816, IRC=0.0000

-2 1
H
B   1   1.8867
O   2   1.2537   1   116.30

$nbo file=h_m_bo_m_ts archive nrt plot $end

--Link1--
%mem=2gb
%nprocshared=8
```

```
%chk=h_m_bo_m_irc
#b3lyp/6-311++G** pop=nbo7read

 AEPH: H(-)...BO(-) pt.#p1, E = -100.522405723, IRC=0.10555

-2 1
     1    -0.636389     0.000000    -2.188361
     5    -0.642936     0.000000    -0.244016
     8     0.482632     0.000000     0.305843

$nbo file=h_m_bo_m_p1 archive nrt plot $end

--Link1--
%mem=2gb
%nprocshared=8
%chk=h_m_bo_m_irc
#b3lyp/6-311++G** pop=nbo7read

 AEPH: H(-)...BO(-) pt.#p2, E =  -100.522820106, IRC=0.21107

-2 1
     1    -0.631448     0.000000    -2.240819
     5    -0.644059    -0.000000    -0.238785
     8     0.483093     0.000000     0.305548

$nbo file=h_m_bo_m_p2 archive nrt plot $end

--Link1--
%mem=2gb
%nprocshared=8
%chk=h_m_bo_m_irc
#b3lyp/6-311++G** pop=nbo7read

 AEPH: H(-)...BO(-) pt.#p3, E = -100.523480053, IRC=0.31660

-2 1
     1    -0.626635     0.000000    -2.293321
     5    -0.645179     0.000000    -0.233579
     8     0.483561    -0.000000     0.305273

$nbo file=h_m_bo_m_p3 archive nrt plot $end

--Link1--
%mem=2gb
%nprocshared=8
%chk=h_m_bo_m_irc
#b3lyp/6-311++G** pop=nbo7read

 AEPH: H(-)...BO(-) pt.#p4, E = -100.524356312, IRC=0.42213

-2 1
     1    -0.621992     0.000000    -2.345873
     5    -0.646290    -0.000000    -0.228400
     8     0.484033     0.000000     0.305019

$nbo file=h_m_bo_m_p4 archive nrt plot $end

--Link1--
%mem=2gb
%nprocshared=8
%chk=h_m_bo_m_irc
#b3lyp/6-311++G** pop=nbo7read

 AEPH: H(-)...BO(-) pt.#p5, E = -100.525419041, IRC=0.52766

-2 1
```

```
     1    -0.617512     0.000000    -2.398470
     5    -0.647393    -0.000000    -0.223247
     8     0.484510    -0.000000     0.304787

$nbo file=h_m_bo_m_p5 archive nrt plot $end

--Link1--
%mem=2gb
%nprocshared=8
%chk=h_m_bo_m_irc
#b3lyp/6-311++G** pop=nbo7read

 AEPH: H(-)...BO(-) pt.#p6, E = -100.526639119, IRC=0.63319

-2 1
     1    -0.613186     0.000000    -2.451112
     5    -0.648490    -0.000000    -0.218122
     8     0.484992    -0.000000     0.304576

$nbo file=h_m_bo_m_p6 archive nrt plot $end

--Link1--
%mem=2gb
%nprocshared=8
%chk=h_m_bo_m_irc
#b3lyp/6-311++G** pop=nbo7read

 AEPH: H(-)...BO(-) pt.#p7, E = -100.527989098, IRC=0.73872

-2 1
     1    -0.609010    -0.000000    -2.503796
     5    -0.649582    -0.000000    -0.213023
     8     0.485481     0.000000     0.304386

$nbo file=h_m_bo_m_p7 archive nrt plot $end

--Link1--
%mem=2gb
%nprocshared=8
%chk=h_m_bo_m_irc
#b3lyp/6-311++G** pop=nbo7read

 AEPH: H(-)...BO(-) pt.#p8, E = -100.529443732, IRC=0.84425

-2 1
     1    -0.604976    -0.000000    -2.556521
     5    -0.650670    -0.000000    -0.207951
     8     0.485975    -0.000000     0.304217

$nbo file=h_m_bo_m_p8 archive nrt plot $end

--Link1--
%mem=2gb
%nprocshared=8
%chk=h_m_bo_m_irc
#b3lyp/6-311++G** pop=nbo7read

 AEPH: H(-)...BO(-) pt.#p9, E = -100.530980226, IRC=0.94978

-2 1
     1    -0.601081    -0.000000    -2.609286
     5    -0.651754     0.000000    -0.202906
     8     0.486476    -0.000000     0.304069

$nbo file=h_m_bo_m_p9 archive nrt plot $end
```

```
--Link1--
%mem=2gb
%nprocshared=8
%chk=h_m_bo_m_irc
#b3lyp/6-311++G** pop=nbo7read

 AEPH: H(-)...BO(-) pt.#p10, E = -100.532578427, IRC=1.05532

-2 1
     1    -0.597320    -0.000000    -2.662089
     5    -0.652835    -0.000000    -0.197887
     8     0.486983    -0.000000     0.303942

$nbo file=h_m_bo_m_p10 archive nrt plot $end

--Link1--
%mem=2gb
%nprocshared=8
%chk=h_m_bo_m_irc
#b3lyp/6-311++G** pop=nbo7read

 AEPH: H(-)...BO(-) pt.#p11, E = -100.534220845, IRC=1.16085

-2 1
     1    -0.593690    -0.000000    -2.714929
     5     -0.653910     -0.000000     -0.192895
8      0.487495      0.000000      0.303835 $nbo
file=h_m_bo_m_p11 archive nrt plot $end

--Link1--
%mem=2gb
%nprocshared=8
%chk=h_m_bo_m_irc
#b3lyp/6-311++G** pop=nbo7read

 AEPH: H(-)...BO(-) pt.#p12, E = -100.535892486, IRC=1.26638

-2 1
     1    -0.590190     0.000000    -2.767806
     5    -0.654981    -0.000000    -0.187929
     8     0.488011    -0.000000     0.303748

$nbo file=h_m_bo_m_p12 archive nrt plot $end

--Link1--
%mem=2gb
%nprocshared=8
%chk=h_m_bo_m_irc
#b3lyp/6-311++G** pop=nbo7read

 AEPH: H(-)...BO(-) pt.#p13, E = -100.537580678, IRC=1.37192

-2 1
     1    -0.586818    -0.000000    -2.820720
     5    -0.656047    -0.000000    -0.182989
     8     0.488532     0.000000     0.303682

$nbo file=h_m_bo_m_p13 archive nrt plot $end

--Link1--
%mem=2gb
%nprocshared=8
%chk=h_m_bo_m_irc
#b3lyp/6-311++G** pop=nbo7read

 AEPH: H(-)...BO(-) pt.#p14, E = -100.539274927, IRC=1.47745
```

```
-2 1
     1    -0.583572    -0.000000    -2.873668
     5    -0.657105    -0.000000    -0.178074
     8     0.489056    -0.000000     0.303636

$nbo file=h_m_bo_m_p14 archive nrt plot $end

--Link1--
%mem=2gb
%nprocshared=8
%chk=h_m_bo_m_irc
#b3lyp/6-311++G** pop=nbo7read

 AEPH: H(-)...BO(-) pt.#p15, E = -100.540966680, IRC=1.58298

-2 1
     1    -0.580451    -0.000000    -2.926651
     5    -0.658157    -0.000000    -0.173185
     8     0.489583    -0.000000     0.303609

$nbo file=h_m_bo_m_p15 archive nrt plot $end

--Link1--
%mem=2gb
%nprocshared=8
%chk=h_m_bo_m_irc
#b3lyp/6-311++G** pop=nbo7read

 AEPH: H(-)...BO(-) pt.#p16, E = -100.542649051, IRC=1.68852

-2 1
     1    -0.577453    -0.000000    -2.979669
     5    -0.659200    -0.000000    -0.168321
     8     0.490112     0.000000     0.303602

$nbo file=h_m_bo_m_p16 archive nrt plot $end

--Link1--
%mem=2gb
%nprocshared=8
%chk=h_m_bo_m_irc
#b3lyp/6-311++G** pop=nbo7read

 AEPH: H(-)...BO(-) pt.#p17, E = -100.544316593, IRC=1.79405

-2 1
     1    -0.574576    -0.000000    -3.032720
     5    -0.660234    -0.000000    -0.163482
     8     0.490643    -0.000000     0.303614

$nbo file=h_m_bo_m_p17 archive nrt plot $end

--Link1--
%mem=2gb
%nprocshared=8
%chk=h_m_bo_m_irc
#b3lyp/6-311++G** pop=nbo7read

 AEPH: H(-)...BO(-) pt.#p18, E = -100.545965108, IRC=1.89959

-2 1
     1    -0.571819     0.000000    -3.085803
     5    -0.661257    -0.000000    -0.158668
     8     0.491173    -0.000000     0.303645
```

```
$nbo file=h_m_bo_m_p18 archive nrt plot $end

--Link1--
%mem=2gb
%nprocshared=8
%chk=h_m_bo_m_irc
#b3lyp/6-311++G** pop=nbo7read

 AEPH: H(-)...BO(-) pt.#p19, E = -100.547591436, IRC=2.00512

-2 1
     1    -0.569178     0.000000    -3.138919
     5    -0.662270     0.000000    -0.153879
     8     0.491704    -0.000000     0.303695

$nbo file=h_m_bo_m_p19 archive nrt plot $end

--Link1--
%mem=2gb
%nprocshared=8
%chk=h_m_bo_m_irc
#b3lyp/6-311++G** pop=nbo7read

 AEPH: H(-)...BO(-) pt.#p20, E = -100.549193294, IRC=2.11065

-2 1
     1    -0.566653     0.000000    -3.192067
     5    -0.663271    -0.000000    -0.149113
     8     0.492234    -0.000000     0.303764

$nbo file=h_m_bo_m_p20 archive nrt plot $end

--Link1--
--Link1--
***************************** H(-)...BO(-) MP2 Input Files ***************************** %mem=2gb
%nprocshared=8
%chk=h_m_bo_m_eq_mp2
#uhf/6-311++G** guess=mix stable=opt

 AE pi-hole: H(-)...BO(-),

-2 1
H
B   1   1.2924
O   2   1.2519   1   132.65

--Link1--
%mem=2gb
%nprocshared=8
%chk=h_m_bo_m_eq_mp2
#mp2/6-311++G** pop=nbo7read guess=read density=current

 AE pi-hole: H(-)...BO(-), EUMP2 = -0.10026293196947D+03(-24.96),(S**2,1)=  0.99415
nu: 827,835,1771,3020
 Vibrational temperatures:   1189.68  1201.58  2547.39  4344.48
          (Kelvin)
 Zero-point correction=                           0.014699 (Hartree/Particle)
 Thermal correction to Energy=                    0.017201
Thermal correction to Enthalpy=                  0.018145  Thermal
correction to Gibbs Free Energy=        -0.004833
 Sum of electronic and zero-point Energies=           -100.248233
 Sum of electronic and thermal Energies=              -100.245731
 Sum of electronic and thermal Enthalpies=            -100.244787
 Sum of electronic and thermal Free Energies=         -100.267765

-2 1
```

```
                1                   0.000000    0.000000   -1.744171
                5                   0.000000    0.000000   -0.614470
8                   0.000000    0.000000    0.602065

$nbo file=h_m_bo_m_eq archive fixdm $end

--Link1--
%mem=2gb
%nprocshared=8
%chk=h_m_bo_m_ts_mp2
#uhf/6-311++G** stable=opt

 AE pi-hole: H(-)...BO(-) t.s. guess,

-2 1
H
B   1   1.8867
O   2   1.2537   1   116.30

--Link1--
%mem=2gb
%nprocshared=8
%chk=h_m_bo_m_ts_mp2
#mp2/6-311++G** pop=nbo7read opt=(ts,calcall) density=current

 AE pi-hole: H(-)...BO(-) t.s., EUMP2 = -0.10022314981872D+03
nu: -869,555,1569
 Vibrational temperatures:    798.99  2256.88
          (Kelvin)

 Zero-point correction=                           0.004839 (Hartree/Particle)
 Thermal correction to Energy=                    0.007861
 Thermal correction to Enthalpy=                  0.008805
Thermal correction to Gibbs Free Energy=        -0.016922
 Sum of electronic and zero-point Energies=          -100.218311
 Sum of electronic and thermal Energies=             -100.215289
 Sum of electronic and thermal Enthalpies=           -100.214344
Sum of electronic and thermal Free Energies=         -100.240071

-2 1
                1                  -1.580255    1.466763   -0.000000
                5                   0.121558    0.664782   -0.000000
8                   0.121558   -0.598834    0.000000

$nbo file=h_m_bo_m_ts_mp2 archive fixdm $end

--Link1--
**************************** H(-)...BO(-) Segment Scan Input Files ****************************
%mem=2gb
%nprocshared=8
%chk=h_m_bo_m_seg
#ub3lyp/6-311++G** pop=nbo7read guess=mix stable=opt
 AE pi-hole: H(-)...BO(-), E(UB3LYP) =  -100.535380549

-2 1
H
B   1   1.8867 x   2
1.      1   90.
O   2   1.2537  3   26.30  1  180.

$nbo file=h_m_bo_m archive $end

--Link1--
%mem=2gb
%nprocshared=8
%chk=h_m_bo_m_seg
#ub3lyp/6-311++G** pop=nbo7read guess=read scf=qc opt=modredundant
```

```
 AE pi-hole: H(-)...BO(-), E(UB3LYP) =  -100.548481569(-16.45), <S**2>= 0.9543
nu: 756,825,1771,2974
 Vibrational temperatures:   1087.52  1186.59  2547.60  4278.78
          (Kelvin)
 Zero-point correction=                           0.014410 (Hartree/Particle)
 Thermal correction to Energy=                    0.016936
 Thermal correction to Enthalpy=                  0.017880
Thermal correction to Gibbs Free Energy=        -0.005120
 Sum of electronic and zero-point Energies=           -100.534072
 Sum of electronic and thermal Energies=              -100.531546
 Sum of electronic and thermal Enthalpies=            -100.530602
Sum of electronic and thermal Free Energies=          -100.553601
```

point	R(HB)	R(BO)	A(HBO)	E	dE	S^2
m9	0.6759	1.1994	180.00	-100.265791588		0.0000
m8	0.7259	1.2012	180.00	-100.351917389		0.0472
m7	0.7759	1.2033	180.00	-100.415402318		0.2906
m6	0.8259	1.2047	180.00	-100.461610206		0.4802
m5	0.8759	1.2058	180.00	-100.494605572	+17.36	0.6307
m4	0.9259	1.2066	180.00	-100.517525004	+2.97	0.7467
m3	0.9759	1.2073	180.00	-100.532768225	-6.59	0.8310
m2	1.0259	1.2078	180.00	-100.542151248	-12.48	0.8894
m1	1.0759	1.2083	180.00	-100.547042553	-15.55	0.9286
eq*	1.1259	1.2089	180.00	-100.548481561	-16.45	0.9543
p1	1.1759	1.2089	180.00	-100.547260728	-15.69	0.9707
p2	1.2259	1.2089	180.00	-100.543997855	-13.64	0.9815
p3	1.2759	1.2093	180.00	-100.539175720	-10.61	0.9885
p4	1.3259	1.2095	180.00	-100.533172681	-6.85	0.9928
p5	1.3759	1.2202	180.00	-100.520969823	0.81	1.0003
p6	1.4259	1.2213	180.00	-100.514217060	5.05	1.0003
p7	1.4759	1.2213	180.00	-100.506853147	9.67	1.0003
p8	1.5259	1.2214	180.00	-100.499057454	14.56	1.0003
p9	1.5759	1.2214	180.00	-100.490977286	19.63	1.0003
p10	1.6259	1.2215	180.00	-100.482731607	24.81	1.0003
p11	1.6759	1.2215	180.00	-100.474416360	30.02	1.0003
p12	1.7259	1.2216	180.00	-100.466108269	35.24	1.0003
p12a	1.7359	1.2216	180.00	-100.464455965	36.27	1.0003
p12b	1.7459	1.2216	180.00	-100.462804071	37.31	1.0003
p12c	1.7559	1.2253	180.00	-100.500379373	13.73	0.0000
p12d	1.7659	1.2549	118.74	-100.522968746	-0.44	0.0000
p13	1.7759	1.2547	118.42	-100.522855499	-0.37	0.0000
p14	1.8259	1.2543	117.48	-100.522440417	-0.11	0.0000
p15	1.8759	1.2538	116.50	-100.522269557	-0.00	0.0000
ts*	1.8867	1.2537	116.30	-100.522263816	0.00	0.0000

```
-2 1
H B   1   1.8867 x   2   1.      1
90. O   2   1.2537  3   26.30  1
180.

1 2 S 30 +0.05

$nbo file=h_m_bo_m_eq archive $end

--Link1--
--Link1--
%mem=2gb
%nprocshared=8
%chk=h_m_bo_m_seg
#ub3lyp/6-311++g** guess=mix stable=opt

H(-)...BO(-), rhb=1.3259

-2 1
H B   1   rhb x   2   1.    1
90. O   2   rbo   3   hbo   1
180.
```

```
 rhb
1.3259 rbo
1.2095 hbo
90.
--Link1--
%mem=2gb
%nprocshared=8
%chk=h_m_bo_m_seg
#ub3lyp/6-311++g** guess=read nosymm opt=(z-matrix,calcfc)

H(-)...BO(-), rhb=1.3759

-2 1
H B   1   rhb x   2   1.    1
90. O   2   rbo   3   hbo   1
180.
 rbo
1.2095 hbo
90.  rhb
1.3759
--Link1--
%mem=2gb
%nprocshared=8
%chk=h_m_bo_m_seg
#ub3lyp/6-311++g** guess=read nosymm opt=(z-matrix,calcfc)

H(-)...BO(-), rhb=1.4259

-2 1
H B   1   rhb x   2   1.    1
90. O   2   rbo   3   hbo   1
180.
 rbo
1.2095 hbo
90.  rhb
1.4259
--Link1--
%mem=2gb
%nprocshared=8
%chk=h_m_bo_m_seg
#ub3lyp/6-311++g** guess=read nosymm opt=(z-matrix,calcfc)

H(-)...BO(-), rhb=1.4759

-2 1
H B   1   rhb x   2   1.    1
90. O   2   rbo   3   hbo   1
180.
 rbo
1.2095 hbo
90.  rhb
1.4759
--Link1--
%mem=2gb
%nprocshared=8
%chk=h_m_bo_m_seg
#ub3lyp/6-311++g** guess=read nosymm opt=(z-matrix,calcfc)

H(-)...BO(-), rhb=1.5259

-2 1
H B   1   rhb x   2   1.    1
90. O   2   rbo   3   hbo   1
180.
 rbo
1.2095 hbo
```

```
90.  rhb
1.5259
--Link1--
%mem=2gb
%nprocshared=8
%chk=h_m_bo_m_seg
#ub3lyp/6-311++g** guess=read nosymm opt=(z-matrix,calcfc)

H(-)...BO(-), rhb=1.5759

-2 1
H B   1   rhb x   2   1.    1
90. O   2   rbo   3   hbo   1
180.
 rbo
1.2095 hbo
90.  rhb
1.5759
--Link1--
%mem=2gb
%nprocshared=8
%chk=h_m_bo_m_seg
#ub3lyp/6-311++g** guess=read nosymm opt=(z-matrix,calcfc)

H(-)...BO(-), rhb=1.6259

-2 1
H B   1   rhb x   2   1.    1
90. O   2   rbo   3   hbo   1
180.
 rbo
1.2095 hbo
90.  rhb
1.6259
--Link1--
%mem=2gb
%nprocshared=8
%chk=h_m_bo_m_seg
#ub3lyp/6-311++g** guess=read nosymm opt=(z-matrix,calcfc)

H(-)...BO(-), rhb=1.6759

-2 1
H B   1   rhb x   2   1.    1
90. O   2   rbo   3   hbo   1
180.
 rbo
1.2095 hbo
90.  rhb
1.6759
--Link1--
%mem=2gb
%nprocshared=8
%chk=h_m_bo_m_seg
#ub3lyp/6-311++g** guess=read nosymm opt=(z-matrix,calcfc)

H(-)...BO(-), rhb=1.7259

-2 1
H B   1   rhb x   2   1.    1
90. O   2   rbo   3   hbo   1
180.
 rbo
1.2095 hbo
90.  rhb
1.7259
--Link1--
```

```
%mem=2gb
%nprocshared=8
%chk=h_m_bo_m_seg
#ub3lyp/6-311++g** guess=read nosymm opt=(z-matrix,calcfc)

H(-)...BO(-), rhb=1.7359

-2 1 H B   1   rhb x   2   1.
1   90. O   2   rbo   3   hbo
1   180.
 rbo
1.2095 hbo
90.  rhb
1.7359
--Link1--
%mem=2gb
%nprocshared=8
%chk=h_m_bo_m_seg
#ub3lyp/6-311++g** guess=read nosymm opt=(z-matrix,calcfc)

H(-)...BO(-), rhb=1.7459

-2 1
H
B   1   rhb
x   2   1.    1   90. O   2
rbo   3   hbo   1   180.
 rbo
1.2095 hbo
90.  rhb
1.7459
--Link1--
%mem=2gb
%nprocshared=8
%chk=h_m_bo_m_seg
#ub3lyp/6-311++g** guess=read nosymm opt=(z-matrix,calcfc)

H(-)...BO(-), rhb=1.7559

-2 1
H B   1   rhb x   2   1.    1
90. O   2   rbo   3   hbo   1
180.
 rbo
1.2095 hbo
90.  rhb
1.7559
--Link1--
%mem=2gb
%nprocshared=8
%chk=h_m_bo_m_seg
#ub3lyp/6-311++g** guess=read nosymm opt=(z-matrix,calcfc)

H(-)...BO(-), rhb=1.7659

-2 1
H B   1   rhb x   2   1.    1
90. O   2   rbo   3   hbo   1
180.
 rbo
1.2095 hbo
90.  rhb
1.7659
--Link1--
%mem=2gb
%nprocshared=8
%chk=h_m_bo_m_seg
```

```
#ub3lyp/6-311++g** guess=read nosymm opt=(z-matrix,calcfc)

H(-)...BO(-), rhb=1.7759

-2 1
H B   1   rhb x   2   1.    1
90. O   2   rbo   3   hbo   1
180.
 rbo
1.2095 hbo
90.  rhb
1.7759
--Link1--
%mem=2gb
%nprocshared=8
%chk=h_m_bo_m_seg
#ub3lyp/6-311++g** guess=read nosymm opt=(z-matrix,calcfc) H(-)...BO(-), rhb=1.8259

-2 1
H B   1   rhb x   2   1.    1
90. O   2   rbo   3   hbo   1
180.
 rbo
1.2095 hbo
90.  rhb
1.8259
--Link1--
%mem=2gb
%nprocshared=8
%chk=h_m_bo_m_seg
#ub3lyp/6-311++g** guess=read nosymm opt=(z-matrix,calcfc)

H(-)...BO(-), rhb=1.8759

-2 1
H B   1   rhb x   2   1.    1
90. O   2   rbo   3   hbo   1
180.
 rbo
1.2095 hbo
90.  rhb
1.8759
--Link1--
--Link1--
--Link1--
%mem=2gb
%nprocshared=8
%chk=h_m_bo_m_ts
#b3lyp/6-311++G** pop=nbo7read
  antielectrostatic pi-hole: H(-)...BO(-) t.s., E(RB3LYP) =  -100.522263816(e-
stable)  nu: -837,542,1594
 Vibrational temperatures:    780.00  2292.70
          (Kelvin)
 Zero-point correction=                           0.004865 (Hartree/Particle)
 Thermal correction to Energy=                    0.007896
 Thermal correction to Enthalpy=                  0.008840
Thermal correction to Gibbs Free Energy=        -0.016883
 Sum of electronic and zero-point Energies=           -100.517398
 Sum of electronic and thermal Energies=              -100.514368
 Sum of electronic and thermal Enthalpies=            -100.513424
 Sum of electronic and thermal Free Energies=         -100.539147
-2 1
H
B   1   1.8867
O   2   1.2537   1   116.30

$nbo file=h_m_bo_m_ts archive $end
```

```
--Link1--
%mem=2gb
%nprocshared=8
%chk=h_m_bo_m_sc
#b3lyp/6-311++G** opt=modredundant
  antielectrostatic pi-hole: H(-)...BO(-) t.s., E(RB3LYP) =  -
100.522263816  nu: -837,542,1594
 Vibrational temperatures:    780.00  2292.70
          (Kelvin)
 Zero-point correction=                           0.004865 (Hartree/Particle)  Thermal correction
to Energy=                    0.007896
 Thermal correction to Enthalpy=                  0.008840
Thermal correction to Gibbs Free Energy=        -0.016883
 Sum of electronic and zero-point Energies=          -100.517398
 Sum of electronic and thermal Energies=             -100.514368
 Sum of electronic and thermal Enthalpies=           -100.513424
 Sum of electronic and thermal Free Energies=        -100.539147
Summary of Optimized Potential Surface Scan (add -100.0 to energies):
                                 1                  2                  3                  4
5
     Eigenvalues --           -0.522264         -0.522374         -0.522697         -0.523213
-0.523901
                                (0.00)           (-0.07)           (-0.27)           (-0.60)
(-1.03)
           R1                  1.886700          1.936700          1.986700          2.036700
2.086700
           R2                  1.253700          1.252938          1.252504          1.251788
1.251074
           A1                116.300000        115.399931        114.583511        113.789098
113.052940
                                 6                  7                  8                  9
10
     Eigenvalues --           -0.524740         -0.525711         -0.526793         -0.527968
-0.529220
                               (-1.55)           (-2.16)           (-2.84)           (-3.58)
(-4.36)
           R1                  2.136700          2.186700          2.236700          2.286700
2.336700
           R2                  1.250384          1.249710          1.249055          1.248423
1.247817
           A1                112.368361        111.734649        111.150797        110.615610
110.127832
                                11                 12                 13                 14
15
     Eigenvalues --           -0.530533         -0.531895         -0.533293         -0.534717
-0.536159
                               (-5.19)           (-6.04)           (-6.92)           (-7.81)
(-8.72)
           R1                  2.386700          2.436700          2.486700          2.536700
2.586700
           R2                  1.247240          1.246693          1.246178          1.245694
1.245242
           A1                109.686239        109.289539        108.936330        108.625107
108.354157
                                16                 17                 18                 19
20
     Eigenvalues --           -0.537610         -0.539065         -0.540518         -0.541965
-0.543402
                               (-9.63)          (-10.54)          (-11.45)          (-12.36)
(-13.26)
           R1                  2.636700          2.686700          2.736700          2.786700
2.836700
           R2                  1.244820          1.244427          1.244064          1.243727
1.243416
           A1                108.121561        107.925400        107.763868        107.635151
107.537350
```

```
                           21
     Eigenvalues --           -0.544827
                              (-14.16)
           R1                 2.886700
           R2                 1.243129
           A1               107.468692
 GradGradGradGradGradGradGradGradGradGradGradGradGradGradGradGradGradGrad
 Summary of Optimized Potential Surface Scan (add -100.0 to energies):
                           1                  2                  3                  4
5
     Eigenvalues --           -0.522264          -0.522383          -0.522745          -0.523356
-0.524214
                              (0.00)            (-0.07)            (-0.30)            (-0.69)
(-1.22)
           R1                 1.886700           1.836700           1.786700           1.736700
1.686700
           R2                 1.253700           1.254395           1.254746           1.255277
1.255675
           A1               116.300000         117.242890         118.338521         119.423904
120.580021
                           6                  7                  8                  9
10
     Eigenvalues --           -0.525303          -0.526594          -0.528035          -0.529551
-0.531034
                              (-1.91)            (-2.72)            (-3.62)            (-4.57)
(-5.50)
           R1                 1.636700           1.586700           1.536700           1.486700
1.436700
           R2                 1.255917           1.256002           1.255917           1.255637
1.255144
           A1               121.807767         123.109262         124.489311         125.951718
127.500399
                          11                 12                 13                 14
15
     Eigenvalues --           -0.532336          -0.533266          -0.533575          -0.532955
-0.531059
                              (-6.32)            (-6.90)            (-7.10)            (-6.71)
(-5.52)
           R1                 1.386700           1.336700           1.286700           1.236700
1.186700
           R2                 1.254412           1.253417           1.251804           1.249569
1.245365
           A1               129.143880         130.894741         132.968050         135.331783
139.549149
                          16                 17                 18                 19
20
     Eigenvalues --           -0.527940          -0.523353          -0.515898          -0.504024
-0.486182
                              (-3.56)            (-0.68)            (+3.99)
           R1                 1.136700           1.086700           1.036700           0.986700
0.936700
           R2                 1.231439           1.224244           1.221187           1.220245
1.219595
           A1               152.175646         163.139440         170.960429         176.130420
177.237934
                          21
     Eigenvalues --           -0.460683
           R1                 0.886700
           R2                 1.217911
           A1               175.628360
 GradGradGradGradGradGradGradGradGradGradGradGradGradGradGradGradGradGrad
-2 1
H
B   1   1.8867
O   2   1.2537   1   116.30

1 2 S 20 +0.05
```

```
--Link1--
%mem=2gb
%nprocshared=8
%chk=h_m_bo_m_sc
#b3lyp/6-311++G** opt=modredundant
  antielectrostatic pi-hole: H(-)...BO(-) t.s., E(RB3LYP) =  -
100.522263816  nu: -837,542,1594
 Vibrational temperatures:    780.00  2292.70
          (Kelvin)
 Zero-point correction=                           0.004865 (Hartree/Particle)
 Thermal correction to Energy=                    0.007896
 Thermal correction to Enthalpy=                  0.008840
Thermal correction to Gibbs Free Energy=        -0.016883
 Sum of electronic and zero-point Energies=           -100.517398
Sum of electronic and thermal Energies=              -100.514368  Sum
of electronic and thermal Enthalpies=            -100.513424
 Sum of electronic and thermal Free Energies=         -100.539147

-2 1
H
B   1   1.8867
O   2   1.2537   1   116.30

1 2 S 20 -0.05

--Link1--
--Link1--
%mem=2gb
%nprocshared=8
%chk=h_m_bo_m
#b3lyp/6-311++G** pop=nbo7read

 antielectrostatic pi-hole: H(-)...BO(-), E(RB3LYP) =  -100.533580545  [-
7.10 kcal/mol w.r.t. t.s.; +97.07 w.r.t. H(-) + BO(-), -100.6882742]  nu:
652,1513,1832
 Vibrational temperatures:    938.40  2177.11  2635.35
          (Kelvin)
 Zero-point correction=                           0.009106 (Hartree/Particle)
 Thermal correction to Energy=                    0.012078
 Thermal correction to Enthalpy=                  0.013022
Thermal correction to Gibbs Free Energy=        -0.012008
 Sum of electronic and zero-point Energies=           -100.524475
 Sum of electronic and thermal Energies=              -100.521503
 Sum of electronic and thermal Enthalpies=            -100.520559
 Sum of electronic and thermal Free Energies=         -100.545589

-2 1
H
B   1   1.2924
O   2   1.2519   1   132.65

$nbo file=h_m_bo_m archive $end

--Link1--
**************************** F(-)...BO(-) Input Files ****************************
%mem=2gb
%nprocshared=8
%chk=f_m_bo_m_eq
#ub3lyp/6-311++G** guess=mix pop=nbo7read stable=opt

 AE pi-hole: F(-)...BO(-),

-2 1
F
B   1   1.2308 x   2
1.      1   90.
```

```
O   2   1.2218  3   90.    1   180.

$nbo file=f_m_bo_m_eq archive $end

--Link1--
%mem=2gb
%nprocshared=8
%chk=f_m_bo_m_eq
#ub3lyp/6-311++G** guess=read pop=nbo7read opt=calcall

 AE pi-hole: F(-)...BO(-), -199.902627194 (-11.01)
 nu: 362(2),891,1970
 Vibrational temperatures:    520.50   520.50  1282.27  2834.09
          (Kelvin)
 Zero-point correction=                           0.008166 (Hartree/Particle)
 Thermal correction to Energy=                    0.011280
 Thermal correction to Enthalpy=                  0.012224
Thermal correction to Gibbs Free Energy=        -0.013779
 Sum of electronic and zero-point Energies=           -199.894461
Sum of electronic and thermal Energies=              -199.891347  Sum
of electronic and thermal Enthalpies=            -199.890403
 Sum of electronic and thermal Free Energies=         -199.916407

-2 1
F
B   1   1.3025 x   2
1.      1   90.
O   2   1.2188  3   90.    1   180.

$nbo file=f_m_bo_m_eq archive $end

--Link1--
--Link1--
%mem=2gb
%nprocshared=8
%chk=f_m_bo_m_ts
#b3lyp/6-311++G** pop=nbo7read

 AE pi-hole: F(-)...BO(-) t.s., E(RB3LYP) =  -199.885078482(e-stable)[+98.97]
nu: -258,315,1594
  Vibrational temperatures:    452.73  2293.09
          (Kelvin)
 Zero-point correction=                           0.004348 (Hartree/Particle)
 Thermal correction to Energy=                    0.007586
 Thermal correction to Enthalpy=                  0.008530
Thermal correction to Gibbs Free Energy=        -0.020303
 Sum of electronic and zero-point Energies=           -199.880731
 Sum of electronic and thermal Energies=              -199.877493
 Sum of electronic and thermal Enthalpies=            -199.876549
 Sum of electronic and thermal Free Energies=         -199.905382

-2 1
F B   1
x    2    1.        1     90. O    2
1.2552   3   22.39  1  180.

$nbo file=f_m_bo_m_ts archive $end

--Link1--
--Link1--
**************************** F(-)...BO(-) IRC Input Files ***************************** %mem=2gb
%nprocshared=8
%chk=f_m_bo_m_irc
#b3lyp/6-311++G**  pop=nbo7read

  F(-)...BO(-) pt#=m10, E = -199.902127478, irc=-3.72738 (eq)
```

```
-2 1
     9    -0.391456     0.000000    -1.094223
     5    -0.299021    -0.000000     0.294841
8     0.670358     0.000000     1.097184

$nbo file=f_m_bo_m_m10 archive nrt plot $end

--Link1--
%mem=2gb
%nprocshared=8
%chk=f_m_bo_m_irc
#b3lyp/6-311++G**  pop=nbo7read

  F(-)...BO(-) pt#=m9, E = -199.901492393, irc=-3.38963

-2 1
     9    -0.382622     0.000000    -1.108859
     5    -0.330356     0.000000     0.329437
8     0.681860     0.000000     1.090255

$nbo file=f_m_bo_m_m9 archive nrt plot $end

--Link1--
%mem=2gb
%nprocshared=8
%chk=f_m_bo_m_irc
#b3lyp/6-311++G**  pop=nbo7read

  F(-)...BO(-) pt#=m8, E = -199.899443562, irc=-3.01365

-2 1
     9    -0.382640     0.000000    -1.137828
     5    -0.347400     0.000000     0.369198
     8     0.693612     0.000000     1.097296

$nbo file=f_m_bo_m_m8 archive nrt plot $end

--Link1--
%mem=2gb
%nprocshared=8
%chk=f_m_bo_m_irc
#b3lyp/6-311++G**  pop=nbo7read

  F(-)...BO(-) pt#=m7, E = -199.896590170, irc=-2.63705

-2 1
     9    -0.384012     0.000000    -1.168168
     5    -0.360032     0.000000     0.409196
     8     0.703933    -0.000000     1.105824

$nbo file=f_m_bo_m_m7 archive nrt plot $end

--Link1--
%mem=2gb
%nprocshared=8
%chk=f_m_bo_m_irc
#b3lyp/6-311++G**  pop=nbo7read

  F(-)...BO(-) pt#=m6, E = -199.893603232, irc=-2.26033

-2 1
     9    -0.385981     0.000000    -1.198916
     5    -0.370076    -0.000000     0.449716
     8     0.713186     0.000000     1.114450

$nbo file=f_m_bo_m_m6 archive nrt plot $end
```

```
--Link1--
%mem=2gb
%nprocshared=8
%chk=f_m_bo_m_irc
#b3lyp/6-311++G**  pop=nbo7read

  F(-)...BO(-) pt#=m5, E = -199.890895889, irc=-1.88363

-2 1
     9    -0.388342     0.000000    -1.229997
     5    -0.377954     0.000000     0.490448
     8     0.721410    -0.000000     1.123356

$nbo file=f_m_bo_m_m5 archive nrt plot $end

--Link1--
%mem=2gb
%nprocshared=8
%chk=f_m_bo_m_irc
#b3lyp/6-311++G**  pop=nbo7read

  F(-)...BO(-) pt#=m4, E = -199.888673833, irc=-1.50692

-2 1
     9    -0.390976     0.000000    -1.261241
     5    -0.384551    -0.000000     0.531275
     8     0.729080     0.000000     1.132370

$nbo file=f_m_bo_m_m4 archive nrt plot $end

--Link1--
%mem=2gb
%nprocshared=8
%chk=f_m_bo_m_irc
#b3lyp/6-311++G**  pop=nbo7read

  F(-)...BO(-) pt#=m3, E = -199.887007510, irc=-1.13020

-2 1
     9    -0.393771     0.000000    -1.292562
     5    -0.390821     0.000000     0.572007
     8     0.736715    -0.000000     1.141532

$nbo file=f_m_bo_m_m3 archive nrt plot $end

--Link1--
%mem=2gb
%nprocshared=8
%chk=f_m_bo_m_irc
#b3lyp/6-311++G**  pop=nbo7read

  F(-)...BO(-) pt#=m2, E = -199.885890124, irc=-0.75350

-2 1
     9    -0.396770     0.000000    -1.324135
     5    -0.395888    -0.000000     0.612530
     8     0.743765     0.000000     1.151165

$nbo file=f_m_bo_m_m2 archive nrt plot $end

--Link1--
%mem=2gb
%nprocshared=8
%chk=f_m_bo_m_irc
#b3lyp/6-311++G**  pop=nbo7read
```

```
  F(-)...BO(-) pt#=m1, E = , irc=-0.37681

-2 1
     9    -0.400016     0.000000    -1.355847
     5    -0.400065     0.000000     0.652914
     8     0.750495     0.000000     1.161043

$nbo file=f_m_bo_m_m1 archive nrt plot $end

--Link1--
%mem=2gb
%nprocshared=8
%chk=f_m_bo_m_irc
#b3lyp/6-311++G**  pop=nbo7read

 AEPH: F(-)...BO(-) t.s., E = -199.885078482, irc=0.0000

-2 1
F
B   1   2.0808
O   2   1.2552   1   112.39

$nbo file=f_m_bo_m_ts archive nrt plot $end

--Link1--
%mem=2gb
%nprocshared=8
%chk=f_m_bo_m_irc
#b3lyp/6-311++G**  pop=nbo7read

  F(-)...BO(-) pt#=p1, E = -199.885246655, irc=0.37680 -2 1
     9    -0.407037     0.000000    -1.419553
     5    -0.406988     0.000000     0.733286
     8     0.763599     0.000000     1.181392

$nbo file=f_m_bo_m_p1 archive nrt plot $end

--Link1--
%mem=2gb
%nprocshared=8
%chk=f_m_bo_m_irc
#b3lyp/6-311++G**  pop=nbo7read

  F(-)...BO(-) pt#=p2, E = -199.885710447, irc=0.75349

-2 1
     9    -0.410720    -0.000000    -1.451579
     5    -0.409719    -0.000000     0.773132
     8     0.769854     0.000000     1.192017

$nbo file=f_m_bo_m_p2 archive nrt plot $end

--Link1--
%mem=2gb
%nprocshared=8
%chk=f_m_bo_m_irc
#b3lyp/6-311++G**  pop=nbo7read

  F(-)...BO(-) pt#=p3, E = -199.886415317, irc=1.13020

-2 1
     9    -0.414558     0.000000    -1.483763
     5    -0.411685    -0.000000     0.812719
     8     0.775765    -0.000000     1.203026
```

```
$nbo file=f_m_bo_m_p3 archive nrt plot $end

--Link1--
%mem=2gb
%nprocshared=8
%chk=f_m_bo_m_irc
#b3lyp/6-311++G**  pop=nbo7read

  F(-)...BO(-) pt#=p4, E = -199.887316695, irc=1.50694

-2 1
     9    -0.418546     0.000000    -1.515915
     5    -0.413180     0.000000     0.852438
     8     0.781530    -0.000000     1.213870

$nbo file=f_m_bo_m_p4 archive nrt plot $end

--Link1--
%mem=2gb
%nprocshared=8
%chk=f_m_bo_m_irc
#b3lyp/6-311++G**  pop=nbo7read

  F(-)...BO(-) pt#=p5, E = -199.888377256, irc=1.88367

-2 1
     9    -0.422584     0.000000    -1.548159
     5    -0.414496     0.000000     0.891926
     8     0.787232    -0.000000     1.224992

$nbo file=f_m_bo_m_p5 archive nrt plot $end

--Link1--
%mem=2gb
%nprocshared=8
%chk=f_m_bo_m_irc
#b3lyp/6-311++G**  pop=nbo7read

  F(-)...BO(-) pt#=p6, E = -199.889567726, irc=2.26041

-2 1
     9    -0.426737     0.000000    -1.580513
     5    -0.415142     0.000000     0.931205
     8     0.792611    -0.000000     1.236401

$nbo file=f_m_bo_m_p6 archive nrt plot $end

--Link1--
%mem=2gb
%nprocshared=8
%chk=f_m_bo_m_irc
#b3lyp/6-311++G**  pop=nbo7read

  F(-)...BO(-) pt#=p7, E = -199.890864069, irc=2.63715

-2 1
     9    -0.430962     0.000000    -1.612856
     5    -0.415345     0.000000     0.970572
     8     0.797768    -0.000000     1.247718

$nbo file=f_m_bo_m_p7 archive nrt plot $end

--Link1--
%mem=2gb
%nprocshared=8
%chk=f_m_bo_m_irc
```

```
#b3lyp/6-311++G**  pop=nbo7read

  F(-)...BO(-) pt#=p8, E = -199.892244116, irc=3.01389

-2 1
     9    -0.435252     0.000000    -1.645279
     5    -0.415381    -0.000000     1.009709
     8     0.802890    -0.000000     1.259293

$nbo file=f_m_bo_m_p8 archive nrt plot $end

--Link1--
%mem=2gb
%nprocshared=8
%chk=f_m_bo_m_irc
#b3lyp/6-311++G**  pop=nbo7read

  F(-)...BO(-) pt#=p9, E = -199.893690828, irc=3.39063

-2 1
     9    -0.439634     0.000000    -1.677789
     5    -0.414826    -0.000000     1.048651
8     0.807712    -0.000000     1.271116

$nbo file=f_m_bo_m_p9 archive nrt plot $end

--Link1--
%mem=2gb
%nprocshared=8
%chk=f_m_bo_m_irc
#b3lyp/6-311++G**  pop=nbo7read

  F(-)...BO(-) pt#=p10, E = -199.895189470, irc=3.76738

-2 1
     9    -0.444076     0.000000    -1.710300
     5    -0.413821     0.000000     1.087627
     8     0.812298    -0.000000     1.282901

$nbo file=f_m_bo_m_p10 archive nrt plot $end

--Link1--
%mem=2gb
%nprocshared=8
%chk=f_m_bo_m_irc
#b3lyp/6-311++G**  pop=nbo7read

  F(-)...BO(-) pt#=p11, E = -199.896726151, irc=4.14413

-2 1
     9    -0.448589     0.000000    -1.742885
     5    -0.412672    -0.000000     1.126363
     8     0.816866    -0.000000     1.294946

$nbo file=f_m_bo_m_p11 archive nrt plot $end

--Link1--
%mem=2gb
%nprocshared=8
%chk=f_m_bo_m_irc
#b3lyp/6-311++G**  pop=nbo7read

  F(-)...BO(-) pt#=p12, E = -199.898291441, irc=4.52087

-2 1
     9    -0.453186     0.000000    -1.775552
```

```
     5    -0.410975     0.000000     1.164871
     8     0.821159    -0.000000     1.307253

$nbo file=f_m_bo_m_p12 archive nrt plot $end

--Link1--
%mem=2gb
%nprocshared=8
%chk=f_m_bo_m_irc
#b3lyp/6-311++G**  pop=nbo7read

  F(-)...BO(-) pt#=p13, E = -199.899877669, irc=4.89762

-2 1
     9    -0.457837    -0.000000    -1.808224
     5    -0.408844    -0.000000     1.203380
     8     0.825217    -0.000000     1.319552

$nbo file=f_m_bo_m_p13 archive nrt plot $end

--Link1--
%mem=2gb
%nprocshared=8
%chk=f_m_bo_m_irc
#b3lyp/6-311++G**  pop=nbo7read

  F(-)...BO(-) pt#=p14, E = -199.901477130, irc=5.27437

-2 1
     9    -0.462550     0.000000    -1.840962
     5    -0.406598    -0.000000     1.241657
     8     0.829269     0.000000     1.332093

$nbo file=f_m_bo_m_p14 archive nrt plot $end

--Link1--
%mem=2gb
%nprocshared=8
%chk=f_m_bo_m_irc
#b3lyp/6-311++G**  pop=nbo7read

  F(-)...BO(-) pt#=p15, E = -199.903084703, irc=5.65111

-2 1
     9    -0.467333     0.000000    -1.873769
     5    -0.403859     0.000000     1.279706
     8     0.833066    -0.000000     1.344881

$nbo file=f_m_bo_m_p15 archive nrt plot $end

--Link1--
%mem=2gb
%nprocshared=8
%chk=f_m_bo_m_irc
#b3lyp/6-311++G**  pop=nbo7read

  F(-)...BO(-) pt#=p16, E = -199.904696098, irc=6.02786

-2 1
     9    -0.472159    -0.000000    -1.906578
     5    -0.400711    -0.000000     1.317746
     8     0.836631     0.000000     1.357665

$nbo file=f_m_bo_m_p16 archive nrt plot $end

--Link1--
```

```
%mem=2gb
%nprocshared=8
%chk=f_m_bo_m_irc
#b3lyp/6-311++G**  pop=nbo7read

  F(-)...BO(-) pt#=p17, E = -199.906307103, irc=6.40461

-2 1
     9    -0.477037    -0.000000    -1.939444
     5    -0.397477    -0.000000     1.355571
     8     0.840199     0.000000     1.370669

$nbo file=f_m_bo_m_p17 archive nrt plot $end

--Link1--
%mem=2gb
%nprocshared=8
%chk=f_m_bo_m_irc
#b3lyp/6-311++G**  pop=nbo7read

  F(-)...BO(-) pt#=p18, E = -199.907990263, irc=6.79905

-2 1
      1          9           0       -0.482206    0.000000   -1.973893
2          5           0       -0.393518    0.000000    1.395009
      3          8           0        0.843613   -0.000000    1.384441

$nbo file=f_m_bo_m_p18 archive nrt plot $end

--Link1--
%mem=2gb
%nprocshared=8
%chk=f_m_bo_m_irc
#b3lyp/6-311++G**  pop=nbo7read

  F(-)...BO(-) pt#=p19, E = -199.909664887, irc=7.19351

-2 1
1          9           0       -0.487430   -0.000000   -2.008387
2          5           0       -0.389471   -0.000000    1.434267       3          8
           0        0.847034    0.000000    1.398393

$nbo file=f_m_bo_m_p19 archive nrt plot $end

--Link1--
%mem=2gb
%nprocshared=8
%chk=f_m_bo_m_irc
#b3lyp/6-311++G**  pop=nbo7read

  F(-)...BO(-) pt#=p20, E = -199.911328267, irc=7.58798

-2 1
1          9           0       -0.492725    0.000000   -2.042951
2          5           0       -0.384960    0.000000    1.473230       3          8
           0        0.850219   -0.000000    1.412640

$nbo file=f_m_bo_m_p20 archive nrt plot $end

--Link1--
***************************** F(-)...BO(-) MP2 Input Files *****************************
%mem=2gb
%nprocshared=8
%chk=f_m_bo_m_eq_mp2
#mp2/6-311++G** pop=nbo7read density=current opt=calcall
```

```
 AE pi-hole: F(-)...BO(-), -0.19942853513464D+03(-15.37) [+85.94]
 [w.r.t. -199.5654846]
nu: 428(2),896,1967
 Vibrational temperatures:    615.80   615.80  1289.44  2830.01
          (Kelvin)
 Zero-point correction=                           0.008473 (Hartree/Particle)
 Thermal correction to Energy=                    0.011455
 Thermal correction to Enthalpy=                  0.012399
Thermal correction to Gibbs Free Energy=        -0.013375
 Sum of electronic and zero-point Energies=           -199.420062
 Sum of electronic and thermal Energies=              -199.417080
 Sum of electronic and thermal Enthalpies=            -199.416136
Sum of electronic and thermal Free Energies=         -199.441910

-2 1
F
B   1   1.3043 x   2
1.      1   90.
O   2   1.2290  3   90.    1   180.

$nbo file=f_m_bo_m_eq_mp2 archive $end

--Link1--
--Link1--
%mem=2gb
%nprocshared=8
%chk=f_m_bo_m_ts_eq
#mp2/6-311++G** pop=nbo7read density=current opt=(ts,calcall)

 AE pi-hole: F(-)...BO(-) t.s., -0.19940403589904D+03
nu: -301,298,1581
 Vibrational temperatures:    428.13  2274.50
          (Kelvin)
 Zero-point correction=                           0.004279 (Hartree/Particle)
 Thermal correction to Energy=                    0.007539
 Thermal correction to Enthalpy=                  0.008483
Thermal correction to Gibbs Free Energy=        -0.020427
 Sum of electronic and zero-point Energies=           -199.399757
 Sum of electronic and thermal Energies=              -199.396497
 Sum of electronic and thermal Enthalpies=            -199.395553
Sum of electronic and thermal Free Energies=         -199.424463
-2 1
                9                   -1.106959   -0.987933   -0.000000
5                  0.000000    0.811434    0.000000                 8
1.245329    0.604278    0.000000

$nbo file=f_m_bo_m_ts_mp2 archive fixdm $end
--Link1--
**************************** F(-)...BO(-) Segment Scan Input Files ****************************
%mem=2gb
%nprocshared=8
%chk=f_m_bo_m_sc
#b3lyp/6-311++G** opt=modredundant
  antielectrostatic pi-hole: F(-)...BO(-) t.s., E(RB3LYP) =  -
199.885078482   nu: -258,315,1594
  Vibrational temperatures:    452.73  2293.09
          (Kelvin)
 Zero-point correction=                           0.004348 (Hartree/Particle)
 Thermal correction to Energy=                    0.007586
 Thermal correction to Enthalpy=                  0.008530
Thermal correction to Gibbs Free Energy=        -0.020303
 Sum of electronic and zero-point Energies=           -199.880731
 Sum of electronic and thermal Energies=              -199.877493
 Sum of electronic and thermal Enthalpies=            -199.876549
 Sum of electronic and thermal Free Energies=         -199.905382
Summary of Optimized Potential Surface Scan (add -199.0 to energies):
```

	1	2	3	4	5
Eigenvalues --	-0.885078	-0.885164	-0.885409	-0.885792	-0.886297
	(0.00)	(-0.05)	(-0.21)	(-0.45)	(-0.76)
R1	2.080800	2.130800	2.180800	2.230800	2.280800
R2	1.255200	1.253884	1.253115	1.252122	1.251169
A1	112.390000	111.884372	111.359769	110.898823	110.487453
	6	7	8	9	10
Eigenvalues --	-0.886907	-0.887611	-0.888395	-0.889249	-0.890165
	(-1.15)	(-1.59)	(-2.08)	(-2.62)	(-3.19)
R1	2.330800	2.380800	2.430800	2.480800	2.530800
R2	1.250300	1.249504	1.248771	1.248094	1.247472
A1	110.114721	109.777800	109.475263	109.205689	108.968056
	11	12	13	14	15
Eigenvalues --	-0.891135	-0.892150	-0.893205	-0.894294	-0.895411
	(-3.80)	(-4.44)	(-5.10)	(-5.78)	(-6.48)
R1	2.580800	2.630800	2.680800	2.730800	2.780800
R2	1.246898	1.246370	1.245885	1.245439	1.245029
A1	108.760377	108.579806	108.424401	108.292702	108.182673
	16	17	18	19	20
Eigenvalues --	-0.896552	-0.897712	-0.898888	-0.900077	-0.901276
	(-7.20)	(-7.92)	(-8.67)	(-9.41)	(-10.16)
R1	2.830800	2.880800	2.930800	2.980800	3.030800
R2	1.244651	1.244303	1.244296	1.243980	1.243689
A1	108.092345	108.020161	108.021315	107.964111	107.918668
	21	22	23	24	25
Eigenvalues --	-0.902482	-0.903694	-0.904909	-0.906126	-0.907343
	(-10.92)	(-11.68)	(-12.44)	(-13.21)	(-13.97)
R1	3.080800	3.130800	3.180800	3.230800	3.280800
R2	1.243418	1.243166	1.242930	1.242709	1.242502
A1	107.884110	107.859588	107.843768	107.835307	107.833632
	26	27	28	29	30
Eigenvalues --	-0.908559	-0.909772	-0.910982	-0.912186	-0.913384
	(-14.73)	(-15.50)			
R1	3.330800	3.380800	3.430800	3.480800	3.530800

```
           R2                 1.242308         1.242125         1.241953         1.241789
1.241634
           A1               107.838453       107.849107       107.864673       107.884536
107.908760
                              31                32                33                34
35
     Eigenvalues --          -0.914576        -0.915760        -0.916935        -0.918101
-0.919257
           R1                 3.580800         3.630800         3.680800         3.730800
3.780800
           R2                 1.241488         1.241348         1.241215         1.241088
1.240968
           A1               107.937531       107.970586       108.007225       108.046945
108.089917
                              36
     Eigenvalues --          -0.920402
           R1                 3.830800
           R2                 1.240853
           A1               108.136464
 GradGradGradGradGradGradGradGradGradGradGradGradGradGradGradGradGradGradGrad

 GradGradGradGradGradGradGradGradGradGradGradGradGradGradGradGradGradGradGrad
-2 1
F
B   1   2.0808
O   2   1.2552   1   112.39

1 2 S 35 +0.05

--Link1--
--Link1--
%mem=2gb
%nprocshared=8
%chk=f_m_bo_m_sc
#b3lyp/6-311++G** opt=(modredundant,calcall)
  antielectrostatic pi-hole: F(-)...BO(-) t.s., E(RB3LYP) =  -
199.885078482   nu: -258,315,1594
  Vibrational temperatures:    452.73  2293.09
          (Kelvin)
 Zero-point correction=                           0.004348 (Hartree/Particle)
 Thermal correction to Energy=                    0.007586
 Thermal correction to Enthalpy=                  0.008530
Thermal correction to Gibbs Free Energy=        -0.020303
 Sum of electronic and zero-point Energies=           -199.880731
 Sum of electronic and thermal Energies=              -199.877493
 Sum of electronic and thermal Enthalpies=            -199.876549
 Sum of electronic and thermal Free Energies=         -199.905382
 2.0808  1.2552  112.39  -199.885078482 (0.00)
 2.0308  1.2563  113.00  -199.885172394 (-0.06)
 1.9808  1.2575  113.67  -199.885468357 (-0.24)
 1.9308  1.2587  114.42  -199.885989025 (-0.57)
 1.8808  1.2599  115.25  -199.886754804 (-1.05)
1.8308  1.2611  116.18  -199.887782071 (-1.70)
 1.7808  1.2623  117.22  -199.889078989 (-2.51)
1.7308  1.2632  118.41  -199.890640821 (-3.49)
 1.6808  1.2640  119.76  -199.892444065 (-4.62)
 1.6308  1.2645  121.31  -199.894437739 (-5.87)
 1.5808  1.2646  123.12  -199.896533635 (-7.19)
 1.5308  1.2640  125.37  -199.898594519 (-8.48)
 1.4808  1.2626  128.01  -199.900418187 (-9.63)
 1.4308  1.2601  131.34  -199.901724365 (-10.45)
 1.3808  1.2556  135.86  -199.902145143 (-10.71)
*1.3025  1.2188  180.00  -199.902627194 (-11.01)
 1.2969  1.2159  180.00  -199.902051061 (-10.65)
 1.2808  1.2198  180.00  -199.902271301 (-10.79)
 1.2308  1.2218  180.00  -199.898190699 (-8.23)
 1.1808  1.2245  180.00  -199.887991058 (-1.83)
```

```
 1.1308  1.2279  180.00  -199.869214055 (+9.95)
 1.0808  1.2321  180.00  -199.838610662 (+29.16)
 1.0308  1.2371  180.00  -199.791863615
 0.9808  1.2428  180.00  -199.723207967

-2 1
F
B   1   1.2808 x   2   1.       1
90. O   2   1.2198   3    90.  1
180.

1 2 S 20 -0.05

--Link1--
--Link1--
%mem=2gb
%nprocshared=8
%chk=f_m_bo_m_eq
#b3lyp/6-311++G** pop=nbo7read

 antielectrostatic pi-hole: F(-)...BO(-), E(RB3LYP) =  -199.902155371  [-
10.72 kcal/mol w.r.t. t.s., +88.26 w.r.t. F(-) + BO(-), -200.0428038]  nu:
307,720,1619
 Vibrational temperatures:    441.19  1035.45  2329.92
          (Kelvin)
 Zero-point correction=                           0.006027 (Hartree/Parti$
 Thermal correction to Energy=                    0.009380
 Thermal correction to Enthalpy=                  0.010324
Thermal correction to Gibbs Free Energy=        -0.017855
 Sum of electronic and zero-point Energies=           -199.896128
Sum of electronic and thermal Energies=              -199.892776
 Sum of electronic and thermal Enthalpies=            -199.891831
 Sum of electronic and thermal Free Energies=         -199.920010

-2 1
F
B   1   1.3871
O   2   1.2563   1   135.19

$nbo file=f_m_bo_m_eq archive $end

--Link1--
**************************** H(-)...CN(-) Input Files ***************************** %mem=2gb
%nprocshared=8
%chk=h_m_cn_m_eq
#ub3lyp/6-311++G** scf=qc stable=opt

 AEHB: H(-)...CN(-) pt.#m11, E = -93.2701684598(-26.58), <S**2>= 0.0000, R(CH)=1.0725
-2 1
H C   1   rch x   2   1.    1
90. N   2   rcn   3   xcn   1
180.
 rcn
1.1511 xcn
90.00 rch
1.0725
--Link1--
%mem=2gb
%nprocshared=8
%chk=h_m_cn_m_eq
#ub3lyp/6-311++G**  guess=read pop=nbo7read scf=qc

 AEHB: H(-)...CN(-) eq, E(UB3LYP) =  -93.2705185229(-26.80), R(CH)=1.0504, S^2 = 0.00
nu: 923(2),2181,3531
 Vibrational temperatures:   1328.15  1328.15  3138.13  5079.76
          (Kelvin)
 Zero-point correction=                           0.017218 (Hartree/Particle)
```

```
 Thermal correction to Energy=                    0.019678
 Thermal correction to Enthalpy=                  0.020622
Thermal correction to Gibbs Free Energy=        -0.002116
 Sum of electronic and zero-point Energies=           -93.253300
Sum of electronic and thermal Energies=              -93.250841
 Sum of electronic and thermal Enthalpies=            -93.249896
 Sum of electronic and thermal Free Energies=         -93.272635

-2 1
                1                   0.000000    0.000000   -1.550772
                6                   0.000000    0.000000   -0.500335
7                   0.000000    0.000000    0.650398

$nbo file=h_m_cn_m_eq archive $end

--Link1--
--Link1--
%mem=2gb
%nprocshared=8
%chk=h_m_cn_m_ts
#ub3lyp/6-311++G** guess=mix stable=opt

 AEHB: H(-)...CN(-) t.s., E(UB3LYP) =  -93.2278127967, <S**2>= 0.5590
R(CH)=1.6225, R(CN)=1.1928, TH(H-C-N)=126.27  nu: -1373,551,1931
 Vibrational temperatures:    793.28  2778.28
          (Kelvin)
 Zero-point correction=                           0.005655 (Hartree/Particle)
 Thermal correction to Energy=                    0.008677
 Thermal correction to Enthalpy=                  0.009622
Thermal correction to Gibbs Free Energy=        -0.015674
 Sum of electronic and zero-point Energies=           -93.222158
Sum of electronic and thermal Energies=              -93.219135
 Sum of electronic and thermal Enthalpies=            -93.218191
 Sum of electronic and thermal Free Energies=         -93.243487
 [EVERY POINT BELOW IS GUARANTEED E-STABLE; ALL POINTS Z-MATRIX OPTIMIZED]

-2 1
H C   1   rch x   2   1.    1
90. N   2   rcn   3   xcn   1
180.
 rcn
1.1928 xcn
36.27 rch
1.6225

$nbo file=h_m_cn_m_ts archive $end

--Link1--
%mem=2gb
%nprocshared=8
%chk=h_m_cn_m_ts
#ub3lyp/6-311++G** guess=read pop=nbo7read

 AEHB: H(-)...CN(-) t.s., E(UB3LYP) =  -93.2278127967, <S**2>= 0.5590
R(CH)=1.6225, R(CN)=1.1928, TH(H-C-N)=126.27  nu: -1373,551,1931
 Vibrational temperatures:    793.28  2778.28
          (Kelvin)
 Zero-point correction=                           0.005655 (Hartree/Particle)
Thermal correction to Energy=                    0.008677
 Thermal correction to Enthalpy=                  0.009622
Thermal correction to Gibbs Free Energy=        -0.015674
 Sum of electronic and zero-point Energies=           -93.222158
 Sum of electronic and thermal Energies=              -93.219135
 Sum of electronic and thermal Enthalpies=            -93.218191
 Sum of electronic and thermal Free Energies=         -93.243487

-2 1
```

```
H C   1   rch x   2   1.    1
90. N   2   rcn   3   xcn   1
180.
 rcn
1.1928 xcn
36.27 rch
1.6225

$nbo file=h_m_cn_m_ts archive nrt plot $end

--Link1--
**************************** H(-)...CN(-) MP2 Input Files **************************** %mem=2gb
%nprocshared=8
%chk=h_m_cn_m_eq_mp2
#uhf/6-311++G** scf=qc stable=opt

 AEHB: H(-)...CN(-) guess,

-2 1
                1                   0.000000    0.000000   -1.550772
                6                   0.000000    0.000000   -0.500335
7                   0.000000    0.000000    0.650398

--Link1--
%mem=2gb
%nprocshared=8
%chk=h_m_cn_m_eq_mp2
#ump2/6-311++G**  guess=read pop=nbo7read scf=qc opt=calcall

 AEHB: H(-)...CN(-) eq, -0.92996381621378D+02(-37.24),(S**2,1)=  0.73041D+00
nu: 900(2),2263,3463
 Vibrational temperatures:   1295.51  1295.51  3255.44  4982.88
          (Kelvin)
 Zero-point correction=                           0.017147 (Hartree/Particle)
 Thermal correction to Energy=                    0.019616
 Thermal correction to Enthalpy=                  0.020560
Thermal correction to Gibbs Free Energy=        -0.002220
 Sum of electronic and zero-point Energies=            -92.979234
Sum of electronic and thermal Energies=               -92.976766
 Sum of electronic and thermal Enthalpies=             -92.975822
Sum of electronic and thermal Free Energies=          -92.998602
-2 1
                1                   0.000000    0.000000   -1.570134
                6                   0.000000    0.000000   -0.509650
7                   0.000000    0.000000    0.661148

$nbo file=h_m_cn_m_eq archive fixdm $end

--Link1--
%mem=2gb
%nprocshared=8
%chk=h_m_cn_m_ts_mp2
#uhf/6-311++G** guess=mix stable=opt

 AEHB: H(-)...CN(-) t.s.,

-2 1
H C   1   rch x   2
1.    1   90.
N   2   rcn   3   xcn   1  180.
 rcn
1.1928 xcn
36.27 rch
1.6225
--Link1--
%mem=2gb
%nprocshared=8
```

```
%chk=h_m_cn_m_ts_mp2
#ump2/6-311++G** guess=read pop=nbo7read density=current opt=(ts,calcall)
 AEHB: H(-)...CN(-) t.s., -0.92937040715227D+02, (S**2,1)=  0.91807D+00
nu: -1517,596,2216
 Vibrational temperatures:    858.44  3188.08
          (Kelvin)
 Zero-point correction=                           0.006407 (Hartree/Particle)
 Thermal correction to Energy=                    0.009402
 Thermal correction to Enthalpy=                  0.010346
Thermal correction to Gibbs Free Energy=        -0.014760
 Sum of electronic and zero-point Energies=           -92.930633
Sum of electronic and thermal Energies=              -92.927639
 Sum of electronic and thermal Enthalpies=            -92.926695
Sum of electronic and thermal Free Energies=         -92.951800

-2 1
                1                  -1.035558    1.630862   -0.000000
                6                   0.079658    0.516996   -0.000000
7                    0.079658   -0.676119    0.000000

$nbo file=h_m_cn_m_ts_mp2 archive nrt plot fixdm $end

--Link1--
***************************** H(-)...CN(-) Segment Scan Input Files *****************************
%mem=2gb
%nprocshared=8
%chk=h_m_cn_m_sc
#ub3lyp/6-311++G** guess=mix stable=opt

 AEHB: H(-)...CN(-) t.s., E(UB3LYP) =  -93.2278127967, <S**2>= 0.5590
R(CH)=1.6225, R(CN)=1.1928, TH(H-C-N)=126.27  nu: -1373,551,1931
 Vibrational temperatures:    793.28  2778.28
          (Kelvin)
 Zero-point correction=                           0.005655 (Hartree/Particle)
 Thermal correction to Energy=                    0.008677
Thermal correction to Enthalpy=                 0.009622  Thermal
correction to Gibbs Free Energy=        -0.015674
 Sum of electronic and zero-point Energies=           -93.222158
Sum of electronic and thermal Energies=              -93.219135  Sum
of electronic and thermal Enthalpies=            -93.218191
 Sum of electronic and thermal Free Energies=         -93.243487
 [EVERY POINT BELOW IS GUARANTEED E-STABLE; ALL POINTS Z-MATRIX OPTIMIZED]

-2 1
H
C   1   rch  x   2
1.    1   90.
N   2   rcn   3   xcn   1  180.
 rcn
1.1928 xcn
36.27 rch
1.6225

$nbo file=h_m_cn_m_ts archive $end

--Link1--
%mem=2gb
%nprocshared=8
%chk=h_m_cn_m_sc
#ub3lyp/6-311++G** guess=read pop=nbo7read

 AEHB: H(-)...CN(-) t.s., E(UB3LYP) =  -93.2278127967, <S**2>= 0.5590
R(CH)=1.6225, R(CN)=1.1928, TH(H-C-N)=126.27  nu: -1373,551,1931
 Vibrational temperatures:    793.28  2778.28
          (Kelvin)
 Zero-point correction=                           0.005655 (Hartree/Particle)
 Thermal correction to Energy=                    0.008677
```

```
 Thermal correction to Enthalpy=                   0.009622
Thermal correction to Gibbs Free Energy=        -0.015674
 Sum of electronic and zero-point Energies=            -93.222158
Sum of electronic and thermal Energies=               -93.219135
 Sum of electronic and thermal Enthalpies=             -93.218191
 Sum of electronic and thermal Free Energies=          -93.243487

-2 1
H C   1   rch  x   2   1.    1
90. N   2   rcn   3   xcn   1
180.
 rcn
1.1928 xcn
36.27 rch
1.6225

$nbo file=h_m_cn_m_ts archive $end

--Link1--
%mem=2gb
%nprocshared=8
%chk=h_m_cn_m_sc
#ub3lyp/6-311++G**  guess=read pop=nbo7read

 AEHB: H(-)...CN(-) pt.#m1, E = -93.2281227676(-0.19), <S**2>= 0.6480, R(CH)=1.5725
-2 1
H C   1   rch  x   2   1.    1
90. N   2   rcn   3   xcn   1
180.
 rcn
1.1943 xcn
36.86 rch
1.5725

$nbo file=h_m_cn_m_m1 archive $end

--Link1--
%mem=2gb
%nprocshared=8
%chk=h_m_cn_m_sc
#ub3lyp/6-311++G**  guess=read pop=nbo7read

 AEHB: H(-)...CN(-) pt.#m2, E = -93.2290713466(-0.79), <S**2>= 0.7287, R(CH)=1.5225
-2 1
H C   1   rch  x   2   1.    1
90. N   2   rcn   3   xcn   1
180.
 rcn
1.1961 xcn
37.42 rch
1.5225

$nbo file=h_m_cn_m_m2 archive $end

--Link1--
%mem=2gb
%nprocshared=8
%chk=h_m_cn_m_sc
#ub3lyp/6-311++G**  guess=read pop=nbo7read

 AEHB: H(-)...CN(-) pt.#m3, E = -93.2306627764(-1.79), <S**2>= 0.7983, R(CH)=1.4725
-2 1
H C   1   rch  x   2   1.    1
90. N   2   rcn   3   xcn   1
180.
 rcn
1.1978 xcn
```

```
38.10 rch
1.4725

$nbo file=h_m_cn_m_m3 archive $end

--Link1--
%mem=2gb
%nprocshared=8
%chk=h_m_cn_m_sc
#ub3lyp/6-311++G**  guess=read pop=nbo7read

 AEHB: H(-)...CN(-) pt.#m4, E = -93.2328540781(-3.16), <S**2>= 0.8568, R(CH)=1.4225
-2 1
H C   1   rch  x   2   1.    1
90. N   2   rcn   3   xcn   1
180.
 rcn
1.2000 xcn
38.65 rch
1.4225

$nbo file=h_m_cn_m_m4 archive $end

--Link1--
%mem=2gb
%nprocshared=8
%chk=h_m_cn_m_sc
#ub3lyp/6-311++G**  guess=read pop=nbo7read

 AEHB: H(-)...CN(-) pt.#m5, E = -93.2355578945(-4.86), <S**2>= 0.9067, R(CH)=1.3725
-2 1
H C   1   rch  x   2   1.    1
90. N   2   rcn   3   xcn   1
180.
 rcn
1.2023 xcn
39.25 rch
1.3725

$nbo file=h_m_cn_m_m5 archive $end

--Link1--
%mem=2gb
%nprocshared=8
%chk=h_m_cn_m_sc
#ub3lyp/6-311++G**  guess=read pop=nbo7read

 AEHB: H(-)...CN(-) pt.#m6, E = -93.2386507223(-6.80), <S**2>= 0.9509, R(CH)=1.3225
-2 1
H C   1   rch  x   2   1.    1
90. N   2   rcn   3   xcn   1
180.
 rcn
1.2042 xcn
40.03 rch
1.3225

$nbo file=h_m_cn_m_m6 archive $end

--Link1--
%mem=2gb
%nprocshared=8
%chk=h_m_cn_m_sc
#ub3lyp/6-311++G**  guess=read pop=nbo7read

 AEHB: H(-)...CN(-) pt.#m7, E = -93.2420089159(-8.91), <S**2>= 0.9893, R(CH)=1.2725
-2 1
```

```
H C   1   rch  x   2   1.    1
90. N   2   rcn   3   xcn   1
180.
 rcn
1.2041 xcn
41.71 rch
1.2725

$nbo file=h_m_cn_m_m7 archive $end

--Link1--
%mem=2gb
%nprocshared=8
%chk=h_m_cn_m_sc
#ub3lyp/6-311++G**  guess=read pop=nbo7read

 AEHB: H(-)...CN(-) pt.#m8, E = -93.2555310846(-17.39), <S**2>= 0.4403, R(CH)=1.2225

-2 1
H C   1   rch  x   2   1.    1
90. N   2   rcn   3   xcn   1
180.
 rcn
1.1537 xcn
90.00 rch
1.2225

$nbo file=h_m_cn_m_m8 archive $end

--Link1--
%mem=2gb
%nprocshared=8
%chk=h_m_cn_m_sc
#ub3lyp/6-311++G**  guess=read pop=nbo7read scf=qc

 AEHB: H(-)...CN(-) pt.#m9, E = -93.2620303056(-21.47), <S**2>= 0.2954, R(CH)=1.1725
-2 1
H C   1   rch  x   2   1.    1
90. N   2   rcn   3   xcn   1
180.
 rcn
1.1527 xcn
90.00 rch
1.1725

$nbo file=h_m_cn_m_m9 archive $end

--Link1--
%mem=2gb
%nprocshared=8
%chk=h_m_cn_m_sc
#ub3lyp/6-311++G**  guess=read pop=nbo7read scf=qc

 AEHB: H(-)...CN(-) pt.#m10, E = -93.2671750946(-24.70), <S**2>= 0.1152, R(CH)=1.1225

-2 1
H
C   1   rch  x   2   1.    1
90. N   2   rcn   3   xcn   1
180.
 rcn
1.1518 xcn
90.00 rch
1.1225

$nbo file=h_m_cn_m_m10 archive $end
```

```
--Link1--
%mem=2gb
%nprocshared=8
%chk=h_m_cn_m_sc
#ub3lyp/6-311++G**  guess=read pop=nbo7read scf=qc

 AEHB: H(-)...CN(-) pt.#m11, E = -93.2701684598(-26.58), <S**2>= 0.0000, R(CH)=1.0725

-2 1
H C   1   rch x   2   1.    1
90. N   2   rcn   3   xcn   1
180.
 rcn
1.1511 xcn
90.00 rch
1.0725

$nbo file=h_m_cn_m_m11 archive $end

--Link1--
%mem=2gb
%nprocshared=8
%chk=h_m_cn_m_sc
#ub3lyp/6-311++G**  guess=read pop=nbo7read scf=qc

 AEHB: H(-)...CN(-) pt.#m11, E = -93.2701684598(-26.58), <S**2>= 0.0000, R(CH)=1.0725
-2 1
H
C   1   rch  x   2   1.    1
90. N   2   rcn   3   xcn   1
180.
 rcn
1.1511 xcn
90.00 rch
1.0725

$nbo file=h_m_cn_m_m11 archive $end

--Link1--
%mem=2gb
%nprocshared=8
%chk=h_m_cn_m_sc
#ub3lyp/6-311++G**  guess=read pop=nbo7read scf=qc
 AEHB: H(-)...CN(-) pt.#m12, E = -93.2698973991(-26.41), <S**2>= 0.0000, R(CH)=1.0225

-2 1
H
C   1   rch  x   2   1.    1
90. N   2   rcn   3   xcn   1
180.
 rcn
1.1503 xcn
90.00 rch
1.0225 $nbo
file=h_m_cn
_m_m12
archive
$end

--Link1--
%mem=2gb
%nprocshared=8
%chk=h_m_cn_m_sc
#ub3lyp/6-311++G**  guess=read pop=nbo7read scf=qc

 AEHB: H(-)...CN(-) pt.#m13, E = -93.2651569285(-23.43), <S**2>= 0.0000, R(CH)=0.9725
-2 1
```

```
H
C   1   rch  x   2   1.    1
90. N   2   rcn   3   xcn   1
180.
 rcn
1.1495 xcn
90.00 rch
0.9725

$nbo file=h_m_cn_m_m13 archive $end

--Link1--
%mem=2gb
%nprocshared=8
%chk=h_m_cn_m_sc
#ub3lyp/6-311++G**  guess=read pop=nbo7read scf=qc

 AEHB: H(-)...CN(-) pt.#m14, E = -93.2544554419(-16.72), <S**2>= 0.0000, R(CH)=0.9225

-2 1
H
C   1   rch  x   2   1.    1
90. N   2   rcn   3   xcn   1
180.
 rcn
1.1486 xcn
90.00 rch
0.9225

$nbo file=h_m_cn_m_m14 archive $end

--Link1--
%mem=2gb
%nprocshared=8
%chk=h_m_cn_m_sc
#ub3lyp/6-311++G**  guess=read pop=nbo7read scf=qc

 AEHB: H(-)...CN(-) pt.#m15, E = -93.2358689847(-5.06), <S**2>= 0.0000, R(CH)=0.8725
-2 1
H
C   1   rch  x   2   1.    1
90. N   2   rcn   3   xcn   1
180.
 rcn
1.1477 xcn
90.00 rch
0.8725

$nbo file=h_m_cn_m_m15 archive $end

--Link1--
%mem=2gb
%nprocshared=8
%chk=h_m_cn_m_sc
#ub3lyp/6-311++G**  guess=read pop=nbo7read scf=qc

 AEHB: H(-)...CN(-) pt.#m16, E = -93.2068880068(+13.13), <S**2>= 0.0000, R(CH)=0.82225
-2 1
H
C   1   rch  x   2   1.    1
90. N   2   rcn   3   xcn   1
180.
 rcn
1.1468 xcn
90.00 rch
0.8225
```

```
$nbo file=h_m_cn_m_m16 archive $end

--Link1--
%mem=2gb
%nprocshared=8
%chk=h_m_cn_m_sc
#ub3lyp/6-311++G**  guess=read pop=nbo7read scf=qc

 AEHB: H(-)...CN(-) pt.#m17, E = -93.1033493172(+78.10), <S**2>= 0.0000, R(CH)=0.7725

-2 1
H
C   1   rch  x   2   1.    1
90. N   2   rcn   3   xcn   1
180.
 rcn
1.1456 xcn
90.00 rch
0.7725

$nbo file=h_m_cn_m_m17 archive $end

--Link1--
%mem=2gb
%nprocshared=8
%chk=h_m_cn_m_ts
#ub3lyp/6-311++G** guess=mix pop=nbo7read stable=opt

 AEHB: H(-)...CN(-) t.s., E(UB3LYP) =  -93.2278127967(0.00), <S**2>= 0.5590
R(CH)=1.6225, R(CN)=1.1928, TH(H-C-N)=126.27  nu: -1373,551,1931
 Vibrational temperatures:    793.28  2778.28
          (Kelvin)
 Zero-point correction=                           0.005655 (Hartree/Particle)
 Thermal correction to Energy=                    0.008677
 Thermal correction to Enthalpy=                  0.009622
Thermal correction to Gibbs Free Energy=        -0.015674
 Sum of electronic and zero-point Energies=           -93.222158
Sum of electronic and thermal Energies=              -93.219135
 Sum of electronic and thermal Enthalpies=            -93.218191
Sum of electronic and thermal Free Energies=         -93.243487
-2 1
H
C   1   1.6225 x   2
1.      1   90.
N   2   1.1928  3   36.27   1  180.

$nbo file=h_m_cn_m_ts archive $end

--Link1--
%mem=2gb
%nprocshared=8
%chk=h_m_cn_m_ts
#ub3lyp/6-311++G** guess=read pop=nbo7read opt=modredundant

 AEHB: H(-)...CN(-) t.s., E(UB3LYP) =  -93.2278127967(0.00), <S**2>= 0.5590
R(CH)=1.6225, R(CN)=1.1928, TH(H-C-N)=126.27  nu: -1373,551,1931
 Vibrational temperatures:    793.28  2778.28
          (Kelvin)
 Zero-point correction=                           0.005655 (Hartree/Particle)
 Thermal correction to Energy=                    0.008677
 Thermal correction to Enthalpy=                  0.009622
Thermal correction to Gibbs Free Energy=        -0.015674
 Sum of electronic and zero-point Energies=           -93.222158
 Sum of electronic and thermal Energies=              -93.219135
 Sum of electronic and thermal Enthalpies=            -93.218191
 Sum of electronic and thermal Free Energies=         -93.243487
Summary of Optimized Potential Surface Scan (add -93.0 to energies):
```

	1	2	3	4	5
Eigenvalues --	-0.227813	-0.228098	-0.228908	-0.230161	-0.231775
	(0.00)	(-0.18)	(-0.69)	(-1.47)	(-2.49)
	[0.5590]	[0.4659]	[0.3733]	[0.2852]	[0.2029]
R1	1.622500	1.672500	1.722500	1.772500	1.822500
R2	1.192800	1.191575	1.190759	1.189947	1.189321
A1	126.270000	125.665605	125.045032	124.556193	124.049267
	6	7	8	9	10
Eigenvalues --	-0.233673	-0.235792	-0.238076	-0.240447	-0.242853
	(-3.68)	(-5.01)	(-6.44)	(-7.93)	(-9.44)
	[0.1272]	[0.0576]	[closed-shell...]		
R1	1.872500	1.922500	1.972500	2.022500	2.072500
R2	1.188774	1.188299	1.187853	1.187203	1.186588
A1	123.625855	123.270469	123.001517	122.979494	123.042657
	11	12	13	14	15
Eigenvalues --	-0.245273	-0.247688	-0.250085	-0.252451	-0.254779
	(-10.96)	(-12.47)	(-13.97)	(-15.46)	(-16.92)
R1	2.122500	2.172500	2.222500	2.272500	2.322500
R2	1.186041	1.185542	1.185091	1.184685	1.184323
A1	123.175063	123.379952	123.656125	124.000414	124.408795
	16	17	18	19	20
Eigenvalues --	-0.257063	-0.259298	-0.261482	-0.263613	-0.265691
	(-18.35)	(-19.76)	(-21.13)	(-22.46)	(-23.77)
R1	2.372500	2.422500	2.472500	2.522500	2.572500
R2	1.183998	1.183707	1.183445	1.183209	1.182996
A1	124.877057	125.401372	125.978920	126.607835	127.286590
	21	22	23	24	25
Eigenvalues --	-0.267716	-0.269690	-0.271612	-0.273485	-0.275310
	(-25.04)	(-26.28)	(-27.48)	(-28.66)	(-29.80]
R1	2.622500	2.672500	2.722500	2.772500	2.822500
R2	1.182803	1.182628	1.182470	1.182324	1.182191
A1	128.012935	128.782294	129.588139	130.425753	131.293834
	26	27	28	29	30
Eigenvalues --	-0.277088	-0.278820	-0.280510	-0.282158	-0.283766

```
                         (-30.92)
          R1                2.872500          2.922500          2.972500          3.022500
3.072500
          R2                1.182068          1.181954          1.181848          1.181749
1.181655
          A1              132.191334        133.115977        134.065676        135.037661
136.027408
                            31
     Eigenvalues --          -0.285336
          R1                3.122500
          R2                1.181567
          A1              137.031112
 GradGradGradGradGradGradGradGradGradGradGradGradGradGradGradGradGradGradGrad

-2 1
H
C   1   1.6225 x   2
1.      1   90.
N   2   1.1928  3   36.27   1  180.

1 2 S  30 +0.05

$nbo file=h_m_cn_m_ts archive $end

--Link1--
--Link1--
**************************** BO(-)...BO(-) Input Files *****************************
%mem=2gb
%nprocshared=8
%chk=bo_m_bo_m_eq
#ub3lyp/6-311++G** stable=opt

 BO(-)...BO(-), guess

-2  1
                8                   0.364614   -2.000376   -0.000000
                5                  -0.000000   -0.815259   -0.000000
5                  0.000001    0.815259    0.000000                 8
-0.364615    2.000376    0.000000
$nbo file=bo_m_bo_m_eq archive $end

--Link1--
%mem=2gb
%nprocshared=8
%chk=bo_m_bo_m_eq
#ub3lyp/6-311++G** guess=read pop=nbo7read opt=calcall

 BO(-)...BO(-), -200.162177477(-6.50)[+91.64],<S**2>= 0.9407
nu: 211,227,345,601,1656,1831
 Vibrational temperatures:    303.42   326.31   496.19   864.85  2382.62
          (Kelvin)           2634.45
 Zero-point correction=                           0.011096 (Hartree/Particle)
 Thermal correction to Energy=                    0.015523
 Thermal correction to Enthalpy=                  0.016467
Thermal correction to Gibbs Free Energy=        -0.014002
 Sum of electronic and zero-point Energies=           -200.151081
 Sum of electronic and thermal Energies=              -200.146655
 Sum of electronic and thermal Enthalpies=            -200.145711
Sum of electronic and thermal Free Energies=         -200.176180
-2  1
                8                   0.364614   -2.000376   -0.000000
                5                  -0.000000   -0.815259   -0.000000
5                  0.000001    0.815259    0.000000                 8
-0.364615    2.000376    0.000000

$nbo file=bo_m_bo_m_eq archive $end
```

```
--Link1--
--Link1--
%mem=2gb
%nprocshared=8
%chk=bo_m_bo_m_ts
#b3lyp/6-311++G** pop=nbo7read

BO(-)...BO(-) t.s., E(RB3LYP) =  -200.151819263 (e-stable)
 nu: -299,64,216,331,1615,1627
 Vibrational temperatures:     91.84   311.37   476.56  2323.34  2341.22
(Kelvin)
 Zero-point correction=                           0.008779 (Hartree/Particle)
 Thermal correction to Energy=                    0.013342
 Thermal correction to Enthalpy=                  0.014286
Thermal correction to Gibbs Free Energy=        -0.017678
 Sum of electronic and zero-point Energies=           -200.143040
 Sum of electronic and thermal Energies=              -200.138478
 Sum of electronic and thermal Enthalpies=            -200.137533
Sum of electronic and thermal Free Energies=         -200.169497

-2 1
                8                   0.787374    1.969317    0.000000
                5                   0.787374    0.719667    0.000000
5                 -0.787374   -0.719667   -0.000000                8
-0.787374   -1.969317   -0.000000

$nbo file=bo_m_bo_m_ts archive $end

--Link1--

%mem=2gb
%nprocshared=8
%chk=bo_m_bo_m_eq_mp2
#uhf/6-311++G** stable=opt

 BO(-)...BO(-), guess

-2  1
                8                   0.364614   -2.000376   -0.000000
                5                  -0.000000   -0.815259   -0.000000
5                  0.000001    0.815259    0.000000                8
-0.364615    2.000376    0.000000

--Link1--
**************************** BO(-)...BO(-) MP2 Input Files ****************************
%mem=2gb
%nprocshared=8
%chk=bo_m_bo_m_eq_mp2
#ump2/6-311++G** guess=read pop=nbo7read density=current opt=calcall

 BO(-)...BO(-), EUMP2 = -0.19961786707969D+03(-3.55)[+97.72], (S**2,1)=  0.10069D+01
nu: 207,245,438,572,1591,1829
 Vibrational temperatures:    298.32   351.85   630.88   822.73  2288.72
          (Kelvin)           2631.73
 Zero-point correction=                           0.011122 (Hartree/Particle)
 Thermal correction to Energy=                    0.015453
 Thermal correction to Enthalpy=                  0.016397
Thermal correction to Gibbs Free Energy=        -0.014408
 Sum of electronic and zero-point Energies=           -199.606745
 Sum of electronic and thermal Energies=              -199.602414
 Sum of electronic and thermal Enthalpies=            -199.601470
Sum of electronic and thermal Free Energies=         -199.632275
-2  1
                8                  -0.230539    2.005448    0.000000
5                  0.259936    0.847734    0.000000                 5
0.000000   -0.804944    0.000000                8
0.068079   -2.032192    0.000000
```

```
$nbo file=bo_m_bo_m_eq_mp2 archive $end

--Link1--
%mem=2gb
%nprocshared=8
%chk=bo_m_bo_m_ts_mp2
#uhf/6-311++G** pop=nbo7read stable=opt

BO(-)...BO(-) t.s., guess

-2 1
                8                   0.783152    1.969494    0.000000
                 5                   0.783152    0.711957    0.000000
         5                  -0.783152   -0.711957    0.000000
         8                  -0.783152   -1.969494    0.000000

--Link1--
%mem=2gb
%nprocshared=8
%chk=bo_m_bo_m_ts_mp2
#mp2/6-311++G** pop=nbo7read opt=(ts,calcall)

BO(-)...BO(-) t.s., EUMP2 = -0.19961220769854D+03,
 nu: -321,59,224,324,1598,1611
 Vibrational temperatures:     84.56   322.45   469.27  2299.57  2318.02
          (Kelvin)
 Zero-point correction=                           0.008699 (Hartree/Particle)
 Thermal correction to Energy=                    0.013267
 Thermal correction to Enthalpy=                  0.014211
Thermal correction to Gibbs Free Energy=        -0.017812
 Sum of electronic and zero-point Energies=           -199.603509
 Sum of electronic and thermal Energies=              -199.598941
 Sum of electronic and thermal Enthalpies=            -199.597997
 Sum of electronic and thermal Free Energies=         -199.630019

-2 1
                8                   0.783152    1.969494    0.000000
                 5                   0.783152    0.711957    0.000000
         5                  -0.783152   -0.711957    0.000000
                8                  -0.783152   -1.969494    0.000000

$nbo file=bo_m_bo_m_ts_mp2 archive $end

--Link1--
**************************** BO(-)...BO(-) Segment Scan Input Files
****************************
%mem=2gb
%nprocshared=8
%chk=bo_m_bo_m_sc
#ub3lyp/6-311++G** nosymm guess=mix stable=opt

 BO(-)...BO(-), guess

-2  1
                8                   0.364614   -2.000376   -0.000000
                5                  -0.000000   -0.815259   -0.000000
                5                   0.000001    0.815259    0.000000
                8                  -0.364615    2.000376    0.000000

--Link1--
%mem=2gb
%nprocshared=8
%chk=bo_m_bo_m_sc
#ub3lyp/6-311++G** nosymm guess=read scf=qc opt=(modredundant,calcall)
 BO(-)...BO(-), -200.162177477(-6.50),<S**2>= 0.9407,
 BACK-SCAN FROM EQ
```

```
 R12     R23    R34   A123    A234      E           S^2    dE
------ ------ ------ ------ ------ ------------- ------ ------
1.2399 1.6305 1.2399 162.90 162.90 -200.162177476 0.9406  -6.50 1.2407
1.5805 1.2407 170.62 170.62 -200.161614522 0.9862  -6.15 1.2417 1.5305
1.2417 179.95 179.95 -200.159492914 1.0021  -4.82 1.2434 1.4805 1.2434
180.00 180.00 -200.154491232 1.0023  -1.68 1.2451 1.4305 1.2451 180.00
180.00 -200.145839135 1.0025  +3.75
1.2469 1.3805 1.2459 180.00 180.00 -200.132674900 1.0026 +12.01
 Summary of Optimized Potential Surface Scan (add -200.0 to energies):
                               1                 2                 3                 4
5
     Eigenvalues --           -0.162177         -0.161728         -0.160608         -0.159093
-0.157404
                               (-6.50)           (-6.22)           (-5.51)           (-4.56)
(-3.50)
           R1                 1.239938          1.239546          1.239903          1.240379
1.240992
           R2                 1.630518          1.680518          1.730518          1.780518
1.830518
           R3                 1.239938          1.239546          1.239903          1.240379
1.240991
           A1               162.898887        158.010383        154.035070        150.596823
147.431847
           A2               162.898799        158.010394        154.035102        150.596929
147.432374
           D1               180.000000        180.000000        180.000000        180.000000
180.000000
                               6                 7                 8                 9
10
     Eigenvalues --           -0.155719         -0.154192         -0.152971         -0.152290
-0.151945
                               (-2.45)           (-1.49)           (-0.72)           (-0.30)
(-0.08)
           R1                 1.242174          1.243709          1.246170          1.249854
1.249642
           R2                 1.880518          1.930518          1.980518          2.030518
2.080518
           R3                 1.242173          1.243709          1.246169          1.249855
1.249642
           A1               144.513234        141.792000        138.885704        135.750072
134.089641
           A2               144.513880        141.792567        138.885986        135.748961
134.088952
           D1               180.000000        180.000000        180.000000        180.000000
180.000000
                              11                12                13                14
15
     Eigenvalues --           -0.151820         -0.151919         -0.152239         -0.152765
-0.153481
                               (0.00)            (-0.06)           (-0.26)            -0.59)
(-1.04)
           R1                 1.249744          1.249627          1.249437          1.249169
1.248837
           R2                 2.130518          2.180518          2.230518          2.280518
2.330518
           R3                 1.249745          1.249627          1.249437          1.249169
1.248837
           A1               132.571061        131.104323        129.751425        128.483037
127.291892
           A2               132.570155        131.103573        129.750975        128.482882
127.291953
           D1               180.000000        180.000000        180.000000        180.000000
180.000000
                              16                17                18                19
20
     Eigenvalues --           -0.154364         -0.155392         -0.156542         -0.157793
-0.159123
```

	(-1.60)	(-2.24)	(-2.96)	(-3.75)	(-4.58)
R1	1.248457	1.248041	1.247602	1.247152	1.246698
R2	2.380518	2.430518	2.480518	2.530518	2.580518
R3	1.248457	1.248041	1.247602	1.247152	1.246698
A1	126.172128	125.118971	124.128427	123.197135	122.322268
A2	126.172308	125.119187	124.128624	123.197286	122.322365
D1	180.000000	180.000000	180.000000	180.000000	180.000000
	21	22	23	24	25
Eigenvalues --	-0.160514	-0.161950	-0.163417	-0.164903	-0.166397
	(-5.46)	(-6.36)	(-7.27)	(-8.21)	(-9.15)
R1	1.246249	1.245810	1.245385	1.244979	1.244592
R2	2.630518	2.680518	2.730518	2.780518	2.830518
R3	1.246249	1.245810	1.245385	1.244979	1.244592
A1	121.501455	120.732722	120.014505	119.345227	118.723280
A2	121.501504	120.732736	120.014497	119.345209	118.723260
D1	180.000000	180.000000	180.000000	180.000000	180.000000
	26	27	28	29	30
Eigenvalues --	-0.167891	-0.169377	-0.170851	-0.172308	-0.173744
	(-10.09)	(-11.02)			
R1	1.244227	1.243883	1.243561	1.243260	1.242979
R2	2.880518	2.930518	2.980518	3.030518	3.080518
R3	1.244227	1.243883	1.243561	1.243260	1.242979
A1	118.147307	117.615956	117.127829	116.681418	116.275350
A2	118.147290	117.615946	117.127825	116.681421	116.275350
D1	180.000000	180.000000	180.000000	180.000000	180.000000
	31	32	33	34	35
Eigenvalues --	-0.175158	-0.176548	-0.177912	-0.179250	-0.180562
R1	1.242719	1.242476	1.242250	1.242041	1.241847
R2	3.130518	3.180518	3.230518	3.280518	3.330518
R3	1.242719	1.242476	1.242250	1.242041	1.241847
A1	115.907232	115.575674	115.278698	115.014412	114.780736
A2	115.907232	115.575674	115.278698	115.014412	114.780736
D1	180.000000	180.000000	180.000000	180.000000	180.000000
	36	37	38	39	40

```
     Eigenvalues --           -0.181848         -0.183108         -0.184344         -0.185554
-0.186741
           R1                  1.241666          1.241498          1.241341          1.241195
1.241058
           R2                  3.380518          3.430518          3.480518          3.530518
3.580518
           R3                  1.241666          1.241498          1.241341          1.241195
1.241058
           A1                114.575584        114.396904        114.242671        114.110832
113.999417
           A2                114.575584        114.396904        114.242671        114.110832
113.999417
           D1                180.000000        180.000000        180.000000        180.000000
180.000000
                              41
     Eigenvalues --           -0.187905
           R1                  1.240930
           R2                  3.630518
           R3                  1.240930
           A1                113.906662
           A2                113.906662
           D1                180.000000
 GradGradGradGradGradGradGradGradGradGradGradGradGradGradGradGradGradGrad

-2  1
O  0.  0.  2.00695
B  0.  0.  0.76525
B  0.  0. -0.76525
O  0.  0. -2.00695

2 3  S  10 -0.05

--Link1--
--Link1--
%mem=2gb
%nprocshared=8
%chk=bo_m_bo_m_sc
#ub3lyp/6-311++G** stable=opt

 BO(-)...BO(-), guess

-2  1
                8                  0.364614   -2.000376   -0.000000
                5                 -0.000000   -0.815259   -0.000000
5                  0.000001    0.815259    0.000000                 8
-0.364615    2.000376    0.000000

--Link1--
%mem=2gb
%nprocshared=8
%chk=bo_m_bo_m_sc
#ub3lyp/6-311++G** guess=read  opt=(modredundant)

 BO(-)...BO(-), -200.162177477(-6.50),<S**2>= 0.9407, backward scan

-2  1
                8                  0.364614   -2.000376   -0.000000
                5                 -0.000000   -0.815259   -0.000000
5                  0.000001    0.815259    0.000000                 8
-0.364615    2.000376    0.000000

2 3  S  20 -0.05

--Link1--
--Link1--
%mem=2gb
%nprocshared=8
```

```
%chk=bo_m_bo_m_ts
#b3lyp/6-311++G** pop=nbo7read

BO(-)...BO(-) t.s., E(RB3LYP) =  -200.151819263 (e-stable)
 nu: -299,64,216,331,1615,1627
 Vibrational temperatures:     91.84   311.37   476.56  2323.34  2341.22
          (Kelvin)
 Zero-point correction=                           0.008779 (Hartree/Particle)
 Thermal correction to Energy=                    0.013342
 Thermal correction to Enthalpy=                  0.014286
Thermal correction to Gibbs Free Energy=        -0.017678
 Sum of electronic and zero-point Energies=           -200.143040
 Sum of electronic and thermal Energies=              -200.138478
 Sum of electronic and thermal Enthalpies=            -200.137533
 Sum of electronic and thermal Free Energies=         -200.169497

-2 1
                8                   0.787374    1.969317    0.000000
                5                   0.787374    0.719667    0.000000
5                 -0.787374   -0.719667   -0.000000                 8
-0.787374   -1.969317   -0.000000

$nbo file=bo_m_bo_m_ts archive $end

--Link1--
***************************** BO(-)...CN(-) Input Files *****************************
%mem=2gb
%nprocshared=8
%chk=bo_m_cn_m_eq
#ub3lyp/6-311++G** nosymm stable=opt

BO(-)...CN(-), starting guess

-2  1
                8                  -0.074688    1.821806    0.000000
5                   0.457037    0.681692   -0.000000
6                   0.000000   -0.767100   -0.000000                 7
                   -0.241098   -1.911472   -0.000000

$nbo file=bo_m_cn_m_eq archive $end

--Link1--
%mem=2gb
%nprocshared=8
%chk=bo_m_cn_m_eq
#ub3lyp/6-311++G** nosymm guess=read pop=nbo7read opt=calcall

BO(-)...CN(-), -192.881682863(-8.78)[+100.97], <S**2>= 0.7161
nu: 195,339,487,687,1653,2107
 Vibrational temperatures:    280.70   487.17   700.45   988.80  2378.65
          (Kelvin)           3031.37
 Zero-point correction=                           0.012457 (Hartree/Particle)
 Thermal correction to Energy=                    0.016587
 Thermal correction to Enthalpy=                  0.017531
Thermal correction to Gibbs Free Energy=        -0.012896
 Sum of electronic and zero-point Energies=           -192.869226
Sum of electronic and thermal Energies=              -192.865096
 Sum of electronic and thermal Enthalpies=            -192.864152
 Sum of electronic and thermal Free Energies=         -192.894579

-2  1
                8                  -0.035181    1.850119   -0.000000
5                   0.414326    0.676023   -0.000000
6                  -0.032349   -0.771196    0.000000                 7
                   -0.205545   -1.930019   -0.000000
$nbo file=bo_m_cn_m_eq archive nrt plot $end
```

```
--Link1--
--Link1--
%mem=2gb
%nprocshared=8
%chk=bo_m_cn_m_ts
#b3lyp/6-311++G** pop=nbo7read

BO(-)...CN(-) t.s., E(RB3LYP) =  -192.867689309 (e-stable)
 nu: -436,127,193,443,1508,159
 Vibrational temperatures:    182.60   277.24   637.29  2170.00  2962.98
(Kelvin)
 Zero-point correction=                           0.009865 (Hartree/Particle)
 Thermal correction to Energy=                    0.014229
Thermal correction to Enthalpy=                  0.015173  Thermal
correction to Gibbs Free Energy=        -0.016545
 Sum of electronic and zero-point Energies=           -192.857825
 Sum of electronic and thermal Energies=              -192.853460
 Sum of electronic and thermal Enthalpies=            -192.852516
Sum of electronic and thermal Free Energies=         -192.884235

-2  1
                8                  -0.452601    1.829800    0.000000
5                   0.478079    0.966075    0.000000                 6
0.000000   -0.869846    0.000000                7
0.175774   -2.035672    0.000000

$nbo file=bo_m_cn_m_ts archive nrt plot $end

--Link1--
%mem=2gb
%nprocshared=8
%chk=cn_m_bo_m_irc
#b3lyp/6-311++G** pop=nbo7read

NC(-)...BO(-), E(RB3LYP) =  -192.878060197(-6.51)
[+103.24]
nu: 224,380,461,686,`629,2144
 Vibrational temperatures:    322.89   547.13   663.88   986.83  2343.37
         (Kelvin)           3085.43
 Zero-point correction=                           0.012587 (Hartree/Parti$
 Thermal correction to Energy=                    0.016648
 Thermal correction to Enthalpy=                  0.017592
Thermal correction to Gibbs Free Energy=        -0.012788
 Sum of electronic and zero-point Energies=           -192.865473
Sum of electronic and thermal Energies=              -192.861412
 Sum of electronic and thermal Enthalpies=            -192.860468
Sum of electronic and thermal Free Energies=         -192.890848

-2  1
                7                  -0.000000   -0.210538   -0.162549
                6                  -0.000000    0.032661    0.981357
                5                  -0.000000    0.202205    2.490907
8                   0.000000    1.150288    3.317810

$nbo file=cn_m_bo_m_eq archive $end

--Link1--
**************************** BO(-)...CN(-) IRC Input Files ****************************
%mem=2gb
%nprocshared=8
%chk=cn_m_bo_m_irc
#b3lyp/6-311++G** pop=nbo7read

 CN(-)...BO(-) pt.#=m6, E = -192.877731015(-6.30), IRC=-2.06040
[+103.45]

-2 1
```

```
     7     0.126964     1.944376    -0.000000
     6     0.109938     0.773183    -0.000000
5    0.337259    -0.755285     0.000000     8
-0.425778    -1.762437     0.000000

$nbo file=cn_m_bo_m_m6 archive $end

--Link1--
%mem=2gb
%nprocshared=8
%chk=cn_m_bo_m_irc
#b3lyp/6-311++G** pop=nbo7read

 CN(-)...BO(-) pt.#=m5, E = -192.876323318(-5.42), IRC=-1.71779 [+104.33]

-2 1
7    0.135737     1.961684     0.000000      6     0.085575
     0.788239    -0.000000      5     0.364015    -0.781834
     0.000000
8    -0.433601    -1.770609    -0.000000

$nbo file=cn_m_bo_m_m5 archive $end

--Link1--
%mem=2gb
%nprocshared=8
%chk=cn_m_bo_m_irc
#b3lyp/6-311++G** pop=nbo7read

 CN(-)...BO(-) pt.#=m4, E = -192.874094205(-4.02), IRC=-1.37436
[+105.73]

-2 1
7     0.142775     1.980545     0.000000
     6     0.064613     0.805558     0.000000
     5     0.388859    -0.811185    -0.000000
8     -0.441127    -1.779921    -0.000000

$nbo file=cn_m_bo_m_m4 archive $end

--Link1--
%mem=2gb
%nprocshared=8
%chk=cn_m_bo_m_irc
#b3lyp/6-311++G** pop=nbo7read
[+107.28]

 CN(-)...BO(-) pt.#=m3, E = -192.871627171(-2.47), IRC=-1.03076

-2 1
7    0.149056     1.998995    -0.000000      6     0.045383
     0.824978     0.000000
     5     0.410920    -0.843835    -0.000000
8    -0.447383    -1.788171     0.000000

$nbo file=cn_m_bo_m_m3 archive $end

--Link1--
%mem=2gb
%nprocshared=8
%chk=cn_m_bo_m_irc
#b3lyp/6-311++G** pop=nbo7read

 CN(-)...BO(-) pt.#=m2, E = -192.869498383(-1.14), IRC=-0.68722
[+108.62]

-2 1
```

```
7    0.155168     2.017272    -0.000000      6     0.026684
     0.845036     0.000000
     5     0.431747    -0.877609    -0.000000
8    -0.453043    -1.795969     0.000000

$nbo file=cn_m_bo_m_m2 archive $end

--Link1--
%mem=2gb
%nprocshared=8
%chk=cn_m_bo_m_irc
#b3lyp/6-311++G** pop=nbo7read

 CN(-)...BO(-) pt.#=m1, E = -192.868133895(-0.28), IRC=-0.34368
[+109.47]

-2 1
     7     0.161142     2.035438     0.000000
6    0.008136     0.865699    -0.000000     5
0.451568    -0.912048     0.000000     8    -
0.457993    -1.803684     0.000000

$nbo file=cn_m_bo_m_m1 archive $end

--Link1--
%mem=2gb
%nprocshared=8
%chk=cn_m_bo_m_irc
#b3lyp/6-311++G** pop=nbo7read

CN(-)...BO(-) t.s., E(RB3LYP) =  -192.867689303(0.00), IRC=0.0000
[+109.75]]
nu: -436,127,193,443,1508,2060
 Vibrational temperatures:    182.38   277.16   637.32  2169.91  2963.35
          (Kelvin)
 Zero-point correction=                           0.009865 (Hartree/Parti$
 Thermal correction to Energy=                    0.014229
 Thermal correction to Enthalpy=                  0.015174
Thermal correction to Gibbs Free Energy=        -0.016546
 Sum of electronic and zero-point Energies=           -192.857824
 Sum of electronic and thermal Energies=              -192.853460
 Sum of electronic and thermal Enthalpies=            -192.852516
 Sum of electronic and thermal Free Energies=         -192.884235

-2 1
               7                   0.176053    2.035510    0.000000
               6                   0.000000    0.869767    0.000000
5                 0.477849   -0.965855   -0.000000                8
-0.452701   -1.829737   -0.000000

$nbo file=cn_m_bo_m_ts archive $end

--Link1--
%mem=2gb
%nprocshared=8
%chk=cn_m_bo_m_irc
#b3lyp/6-311++G** pop=nbo7read

 CN(-)...BO(-) pt.#=p1, E = -192.868088926(-0.25), IRC=0.34370
[+109.50]

-2 1
7    0.172659     2.071776    -0.000000      6    -0.026442
     0.910029     0.000000
     5     0.485825    -0.983468    -0.000000
8    -0.465714    -1.819596    -0.000000
```

```
$nbo file=cn_m_bo_m_p1 archive $end

--Link1--
%mem=2gb
%nprocshared=8
%chk=cn_m_bo_m_irc
#b3lyp/6-311++G** pop=nbo7read

 CN(-)...BO(-) pt.#=p2, E = -192.869184336(-0.94), IRC=0.68730
[+108.81]

-2 1
     7     0.178215     2.090266     0.000000
6    -0.042952     0.932950    -0.000000
     5     0.501456    -1.019429     0.000000
     8    -0.468948    -1.828232    -0.000000

$nbo file=cn_m_bo_m_p2 archive $end

--Link1--
%mem=2gb
%nprocshared=8
%chk=cn_m_bo_m_irc
#b3lyp/6-311++G** pop=nbo7read

 CN(-)...BO(-) pt.#=p3, E = -192.870827126(-1.97), IRC=1.03094
[+107.78]
-2 1
7    0.183577     2.108912    -0.000000      6    -0.058811     0.956490     0.000000
     5     0.515908    -1.055625    -0.000000
8    -0.471689    -1.837309     0.000000

$nbo file=cn_m_bo_m_p3 archive $end

--Link1--
%mem=2gb
%nprocshared=8
%chk=cn_m_bo_m_irc
#b3lyp/6-311++G** pop=nbo7read

 CN(-)...BO(-) pt.#=p4, E = -192.872881589(-3.26), IRC=1.37461
[+106.49]

-2 1
7     0.189024     2.127567     0.000000
     6    -0.074424     0.980301    -0.000000
     5     0.529839    -1.091844     0.000000
8     -0.474333    -1.846574    -0.000000

$nbo file=cn_m_bo_m_p4 archive $end

--Link1--
%mem=2gb
%nprocshared=8
%chk=cn_m_bo_m_irc
#b3lyp/6-311++G** pop=nbo7read

 CN(-)...BO(-) pt.#=p5, E = -192.875232196(-4.73), IRC=1.71827
[+105.02]

-2 1
7     0.194507     2.146337     0.000000
     6    -0.090019     1.004211    -0.000000
     5     0.543531    -1.127906     0.000000
8     -0.476858    -1.856123    -0.000000
```

```
$nbo file=cn_m_bo_m_p5 archive $end

--Link1--
%mem=2gb
%nprocshared=8
%chk=cn_m_bo_m_irc
#b3lyp/6-311++G** pop=nbo7read

 CN(-)...BO(-) pt.#=p6, E = -192.877782284(-6.33), IRC=2.06194
[+103.42]

-2 1
7    0.200017     2.165071    -0.000000      6    -0.105486
     1.028487     0.000000
     5     0.556566    -1.163964    -0.000000
8    -0.479049    -1.865920     0.000000

$nbo file=cn_m_bo_m_p6 archive $end

--Link1--
%mem=2gb
%nprocshared=8
%chk=cn_m_bo_m_irc
#b3lyp/6-311++G** pop=nbo7read

 CN(-)...BO(-) pt.#=p7, E = -192.880451246(-8.01), IRC=2.40562
[+101.74]

-2 1
7     0.205650     2.183645     0.000000
     6    -0.120974     1.053105    -0.000000
     5     0.569118    -1.199973     0.000000
8     -0.481000    -1.875864    -0.000000

$nbo file=cn_m_bo_m_p7 archive $end

--Link1--
%mem=2gb
%nprocshared=8
%chk=cn_m_bo_m_irc
#b3lyp/6-311++G** pop=nbo7read

 CN(-)...BO(-) pt.#=p8, E = -192.883174095(-9.72), IRC=2.74929
[+100.03]

-2 1
7    0.211379     2.202250    -0.000000      6    -0.136507
     1.077865    -0.000000
     5     0.581386    -1.235819    -0.000000
8    -0.482807    -1.886056     0.000000

$nbo file=cn_m_bo_m_p8 archive $end

--Link1--
%mem=2gb
%nprocshared=8
%chk=cn_m_bo_m_irc
#b3lyp/6-311++G** pop=nbo7read

 CN(-)...BO(-) pt.#=p9, E = -192.885901637(-11.43), IRC=3.09297
[+98.32]

-2 1
7    0.217169     2.220676    -0.000000      6    -0.152039
     1.103012     0.000000
     5     0.593113    -1.271612     0.000000
```

```
8    -0.484294    -1.896419    -0.000000

$nbo file=cn_m_bo_m_p9 archive $end

--Link1--
%mem=2gb
%nprocshared=8
%chk=cn_m_bo_m_irc
#b3lyp/6-311++G** pop=nbo7read

 CN(-)...BO(-) pt.#=p10, E = -192.888596488(-13.12), IRC=3.43665
[+96.63]

-2 1
7     0.223106     2.238802     0.000000
     6    -0.167679     1.128568    -0.000000
     5     0.604376    -1.307328    -0.000000
8     -0.485511    -1.906876     0.000000

$nbo file=cn_m_bo_m_p10 archive $end

--Link1--
%mem=2gb
%nprocshared=8
%chk=cn_m_bo_m_irc
#b3lyp/6-311++G** pop=nbo7read

 CN(-)...BO(-) pt.#=p11, E = -192.891230944(-14.77), IRC=3.78032
[+94.98]

-2 1
     7     0.229172     2.256909    -0.000000
6    -0.183427     1.154271     0.000000
     5     0.615385    -1.342841     0.000000
8    -0.486584    -1.917568    -0.000000  $nbo
file=cn_m_bo_m_p11 archive $end

--Link1--
%mem=2gb
%nprocshared=8
%chk=cn_m_bo_m_irc
#b3lyp/6-311++G** pop=nbo7read

 CN(-)...BO(-) pt.#=p12, E = -192.893787908(-16.38), IRC=4.12400
[+93.37]

-2 1
7     0.235266     2.274760     0.000000
     6    -0.199163     1.180408    -0.000000
     5     0.625884    -1.378275    -0.000000
8     -0.487339    -1.928417     0.000000

$nbo file=cn_m_bo_m_p12 archive $end

--Link1--
%mem=2gb
%nprocshared=8
%chk=cn_m_bo_m_irc
#b3lyp/6-311++G** pop=nbo7read

 CN(-)...BO(-) pt.#=p13, E = -192.896256327(-17.93), IRC=4.46768
[+91.82]

-2 1
7     0.241501     2.292216     0.000000
     6    -0.215020     1.207004    -0.000000
```

```
     5     0.635937    -1.413606     0.000000
8     -0.487821    -1.939334    -0.000000

$nbo file=cn_m_bo_m_p13 archive $end

--Link1--
%mem=2gb
%nprocshared=8
%chk=cn_m_bo_m_irc
#b3lyp/6-311++G** pop=nbo7read

 CN(-)...BO(-) pt.#=p14, E = -192.898629403(-19.42), IRC=4.81136
[+90.34]

-2 1
7     0.247855     2.309634     0.000000
     6    -0.230987     1.233744    -0.000000
     5     0.645773    -1.448712     0.000000
8     -0.488175    -1.950481     0.000000

$nbo file=cn_m_bo_m_p14 archive $end

--Link1--
%mem=2gb
%nprocshared=8
%chk=cn_m_bo_m_irc
#b3lyp/6-311++G** pop=nbo7read

 CN(-)...BO(-) pt.#=p15, E = -192.900906597(-20.84), IRC=5.15503
[+88.91]

-2 1
7    0.254168     2.326763    -0.000000      6    -0.246873
     1.260944     0.000000
     5     0.655131    -1.483729    -0.000000
8    -0.488225    -1.961783     0.000000

$nbo file=cn_m_bo_m_p15 archive $end

--Link1--
%mem=2gb
%nprocshared=8
%chk=cn_m_bo_m_irc
#b3lyp/6-311++G** pop=nbo7read

 CN(-)...BO(-) pt.#=p16, E = -192.903089175(-22.21), IRC=5.49872
[+87.54]

-2 1
7     0.260599     2.343432     0.000000
     6    -0.262857     1.288631    -0.000000
     5     0.664070    -1.518629     0.000000
8     -0.488015    -1.973127    -0.000000

$nbo file=cn_m_bo_m_p16 archive $end

--Link1--
%mem=2gb
%nprocshared=8
%chk=cn_m_bo_m_irc
#b3lyp/6-311++G** pop=nbo7read

 CN(-)...BO(-) pt.#=p17, E = -192.905178964(-23.53), IRC=5.84239
[+86.23]

-2 1
```

```
7    0.267109     2.360067    -0.000000      6    -0.278914
     1.316449     0.000000
     5     0.672835    -1.553312    -0.000000
8    -0.487702    -1.984688     0.000000

$nbo file=cn_m_bo_m_p17 archive $end

--Link1--
%mem=2gb
%nprocshared=8
%chk=cn_m_bo_m_irc
#b3lyp/6-311++G** pop=nbo7read

 CN(-)...BO(-) pt.#=p18, E = -192.907181746(-24.78), IRC=6.18607
[+84.97]

-2 1
7    0.273495     2.376414    -0.000000      6    -0.294795
     1.344728     0.000000
     5     0.681160    -1.587916     0.000000
8    -0.487108    -1.996399    -0.000000

$nbo file=cn_m_bo_m_p18 archive $end

--Link1--
%mem=2gb
%nprocshared=8
%chk=cn_m_bo_m_irc
#b3lyp/6-311++G** pop=nbo7read

 CN(-)...BO(-) pt.#=p19, E = -192.909102724(-25.99), IRC=6.52975
[+83.76]

-2 1
7     0.279962     2.392255     0.000000
     6    -0.310736     1.373504    -0.000000
     5     0.689098    -1.622406    -0.000000
8     -0.486274    -2.008116     0.000000

$nbo file=cn_m_bo_m_p19 archive $end

--Link1--
%mem=2gb
%nprocshared=8
%chk=cn_m_bo_m_irc
#b3lyp/6-311++G** pop=nbo7read

 CN(-)...BO(-) pt.#=p20, E = -192.910945576(-27.14), IRC=6.87343
[+82.61]

-2 1
7     0.286456     2.408086    -0.000000
     6    -0.326690     1.402386     0.000000
     5     0.696905    -1.656714     0.000000
8     -0.485363    -2.020030    -0.000000

$nbo file=cn_m_bo_m_p20 archive $end

--Link1--
--Link1--
%mem=2gb
%nprocshared=8
%chk=cn_m_bo_m
#b3lyp/6-311++G** pop=nbo7read
```

```
NC(-)...BO(-), E(RB3LYP) =  -192.878060197(-6.51) [+103.24
w.r.t. CN(-) + BO(-), -193.0425884]
nu: 224,380,461,686,`629,2144
 Vibrational temperatures:    322.89   547.13   663.88   986.83  2343.37
          (Kelvin)           3085.43
 Zero-point correction=                           0.012587 (Hartree/Parti$
 Thermal correction to Energy=                    0.016648
 Thermal correction to Enthalpy=                  0.017592
Thermal correction to Gibbs Free Energy=        -0.012788
 Sum of electronic and zero-point Energies=           -192.865473
Sum of electronic and thermal Energies=              -192.861412
 Sum of electronic and thermal Enthalpies=            -192.860468
 Sum of electronic and thermal Free Energies=         -192.890848

-2  1
                7                  -0.000000   -0.210538   -0.162549
                6                  -0.000000    0.032661    0.981357
                5                  -0.000000    0.202205    2.490907
8                  0.000000    1.150288    3.317810

$nbo file=cn_m_bo_m archive $end

--Link1--
%mem=2gb
%nprocshared=8
%chk=cn_m
#b3lyp/6-311++G** pop=nbo7read

CN(-), -92.8884777652 nu:
2122
 Vibrational temperatures:   3053.46
          (Kelvin)
 Zero-point correction=                           0.004835 (Hartree/Particle)
 Thermal correction to Energy=                    0.007196
 Thermal correction to Enthalpy=                  0.008140
Thermal correction to Gibbs Free Energy=        -0.014204
 Sum of electronic and zero-point Energies=            -92.883643
 Sum of electronic and thermal Energies=               -92.881282
 Sum of electronic and thermal Enthalpies=             -92.880338
 Sum of electronic and thermal Free Energies=          -92.902682

-1  1
C
N  1  1.1753

$nbo file=cn_m archive $end

--Link1--
%mem=2gb
%nprocshared=8 %chk=bo_m
#b3lyp/6-311++G**
pop=nbo7read freq

BO(-), -100.154110620
 nu: 1737
 Vibrational temperatures:   2498.68
          (Kelvin)
 Zero-point correction=                           0.003956 (Hartree/Particle)
 Thermal correction to Energy=                    0.006319
 Thermal correction to Enthalpy=                  0.007263
Thermal correction to Gibbs Free Energy=        -0.015239
 Sum of electronic and zero-point Energies=           -100.150154
 Sum of electronic and thermal Energies=              -100.147792
 Sum of electronic and thermal Enthalpies=            -100.146848
Sum of electronic and thermal Free Energies=         -100.169349

-1 1
```

```
B
O   1   1.2351

$nbo file=bo_m archive $end

--Link1--
**************************** BO(-)...CN(-) MP2 Input Files ****************************
%mem=2gb
%nprocshared=8
%chk=bo_m_cn_m_eq_mp2
#uhf/6-311++G** stable=opt

BO(-)...CN(-), starting guess

-2  1
                8                  -0.035181    1.850119   -0.000000
5                   0.414326    0.676023   -0.000000
6                   -0.032349   -0.771196    0.000000                 7
                    -0.205545   -1.930019   -0.000000

--Link1--
%mem=2gb
%nprocshared=8
%chk=bo_m_cn_m_eq_mp2
#ump2/6-311++G**  guess=read pop=nbo7read opt=calcall

BO(-)...CN(-), -0.19234972212210D+03(-10.40)[+192.52], (S**2,1)=  0.90609D+00
nu: 237,355,556,680,1563,2775
 Vibrational temperatures:    340.84   510.79   799.52   977.79  2248.89
          (Kelvin)           3992.50
 Zero-point correction=                           0.014045 (Hartree/Particle)
 Thermal correction to Energy=                    0.018050
 Thermal correction to Enthalpy=                  0.018994
Thermal correction to Gibbs Free Energy=        -0.011503
 Sum of electronic and zero-point Energies=          -192.335677
 Sum of electronic and thermal Energies=             -192.331672
 Sum of electronic and thermal Enthalpies=           -192.330728
Sum of electronic and thermal Free Energies=         -192.361225

-2  1
                8                  -0.033488   -1.798603    0.000000
                5                   0.594900   -0.690762    0.000000
6                   0.000000    0.775500    0.000000                 7
-0.386657    1.884234    0.000000

$nbo file=bo_m_cn_m_eq_mp2 archive $end

--Link1--
%mem=2gb
%nprocshared=8
%chk=bo_m_cn_m_ts_mp2
#uhf/6-311++G** guess=mix stable=opt

BO(-)...CN(-) t.s., guess
-2  1
                8                  -0.452601    1.829800    0.000000
5                   0.478079    0.966075    0.000000
6                   0.000000   -0.869846    0.000000
7                   0.175774   -2.035672    0.000000
$nbo file=bo_m_cn_m_ts_mp2 archive $end

--Link1--
%mem=2gb
%nprocshared=8
%chk=bo_m_cn_m_ts_mp2
#ump2/6-311++G** guess=read pop=nbo7read opt=(ts,calcall)
```

```
BO(-)...CN(-) t.s., -0.19233315227723D+03, (S**2,1)=  0.70939D+00
nu: -549,110,209,387,1509,1962
 Vibrational temperatures:    158.34   301.20   556.70  2171.29  2823.57
          (Kelvin)
 Zero-point correction=                           0.009518 (Hartree/Particle)
 Thermal correction to Energy=                    0.013940
 Thermal correction to Enthalpy=                  0.014884
Thermal correction to Gibbs Free Energy=        -0.017085
 Sum of electronic and zero-point Energies=           -192.323634
 Sum of electronic and thermal Energies=              -192.319212
 Sum of electronic and thermal Enthalpies=            -192.318268
Sum of electronic and thermal Free Energies=         -192.350237

-2  1
                8                   0.418642   -1.870289    0.000000
                5                  -0.530398   -1.027096    0.000000
6                  0.000000    0.904040    0.000000
7                 -0.099592    2.096222    0.000000
$nbo file=bo_m_cn_m_ts_mp2 archive $end

--Link1--
***************************** BO(-)...CN(-) Segment Scan Input Files
*****************************
%mem=2gb
%nprocshared=8
%chk=bo_m_cn_m_sc
#b3lyp/6-311++G** pop=nbo7read opt=modredundant

BO(-)...CN(-) t.s., E(RB3LYP) =  -192.867689309 (e-stable) [109.75]
nu: -436,127,193,443,1508,159
 Vibrational temperatures:    182.60   277.24   637.29  2170.00  2962.98
          (Kelvin)
 Zero-point correction=                           0.009865 (Hartree/Particle)
 Thermal correction to Energy=                    0.014229
 Thermal correction to Enthalpy=                  0.015173
Thermal correction to Gibbs Free Energy=        -0.016545
 Sum of electronic and zero-point Energies=           -192.857825
Sum of electronic and thermal Energies=              -192.853460
 Sum of electronic and thermal Enthalpies=            -192.852516
 Sum of electronic and thermal Free Energies=         -192.884235
FORWARD SCAN FROM TS TO LONG-RANGE
                               1                  2                  3                  4
5
     Eigenvalues --           -0.867689         -0.867962         -0.868700         -0.869810
-0.871218
                               (0.00)            (-0.17)            (-0.63)            (-1.33)
(-2.21)
          R1                 1.269719          1.267502          1.265105          1.262807
1.260832
          R2                 1.897147          1.947147          1.997147          2.047147
2.097147
          R3                 1.179002          1.180438          1.181417          1.182002
1.182369
          A1               118.267270        117.461038        116.713876        115.968325
115.241108
          A2               156.830144        153.764056        151.269119        149.261459
147.593390
          D1               180.000000        180.000000        180.000000        180.000000
180.000000
                               6                  7                  8                  9
10
     Eigenvalues --           -0.872858         -0.874678         -0.876632         -0.878680
-0.880788
                               (-3.24)            (-4.39)            (-5.61)            (-6.90)
(-8.22)
          R1                 1.258785          1.257102          1.255459          1.254004
1.252658
```

```
          R2                2.147147          2.197147          2.247147          2.297147
2.347147
          R3                1.182605          1.182658          1.182728          1.182678
1.182705
          A1              114.528155        113.855350        113.195306        112.585217
112.039707
          A2              146.277249        145.120848        144.229520        143.451452
142.954770
          D1              180.000000        180.000000        180.000000        180.000000
180.000000
                              11                12                13                14
15
     Eigenvalues --         -0.882930         -0.885081         -0.887225         -0.889345
-0.891432
                              (-9.56)          (-10.91)          (-12.26)
          R1                1.251451          1.250347          1.249342          1.248430
1.247605
          R2                2.397147          2.447147          2.497147          2.547147
2.597147
          R3                1.182564          1.182465          1.182362          1.182256
1.182148
          A1              111.489212        110.976925        110.497980        110.054689
109.647094
          A2              142.437057        142.068263        141.819780        141.674668
141.623053
          D1              180.000000        180.000000        180.000000        180.000000
180.000000
                              16                17                18                19
20
     Eigenvalues --         -0.893476         -0.895473         -0.897418         -0.899310
-0.901147
          R1                1.246858          1.246182          1.245568          1.245012
1.244505
          R2                2.647147          2.697147          2.747147          2.797147
2.847147
          R3                1.182040          1.181934          1.181830          1.181728
1.181630
          A1              109.274468        108.935741        108.629551        108.354321
108.108675
          A2              141.657219        141.770309        141.956110        142.209308
142.525251
          D1              180.000000        180.000000        180.000000        180.000000
180.000000
                              21
     Eigenvalues --         -0.902930
R1                  1.244045            R2
2.897147            R3
1.181534
          A1              107.891621
          A2              142.899346
          D1              180.000000
 GradGradGradGradGradGradGradGradGradGradGradGradGradGradGradGradGradGrad
-2  1
                8                 -0.452601    1.829800    0.000000
5                   0.478079    0.966075    0.000000
6                   0.000000   -0.869846    0.000000
7                   0.175774   -2.035672    0.000000  2 3 s 20 .05

--Link1--
--Link1--
%mem=2gb
%nprocshared=8
%chk=bo_m_cn_m_sc
#ub3lyp/6-311++G** nosymm stable=opt

BO(-)...CN(-), starting guess
```

```
-2  1
                8                  -0.074688    1.821806    0.000000
5                   0.457037    0.681692   -0.000000
6                   0.000000   -0.767100   -0.000000                 7
                    -0.241098   -1.911472   -0.000000

$nbo file=bo_m_cn_m_eq archive $end

--Link1--
%mem=2gb
%nprocshared=8
%chk=bo_m_cn_m_sc
#ub3lyp/6-311++G** nosymm guess=read opt=modredundant

BO(-)...CN(-), -192.881682863(-8.78)[+100.97], <S**2>= 0.7161 nu:
195,339,487,687,1653,2107
 Vibrational temperatures:    280.70   487.17   700.45   988.80  2378.65
          (Kelvin)           3031.37
 Zero-point correction=                           0.012457 (Hartree/Particle)
 Thermal correction to Energy=                    0.016587
 Thermal correction to Enthalpy=                  0.017531
Thermal correction to Gibbs Free Energy=        -0.012896
 Sum of electronic and zero-point Energies=           -192.869226
 Sum of electronic and thermal Energies=              -192.865096
 Sum of electronic and thermal Enthalpies=            -192.864152
 Sum of electronic and thermal Free Energies=         -192.894579
FORWARD SCAN FROM EQ TOWARD TS
  R1     R2     R3    A123   A234      energy       S^2    dE
------ ------ ------ ------ ------ -------------- ------ ------
1.2572 1.5146 1.1717 141.90 171.35 -192.881682863 0.7161 -8.78 1.2591
1.5646 1.1691 137.16 170.62 -192.880989959 0.6930 -8.35 1.2609 1.6146
1.1678 133.41 170.01 -192.879199609 0.6697 -7.22 1.2621 1.6646 1.1674
130.24 169.45 -192.876714414 0.6374 -5.66 1.2631 1.7146 1.1678 127.37
168.76 -192.873907611 0.5857 -3.90 1.2645 1.7646 1.1692 124.58 167.54
-192.871149796 0.4969 -2.17 1.2671 1.8146 1.1723 121.60 164.78 -
192.868886449 0.3190 -0.75 1.2711 1.8646 1.1778 118.86 159.00 -
192.867812921 0.0000 -0.08
1.2689 1.9146 1.1797 118.05 156.37 -192.867724593 0.0000 -0.02

-2  1
                8                  -0.035181    1.850119   -0.000000
        5                   0.414326    0.676023   -0.000000
6                  -0.032349   -0.771196    0.000000
7                  -0.205545   -1.930019   -0.000000

2 3 S 20 +0.05

--Link1--
%mem=2gb
%nprocshared=8
%chk=bo_m_cn_m_sc
#ub3lyp/6-311++G** nosymm stable=opt

BO(-)...CN(-), starting guess

-2  1
                8                  -0.074688    1.821806    0.000000
5                   0.457037    0.681692   -0.000000
6                   0.000000   -0.767100   -0.000000
7                   -0.241098   -1.911472   -0.000000
$nbo file=bo_m_cn_m_eq archive $end

--Link1--
%mem=2gb
%nprocshared=8
%chk=bo_m_cn_m_sc
#ub3lyp/6-311++G** nosymm guess=read opt=modredundant
```

```
BO(-)...CN(-), -192.881682863(-8.78)[+100.97], <S**2>= 0.7161
nu: 195,339,487,687,1653,2107
 Vibrational temperatures:    280.70   487.17   700.45   988.80  2378.65
          (Kelvin)           3031.37
 Zero-point correction=                           0.012457 (Hartree/Particle)
 Thermal correction to Energy=                    0.016587
 Thermal correction to Enthalpy=                  0.017531
Thermal correction to Gibbs Free Energy=        -0.012896
 Sum of electronic and zero-point Energies=           -192.869226
 Sum of electronic and thermal Energies=              -192.865096
 Sum of electronic and thermal Enthalpies=            -192.864152
 Sum of electronic and thermal Free Energies=         -192.894579
BACKWARD SCAN FROM EQ TOWARD WALL
  R1     R2     R3    A123   A234      energy       S^2    dE
------ ------ ------ ------ ------ -------------- ------ ------
1.2572 1.5146 1.1717 141.90 171.35 -192.881682863 0.7161 -8.78 1.2549
1.4646 1.1762 148.19 172.45 -192.880836371 0.7501 -8.25 1.2523 1.4146
1.1836 156.88 174.52 -192.877959244 0.8096 -6.44 1.2537 1.3646 1.1957
168.09 177.63 -192.872383722 0.9140 -2.95 1.2588 1.3146 1.2068 179.96
179.95 -192.862842875 1.0052 +3.04
1.2615 1.2646 1.2097 180.00 180.00 -192.846973944 1.0053 13.00

-2  1
                8                  -0.035181    1.850119   -0.000000
5                   0.414326    0.676023   -0.000000
6                   -0.032349   -0.771196    0.000000                 7
                    -0.205545   -1.930019   -0.000000

2 3 S 20 -0.05

--Link1--
**************************** CN(-)...CN(-) Input Files ****************************
%mem=2gb
%nprocshared=8
%chk=cn_m_cn_m_ts
#ub3lyp/6-311++G** guess=read pop=nbo7read

 CN(-)...CN(-) t.s., E(UB3LYP) = -185.533827496, <S**2>= 0.7061
nu: -979,123,235,497,1755,1872
 Vibrational temperatures:    177.36   337.52   715.25  2524.65  2692.97
(Kelvin)
 Zero-point correction=                           0.010209 (Hartree/Particle)
 Thermal correction to Energy=                    0.014470
 Thermal correction to Enthalpy=                  0.015415
Thermal correction to Gibbs Free Energy=        -0.015572
 Sum of electronic and zero-point Energies=           -185.523618
 Sum of electronic and thermal Energies=              -185.519357
 Sum of electronic and thermal Enthalpies=            -185.518413
 Sum of electronic and thermal Free Energies=         -185.549399

-2 1
                7                  -0.726506    1.734081    0.000000
6                   0.074707    0.828532    0.000000                 6
0.000000   -0.782200    0.000000                 7
0.662471   -1.773795    0.000000

$nbo file=cn_m_cn_m_ts archive $end

--Link1--
%mem=2gb
%nprocshared=8
%chk=cn_m_cn_m_eq
#ub3lyp/6-311++G** guess=read pop=nbo7read

 NC(-)...CN(-), E(UB3LYP) =  -185.545676527(-7.44)[+145.13], <S**2>= 1.0073
nu: 148,230,287,881,1737,2161; R(CC) = 1.3557
```

```
 Vibrational temperatures:    213.16   331.24   412.81  1267.92  2499.35
          (Kelvin)           3109.75
 Zero-point correction=                           0.012405 (Hartree/Particle)
 Thermal correction to Energy=                    0.016896
 Thermal correction to Enthalpy=                  0.017840
Thermal correction to Gibbs Free Energy=        -0.012279
 Sum of electronic and zero-point Energies=           -185.533272
 Sum of electronic and thermal Energies=              -185.528781
 Sum of electronic and thermal Enthalpies=            -185.527837
Sum of electronic and thermal Free Energies=         -185.557956

-2  1
                7                   0.277167    1.848062   -0.000000
                6                   0.000304    0.677850   -0.000000
                6                   0.000000   -0.677835    0.000000
7                  -0.277428   -1.848074    0.000000

$nbo file=cn_m_cn_m_eq archive $end

--Link1--
--Link1--
**************************** CN(-)...CN(-) IRC Input Files ****************************
%mem=2gb
%nprocshared=8
%chk=cn_m_cn_mirc
#b3lyp/6-311++G** pop=nbo7read

CN(-)...CN(-), pt.#m1, E = , R(CC)= , IRC=

-2 1

$nbo file=cn_m_cn_m_m1 archive $end

--Link1--
%mem=2gb
%nprocshared=8
%chk=cn_m_cn_mirc
#b3lyp/6-311++G** pop=nbo7read irc=(calcfc,recalc=3,maxpoints=30)

CN(-)...CN(-) t.s., E(RB3LYP) =  -185.526222159 (0.00),R(CC)=1.539834, IRC=0.0000 nu:
-853,182,251,585,1652,1843
 Vibrational temperatures:    261.83   361.69   841.93  2377.14  2651.01
(Kelvin)
 Zero-point correction=                           0.010282 (Hartree/Particle)
 Thermal correction to Energy=                    0.014361
 Thermal correction to Enthalpy=                  0.015305
Thermal correction to Gibbs Free Energy=        -0.015100
 Sum of electronic and zero-point Energies=           -185.515940
 Sum of electronic and thermal Energies=              -185.511862
 Sum of electronic and thermal Enthalpies=            -185.510917
Sum of electronic and thermal Free Energies=         -185.541322

-2 1
              7                  -0.553471    1.801981    0.000000
6                   0.000000    0.739637    0.000000                6
-0.113760   -0.795989    0.000000                7
0.650979   -1.753680    0.000000

$nbo file=cn_m_cn_m_ts archive $end

--Link1--
%mem=2gb
%nprocshared=8
%chk=cn_m_cn_mirc
#b3lyp/6-311++G** pop=nbo7read

CN(-)...CN(-), pt.#p1, E = -185.525762734(+!!!0.29), R(CC)= 1.543689, IRC=0.04989
```

```
-2 1
1          7           0       -0.552743    1.804558   -0.000000
2          6           0       -0.003061    0.739639    0.000000
3          6           0       -0.109450   -0.800379    0.000000
4          7           0        0.649160   -1.752506    0.000000

$nbo file=cn_m_cn_m_p1 archive $end

--Link1--
%mem=2gb
%nprocshared=8
%chk=cn_m_cn_mirc
#b3lyp/6-311++G** pop=nbo7read

CN(-)...CN(-), pt.#p2, E = -185.529782772, R(CC)= 1.603262, IRC=0.39835

-2 1
1          7           0       -0.565388    1.811528    0.000000
2          6           0        0.022301    0.767123   -0.000000
3          6           0       -0.126499   -0.829220    0.000000
4          7           0        0.654681   -1.758317    0.000000

$nbo file=cn_m_cn_m_p2 archive $end

--Link1--
%mem=2gb
%nprocshared=8
%chk=cn_m_cn_mirc
#b3lyp/6-311++G** pop=nbo7read

CN(-)...CN(-), pt.#p3, E = -185.534907296, R(CC)= 1.665569, IRC=0.74708

-2 1
1          7           0       -0.577172    1.817670    0.000000
2          6           0        0.046337    0.795918   -0.000000
3          6           0       -0.143174   -0.858834    0.000000
4          7           0        0.660156   -1.763756   -0.000000

$nbo file=cn_m_cn_m_p3 archive $end

--Link1--
%mem=2gb
%nprocshared=8
%chk=cn_m_cn_mirc
#b3lyp/6-311++G** pop=nbo7read

CN(-)...CN(-), pt.#p4, E = -185.541042020, R(CC)= 1.730917, IRC=1.09591

-2 1
1          7           0       -0.588017    1.823829   -0.000000
2          6           0        0.068439    0.826189   -0.000000       3          6
           0       -0.158403   -0.889800   -0.000000       4          7           0
           0.665113   -1.769320    0.000000

$nbo file=cn_m_cn_m_p4 archive $end

--Link1--
%mem=2gb
%nprocshared=8
%chk=cn_m_cn_mirc
#b3lyp/6-311++G** pop=nbo7read

CN(-)...CN(-), pt.#p5, E = -185.547933761, R(CC)= 1.797833, IRC=1.44474
-2 1
      1          7           0       -0.598283    1.830424   -0.000000
2          6           0        0.089069    0.857255    0.000000       3
6           0       -0.172745   -0.921412   -0.000000
```

```
      4          7           0        0.669989   -1.775446    0.000000
$nbo file=cn_m_cn_m_p5 archive $end

--Link1--
%mem=2gb
%nprocshared=8
%chk=cn_m_cn_mirc
#b3lyp/6-311++G** pop=nbo7read

CN(-)...CN(-), pt.#p6, E = -185.555281016, R(CC)= 1.866185, IRC=1.79359

-2 1
      1          7           0       -0.607857    1.837401   -0.000000
2         6          0        0.108071    0.889212    0.000000       3
6          0       -0.186082   -0.953645   -0.000000       4         7
0        0.674710   -1.782188    0.000000

$nbo file=cn_m_cn_m_p6 archive $end

--Link1--
%mem=2gb
%nprocshared=8
%chk=cn_m_cn_mirc
#b3lyp/6-311++G** pop=nbo7read

CN(-)...CN(-), pt.#p7, E = -185.562783221, R(CC)= 1.934904, IRC=2.14246

-2 1
      1          7           0       -0.617243    1.844931   -0.000000
2         6          0        0.126224    0.921478    0.000000       3
6          0       -0.198978   -0.985901   -0.000000       4         7
0        0.679590   -1.789726   -0.000000

$nbo file=cn_m_cn_m_p7 archive $end

--Link1--
%mem=2gb
%nprocshared=8
%chk=cn_m_cn_mirc
#b3lyp/6-311++G** pop=nbo7read

CN(-)...CN(-), pt.#p8, E = -185.570199394, R(CC)= 2.003918, IRC=2.49133

-2 1
      1          7           0       -0.626416    1.853014    0.000000
2         6          0        0.143660    0.953889    0.000000       3
6          0       -0.211477   -1.018310   -0.000000       4         7
0        0.684532   -1.797810   -0.000000

$nbo file=cn_m_cn_m_p8 archive $end

--Link1--
%mem=2gb
%nprocshared=8
%chk=cn_m_cn_mirc
#b3lyp/6-311++G** pop=nbo7read

CN(-)...CN(-), pt.#p9, E = -185.577354597, R(CC)= 2.073301, IRC=2.84020
-2 1
      1          7           0       -0.635265    1.861385   -0.000000
2         6          0        0.160283    0.986655    0.000000       3
6          0       -0.223571   -1.050803   -0.000000       4         7
0        0.689500   -1.806416   -0.000000

$nbo file=cn_m_cn_m_p9 archive $end

--Link1--
```

```
%mem=2gb
%nprocshared=8
%chk=cn_m_cn_mirc
#b3lyp/6-311++G** pop=nbo7read
CN(-)...CN(-), pt.#p10, E = -185.584134221, R(CC)= 2.142997, IRC=3.18908

-2 1
1          7           0       -0.643848    1.869911    0.000000
2          6           0        0.176232    1.019773   -0.000000
3          6           0       -0.235239   -1.083350   -0.000000       4          7           0
           0.694415   -1.815431    0.000000

$nbo file=cn_m_cn_m_p10 archive $end

--Link1--
%mem=2gb
%nprocshared=8
%chk=cn_m_cn_mirc
#b3lyp/6-311++G** pop=nbo7read

CN(-)...CN(-), pt.#p11, E = -185.590472598, R(CC)= 2.212828, IRC=3.53796

-2 1
      1          7           0       -0.652237    1.878800   -0.000000
2          6           0        0.191612    1.053017    0.000000       3
6           0       -0.246526   -1.116001    0.000000
      4          7           0        0.699297   -1.824828   -0.000000

$nbo file=cn_m_cn_m_p11 archive $end

--Link1--
%mem=2gb
%nprocshared=8
%chk=cn_m_cn_mirc
#b3lyp/6-311++G** pop=nbo7read

CN(-)...CN(-), pt.#p12, E = -185.596342792, R(CC)= 2.282983, IRC=3.88684

-2 1
1          7           0       -0.660237    1.887784    0.000000
2          6           0        0.206246    1.086618   -0.000000
3          6           0       -0.257370   -1.148795   -0.000000       4          7           0
           0.704048   -1.834504    0.000000

$nbo file=cn_m_cn_m_p12 archive $end

--Link1--
%mem=2gb
%nprocshared=8
%chk=cn_m_cn_mirc
#b3lyp/6-311++G** pop=nbo7read

CN(-)...CN(-), pt.#p13, E = -185.601742931, R(CC)= 2.353448, IRC=4.23572

-2 1
      1          7           0       -0.667895    1.896744   -0.000000
2          6           0        0.220210    1.120603    0.000000       3
6           0       -0.267727   -1.181707    0.000000
      4          7           0        0.708616   -1.844383   -0.000000

$nbo file=cn_m_cn_m_p13 archive $end

--Link1--
%mem=2gb
%nprocshared=8
%chk=cn_m_cn_mirc
#b3lyp/6-311++G** pop=nbo7read
```

```
CN(-)...CN(-), pt.#p14, E = -185.606686659, R(CC)= 2.423900, IRC=4.58460
-2 1
1          7           0       -0.675380    1.906057    0.000000
2          6           0        0.233682    1.154649   -0.000000
3          6           0       -0.277722   -1.214688   -0.000000
4          7           0        0.713121   -1.854609    0.000000

$nbo file=cn_m_cn_m_p14 archive $end

--Link1--
%mem=2gb
%nprocshared=8
%chk=cn_m_cn_mirc
#b3lyp/6-311++G** pop=nbo7read

CN(-)...CN(-), pt.#p15, E = -185.611202796, R(CC)= 2.494585, IRC=4.93348

-2 1
1          7           0       -0.682405    1.915484   -0.000000
2          6           0        0.246346    1.189018    0.000000       3          6
           0       -0.287176   -1.247846    0.000000
      4          7           0        0.717396   -1.865074   -0.000000

$nbo file=cn_m_cn_m_p15 archive $end

--Link1--
%mem=2gb
%nprocshared=8
%chk=cn_m_cn_mirc
#b3lyp/6-311++G** pop=nbo7read

CN(-)...CN(-), pt.#p16, E = , R(CC)= , IRC

-2 1

$nbo file=cn_m_cn_m_p16 archive $end

--Link1--
%mem=2gb
%nprocshared=8
%chk=cn_m_cn_mirc
#b3lyp/6-311++G** pop=nbo7read

CN(-)...CN(-), pt.#p17, E = , R(CC)= , IRC

-2 1

$nbo file=cn_m_cn_m_p17 archive $end

--Link1--
%mem=2gb
%nprocshared=8
%chk=cn_m_cn_mirc
#b3lyp/6-311++G** pop=nbo7read

CN(-)...CN(-), pt.#p18, E = , R(CC)= , IRC

-2 1

$nbo file=cn_m_cn_m_p18 archive $end

--Link1--
%mem=2gb
%nprocshared=8
%chk=cn_m_cn_mirc
```

```
#b3lyp/6-311++G** pop=nbo7read

CN(-)...CN(-), pt.#p19, E = , R(CC)= , IRC

-2 1

$nbo file=cn_m_cn_m_p19 archive $end

--Link1--
%mem=2gb
%nprocshared=8
%chk=cn_m_cn_mirc
#b3lyp/6-311++G** pop=nbo7read

CN(-)...CN(-), pt.#p20, E = , R(CC)= , IRC

-2 1

$nbo file=cn_m_cn_m_p20 archive $end

--Link1--
--Link1--
%mem=2gb
%nprocshared=8
%chk=cn_m_cn_m
#b3lyp/6-311++G** pop=nbo7read

NC(-)...CN(-), E(RB3LYP) =  -185.534077878 (-4.93) [+]
nu: 213,402,497,873,1854,2051
 Vibrational temperatures:    306.66   578.16   715.76  1255.70  2667.81
          (Kelvin)           2951.28
 Zero-point correction=                          0.013420 (Hartree/Particle)
 Thermal correction to Energy=                   0.017388
 Thermal correction to Enthalpy=                 0.018332
Thermal correction to Gibbs Free Energy=        -0.011313
 Sum of electronic and zero-point Energies=           -185.520658
Sum of electronic and thermal Energies=              -185.516690
 Sum of electronic and thermal Enthalpies=            -185.515746
Sum of electronic and thermal Free Energies=         -185.545391

-2  1
                7                  -0.000000   -0.248864   -0.110064
                6                  -0.000000    0.055566    1.031335
6                   0.000000    0.319059    2.371234                 7
0.000000    1.048856    3.335020

$nbo file=cn_m_cn_m archive $end

--Link1--
--Link1--
**************************** CN(-)...CN(-) MP2 Input Files ****************************
%mem=5gb
%nprocshared=8
%chk=junk_mp2.chk
#uhf/6-311++G** guess=mix scf=qc stable=opt

 NC(-)...CN(-) guess for t.s. search
 R(CC) = 1.68

-2 1
N C  1  r12 x  2  1.
1   90.
C  2  r23   3   c123  1   180. x
4  1.    2   90.   3   180. N  4
r34   5   c234  2   180.
 r12
1.2044  r34
```

```
1.2044 c123
47.74  c234
47.75   r23
1.68

--Link1--
%mem=2gb
%nprocshared=8
%chk=junk_mp2.chk
#ump2/6-311++G** guess=read pop=nbo7read scf=qc nosymm

 NC(-)...CN(-) t.s. (open-shell), -0.18500695200544D+03(0.00)[+163.94], S^2=0.6297
R(CC) = 1.6642
 nu: -1166,57,271,420,1983,2003
 Vibrational temperatures:     82.47   389.30   604.37  2853.09  2882.30
          (Kelvin)

 Zero-point correction=                           0.010785 (Hartree/Particle)
 Thermal correction to Energy=                    0.015187
 Thermal correction to Enthalpy=                  0.016132
Thermal correction to Gibbs Free Energy=        -0.015489
 Sum of electronic and zero-point Energies=           -184.996167
 Sum of electronic and thermal Energies=              -184.991765
 Sum of electronic and thermal Enthalpies=            -184.990820
 Sum of electronic and thermal Free Energies=         -185.022441

-2 1
N C  1  r12 x  2  1.    1   90.
C  2  r23   3   c123  1   180.
x  4  1.    2   90.   3   180.
N  4  r34   5   c234  2   180.
 r12
1.1937  r34
1.1937 c123
54.32  c234
54.33   r23
1.6642

$nbo file=cn_m_cn_m_ts_mp2 archive $end

--Link1--
%mem=2gb
%nprocshared=8
%chk=junk_mp2.chk
#ump2/6-311++G** pop=nbo7read scf=qc nosymm opt=(calcall)

 NC(-)...CN(-) eq. (closed-shell), -0.18502720241847D+03(-12.71)[+151.23]
R(CC) = 1.3810
 nu: 248,489,528,896,1909,2597
 Vibrational temperatures:    356.23   703.92   759.52  1288.99  2747.07
          (Kelvin)          3736.42
 Zero-point correction=                           0.015188 (Hartree/Particle)
 Thermal correction to Energy=                    0.019003
 Thermal correction to Enthalpy=                  0.019947
Thermal correction to Gibbs Free Energy=        -0.009570
 Sum of electronic and zero-point Energies=           -185.012014
Sum of electronic and thermal Energies=              -185.008200
 Sum of electronic and thermal Enthalpies=            -185.007255
 Sum of electronic and thermal Free Energies=         -185.036772

-2 1
N C  1  r12 x  2  1.    1   90.
C  2  r23   3   c123  1   180.
x  4  1.    2   90.   3   180.
N  4  r34   5   c234  2   180.
 r12
1.1882  r34
1.2193 c123
```

```
90.00  c234
59.61   r23
1.3810

$nbo file=cn_m_cn_m_eq_mp2 archive $end

--Link1--
**************************** CN(-)...CN(-) Segment Scan Input Files
****************************
%mem=2gb
%nprocshared=8
%chk=cn_m_cn_m_sc
#ub3lyp/6-311++G** guess=mix stable=opt

 CN(-)...CN(-) scan guess

-2 1
N
C   1    1.1928 x   2    1.0     1
90. C   2    1.5625  3    90.   1
180. x   4    1.0     2    90.   3
0. N   4    1.2091  5    90.   2
180.

--Link1--
%mem=2gb
%nprocshared=8
%chk=cn_m_cn_m_sc
#ub3lyp/6-311++G** guess=read opt=(modredundant,calcall,maxcycles=50) scf=qc

 CN(-)...CN(-) t.s., E(UB3LYP) = -185.533827496, <S**2>= 0.7061
  R12    R23    R34   A123   A234      energy     <S**2>   dE
------ ------ ------ ------ ------ -------------- ------ ------
1.2110 1.6625 1.2110 133.75 133.65 -185.536411431 0.1431  -1.62 1.2109
1.6525 1.2110 134.29 134.18 -185.535693660 0.2184  -1.17 1.2108 1.6425
1.2109 134.80 134.74 -185.535047018 0.2861  -0.77
1.2107 1.6325 1.2108 135.36 135.29 -185.534467188 0.3481  -0.40 1.2025
1.6225 1.2113 138.76 135.17 -185.534004111 0.4998  -0.11
1.1930 1.6125 1.2093 143.42 135.83 -185.533827764 0.7003   0.00 1.1716
1.6025 1.2079 159.11 136.40 -185.534458702 1.0039  -0.40
1.1710 1.5925 1.2096 160.20 136.20 -185.535134237 1.0007  -0.82 1.1706
1.5825 1.2112 161.13 136.09 -185.535815439 0.9975  -1.25 1.1704 1.5725
1.2124 161.85 136.17 -185.536497718 0.9947  -1.68 1.1703 1.5625 1.2137
161.99 134.83 -185.537179825 0.9116  -2.10 1.1708 1.5125 1.2175 164.36
136.39 -185.540412687 0.9794  -4.13 1.1739 1.4625 1.2182 165.51 140.83
-185.543052676 0.9715  -5.79 1.1826 1.4125 1.2138 165.69 149.53 -
185.544771419 0.9773  -6.87 1.2022 1.3625 1.2021 165.43 165.43 -
185.545658248 1.0073  -7.42 1.2053 1.3125 1.2053 179.99 180.00 -
185.544715115 1.0075  -6.83 1.2079 1.2625 1.2079 180.00 180.00 -
185.538897753 1.0078  -3.18 1.2109 1.2125 1.2109 180.00 180.00 -
185.525706731 1.0080   5.10
1.2144 1.1625 1.2144 180.00 180.00 -185.502903488 1.0081  19.41

-2 1
N
C   1    1.1928 x   2    1.0     1
90. C   2    1.6625  3    90.   1
180. x   4    1.0     2    90.   3
0. N   4    1.2091  5    90.   2
180.

2 3 S 20 -0.01

--Link1--
--Link1--
%mem=2gb
%nprocshared=8
```

```
%chk=cn_m_cn_m_sc
#ub3lyp/6-311++G** guess=mix stable=opt

 CN(-)...CN(-) t.s., E(UB3LYP) = -185.533827496, <S**2>= 0.7061

-2 1
                7                  -0.726506    1.734081    0.000000
6                   0.074707    0.828532    0.000000
6                   0.000000   -0.782200    0.000000
7                   0.662471   -1.773795    0.000000
--Link1--
%mem=2gb
%nprocshared=8
%chk=cn_m_cn_m_sc
#ub3lyp/6-311++G** guess=read opt=(modredundant,maxcycles=50) scf=qc  CN(-)...CN(-) t.s.,
E(UB3LYP) = -185.533827496, <S**2>= 0.7061
 Summary of Optimized Potential Surface Scan (add -185.0 to energies):
                                1                 2                 3                 4
5
    Eigenvalues --          -0.533828         -0.536409         -0.540816         -0.545639
-0.550723
                              (0.00)           (-1.62)           (-4.39)           (-7.41)
(-10.60)
          R1                 1.192822          1.211052          1.209074          1.206097
1.203259
          R2                 1.612464          1.662464          1.712464          1.762464
1.812464
          R3                 1.209143          1.211037          1.209124          1.206177
1.203283
          A1               143.386952        133.594833        131.642116        130.261057
129.078571
          A2               135.825518        133.597420        131.650894        130.268863
129.080372
          D1               180.000000        180.000000        180.000000        180.000000
180.000000
                                6                 7                 8                 9
10
    Eigenvalues --          -0.555983         -0.561336         -0.566701         -0.572009
-0.577203
                            (-13.90)          (-17.26)
          R1                 1.200649          1.198284          1.196165          1.194282
1.192618
          R2                 1.862464          1.912464          1.962464          2.012464
2.062464
          R3                 1.200658          1.198290          1.196170          1.194286
1.192622
          A1               128.066667        127.207012        126.481668        125.875934
125.378070
          A2               128.067448        127.207583        126.482157        125.876402
125.378560
          D1               180.000000        180.000000        180.000000        180.000000
180.000000
                               11                12                13                14
15
    Eigenvalues --          -0.582238         -0.587084         -0.591720         -0.596133
-0.600318
          R1                 1.191155          1.189873          1.188753          1.187774
1.186918
          R2                 2.112464          2.162464          2.212464          2.262464
2.312464
          R3                 1.191159          1.189877          1.188757          1.187779
1.186923
          A1               124.978721        124.670376        124.446853        124.302870
124.233817
          A2               124.979283        124.671067        124.447736        124.304012
124.235291
```

```
           D1              180.000000        180.000000        180.000000        180.000000
180.000000
                              16                17                18                19
20
     Eigenvalues --          -0.604277         -0.608015         -0.611540         -0.614863
-0.617996
           R1                1.186167          1.185502          1.184910          1.184386
1.183928
           R2                2.362464          2.412464          2.462464          2.512464
2.562464
           R3                1.186172          1.185508          1.184917          1.184392
1.183934
           A1              124.235927        124.306976        124.446823        124.656027
124.932852
           A2              124.237806        124.309322        124.449658        124.659303
124.936437
           D1              180.000000        180.000000        180.000000        180.000000
180.000000
                              21                22                23                24
25
     Eigenvalues --          -0.620950         -0.623738         -0.626374         -0.628869
-0.631235
           R1                1.183533          1.183192          1.182896          1.182636
1.182403
           R2                2.612464          2.662464          2.712464          2.762464
2.812464
           R3                1.183538          1.183197          1.182900          1.182639
1.182406
           A1              125.271598        125.664889        126.106086        126.590114
127.113023
           A2              125.275287        125.668457        126.109341        126.592934
127.115358
           D1              180.000000        180.000000        180.000000        180.000000
180.000000
                              26                27                28                29
30
     Eigenvalues --          -0.633482         -0.635620         -0.637661         -0.639610
-0.641477
           R1                1.182194          1.182004          1.181831          1.181673
1.181528
           R2                2.862464          2.912464          2.962464          3.012464
3.062464
           R3                1.182196          1.182005          1.181832          1.181674
1.181529
           A1              127.671564        128.263012        128.884896        129.534761
130.210150
           A2              127.673427        128.264456        128.885997        129.535602
130.210809
           D1              180.000000        180.000000        180.000000        180.000000
180.000000
                              31
     Eigenvalues --          -0.643269
           R1                1.181394
           R2                3.112464
           R3                1.181395
           A1              130.908841
           A2              130.909383
           D1              180.000000

-2 1
                7                  -0.726506    1.734081    0.000000
6                   0.074707    0.828532    0.000000
6                   0.000000   -0.782200    0.000000
7                   0.662471   -1.773795    0.000000
2 3 S 30 +0.05

--Link1--
```

```
--Link1--
%mem=2gb
%nprocshared=8
%chk=cn_m_cn_m_eq
#ub3lyp/6-311++G** guess=read pop=nbo7read

 NC(-)...CN(-), E(UB3LYP) =  -185.545676527(-7.44), <S**2>= 1.0073
nu: 148,230,287,881,1737,2161
 Vibrational temperatures:    213.16   331.24   412.81  1267.92  2499.35
          (Kelvin)           3109.75
 Zero-point correction=                           0.012405 (Hartree/Particle)
 Thermal correction to Energy=                    0.016896
 Thermal correction to Enthalpy=                  0.017840
Thermal correction to Gibbs Free Energy=        -0.012279
 Sum of electronic and zero-point Energies=           -185.533272
 Sum of electronic and thermal Energies=              -185.528781
 Sum of electronic and thermal Enthalpies=            -185.527837
Sum of electronic and thermal Free Energies=         -185.557956
-2  1
                7                   0.277167    1.848062   -0.000000
                6                   0.000304    0.677850   -0.000000
6                   0.000000   -0.677835    0.000000
7                   -0.277428   -1.848074    0.000000

$nbo file=cn_m_cn_m_eq archive $end

--Link1--
--Link1--
```
